# Regioselective Synthesis of β,γ-Unsaturated Amides from Unactivated Alkenes

**DOI:** 10.1021/acs.joc.5c00093

**Published:** 2025-03-08

**Authors:** Sabela Vega-Ces, Bogdan R. Brutiu, Daniel Kaiser, Nuno Maulide

**Affiliations:** † Institute of Organic Chemistry, University of Vienna, Währinger Straße 38, 1090 Vienna, Austria

## Abstract

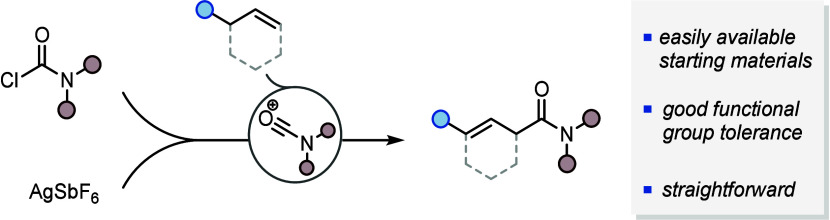

β,γ-Unsaturated amides are valuable substrates for downstream functionalization reactions but can be challenging to prepare. Herein, we introduce an approach featuring the regioselective addition of carbamoyl chlorides to unactivated alkenes, present its scope and limitations, and exemplify its synthetic utility.

Carboxamides have gained attention as important motifs in drug design^1−3^ (where they contribute to the stability, bioavailability, and conformational control of therapeutic compounds), in materials science (as high-stability polymers^4−6^ or biomedical materials^7−9^), and as useful ligands in catalysis.^10^ In particular, β,γ-unsaturated amides—despite being rarely found in nature—are considered valuable substrates for numerous functionalization reactions such as hydroborations,^11^ hydrochlorinations^12^ or formation of motifs such as chiral 1,4-dicarbonyls,^13,14^ 3-pyrrolin-2-ones^15^ and γ-lactams.^16^ However, their preparation can be challenging.^17^ Coupling the β,γ-unsaturated carboxylic acid precursor to the corresponding amine is widely reported (Top Left, Scheme 1a);^13,14,18−20^ however, the substrates are generally not commercially available.^21−23^ Transition-metal-catalyzed carbonylation represents a robust methodology for the synthesis of β,γ-unsaturated carbonyls.^24^ These reactions have evolved from transformations that required harsh conditions^25,26^ and had low atom economy, to efficient and versatile reactions conducted under mild conditions. Examples include the palladium-catalyzed carbonylation of allylamines developed by Huang (Top Right, Scheme 1a),^27^ as well as subsequent work by Beller (Bottom Left, Scheme 1a),^28^ who introduced a multicomponent version enabling the addition of alcohols or amines along with carbon monoxide to allyl chlorides, to yield β,γ-unsaturated esters and amides, respectively. These methods require prefunctionalized substrates and high-pressure-tolerant setups, and while they are well-reported for esters, they are generally underreported for amides.^29,30^ Furthermore, 1,5-HAT-mediated remote desaturation reactions, which combine transition-metal catalysis and photoinduced radical generation, have gained importance in recent years.^31−33^ For instance, Yu reported a pioneering photoinduced and palladium-catalyzed protocol that required prefunctionalized *O*-acyl hydroxamates (Bottom Right, Scheme 1a).^34^ The currently reported strategies leave space for the development of additional and complementary methods that employ readily available substrates in a single-step synthesis.

**Scheme 1:**
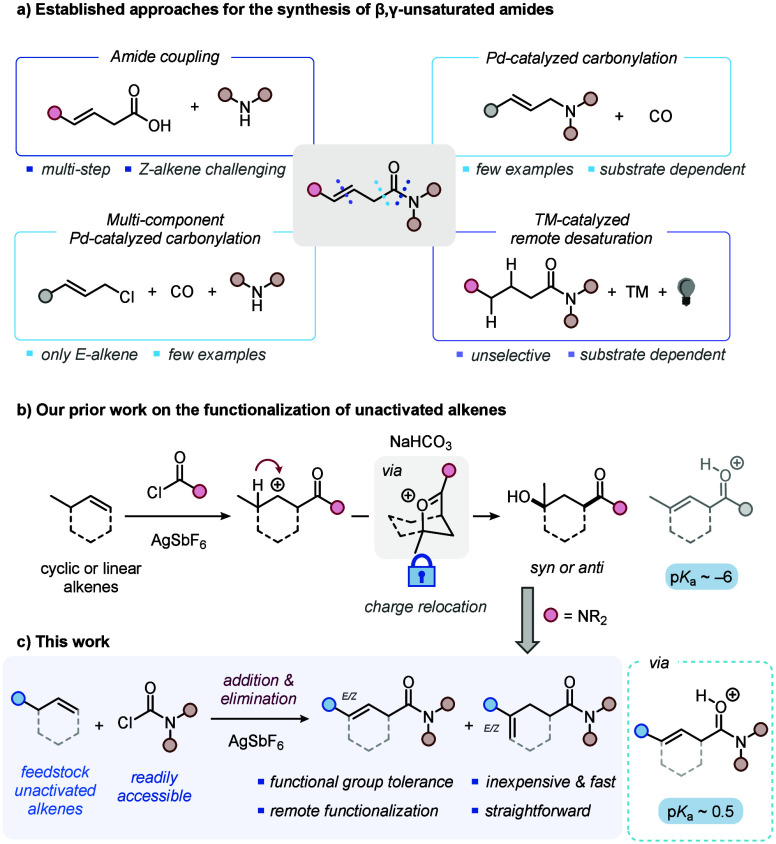
(a) Established Approaches for the Synthesis of β,γ-Unsaturated Amides, (b) Our Previous Work on the Functionalization of Unactivated Alkenes, (c) This Work: Synthesis of β,γ-Unsaturated Amides from Unactivated Alkenes

Our group recently disclosed a general method for the regioselective 1,3-difunctionalization of unactivated alkenes (Scheme 1b).^35^ Therein, the electrophilic addition of acylium ions to alkenes served as the basis for a selective strategy termed “charge relocation”—referring to a synthetic logic based on formal migration of incipient positive charge to a defined position, resulting in the formation of a bridged oxocarbenium intermediate. While we reported a range of different nucleophiles capable of opening this oxocarbenium intermediate, variations of the electrophilic reactant were not explored.

We initially hypothesized that transitioning from acyl chlorides to carbamoyl chlorides might provide access to the corresponding γ-hydroxy amides with relative *syn*-configuration. To our surprise, initial investigations revealed β,γ- and γ,δ-unsaturated amides as major products of this type of transformation (Scheme 1c).^36^ Here, we present the regioselective transformation of unactivated alkenes into β,γ-unsaturated amides under mild conditions and with good functional group tolerance.

Following the aforementioned initial observations, cyclohexene (**1a**) and *N,N-*diethylcarbamoyl chloride (**2a**) were chosen as model substrates for optimization of the reaction conditions (Table 1). Our initial conditions saw **2a** treated with silver hexafluoroantimonate in the presence of **1a** at 0 °C, forming a 14:1 regiosiomeric mixture of **3a** and **3a′** in 65% yield (entry 1). We subsequently found that a change to ambient temperature had minimal impact on the regioisomeric ratio, while leading to a significant increase in the yield, regardless of the concentration (entries 2 and 3). Other silver salts were also tested (entries 4 and 5), with silver triflate showing the best compromise between yield and regioselectivity (entry 4). However, the use of silver triflate negatively impacted the degree of product formation for some substrates, such as cyclopentenes and some linear olefins, due to the formation of side products resulting from triflate addition (see Supporting Information).

**Table 1:**
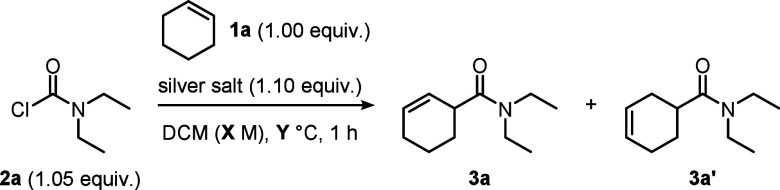

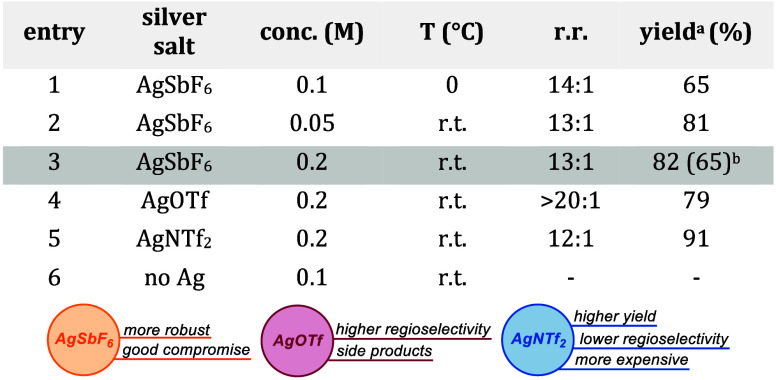
Optimization of Conditions

a Yield determined by NMR analysis of the crude reaction mixture, using mesitylene as an internal standard.

b Isolated yield. r.r. = regioisomeric ratio.

Although silver hexafluoroantimonate was selected as the standard salt, all three silver species were employed to explore the scope. A final control reaction lacking any silver additive did not lead to product formation, confirming this class of reagents’ key role for the activation of the carbamoyl chloride (entry 6, for a full table of optimization, see the Supporting Information).

Mechanistic considerations led us to the proposal shown in Scheme 2. Initially, the unactivated olefin attacks the highly electrophilic species **I**—generated by chloride abstraction—and furnishes β-amidic cation **II**.^35,37^ At this stage, we envision two alternative and potentially competing pathways. On the one hand, direct elimination can occur through a favorable 6-membered transition state, leading to the major regioisomeric product, β,γ-unsaturated amide **III**. On the other hand, intermediate **II** can also undergo a 1,2-hydride shift, forming **IV**. This places the carbocation at the γ-position (relative to the amide carbonyl) and allows for two distinct intramolecular elimination pathways, yielding the major (β,γ-unsaturated amide, **III**) and the minor (γ,δ-unsaturated amide, **V**) regioisomers.^38^ Amide-mediated deprotonation was additionally confirmed by ^13^C NMR experiments, which showed the protonated amide with a characteristic imidate peak at 179 ppm (see the Supporting Information for details).^39^

**Scheme 2:**
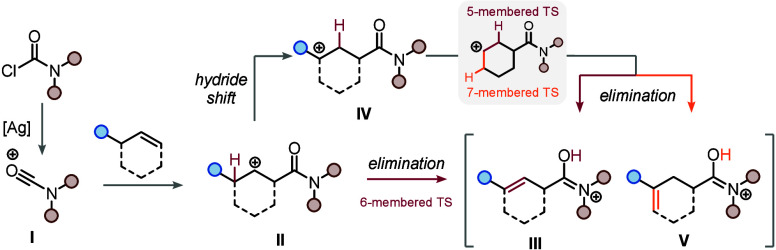
Proposed Mechanism

The optimized conditions (Table 1, entries 3–5) were applied to investigate the scope of this synthesis (Scheme 3), with initial investigations focused on variation of the carbamoyl chloride (**2**) and a range of cyclohexene substrates (**1**). We were pleased to find that, despite slight variations in yield and regioisomeric ratio, changes to the nature of the carbamoyl chloride were generally well tolerated (**3a**–**3j**). The use of an aniline-derived carbamoyl chloride, however, resulted in both a lower yield and a lower regioselectivity (**3h**).

**Scheme 3:**
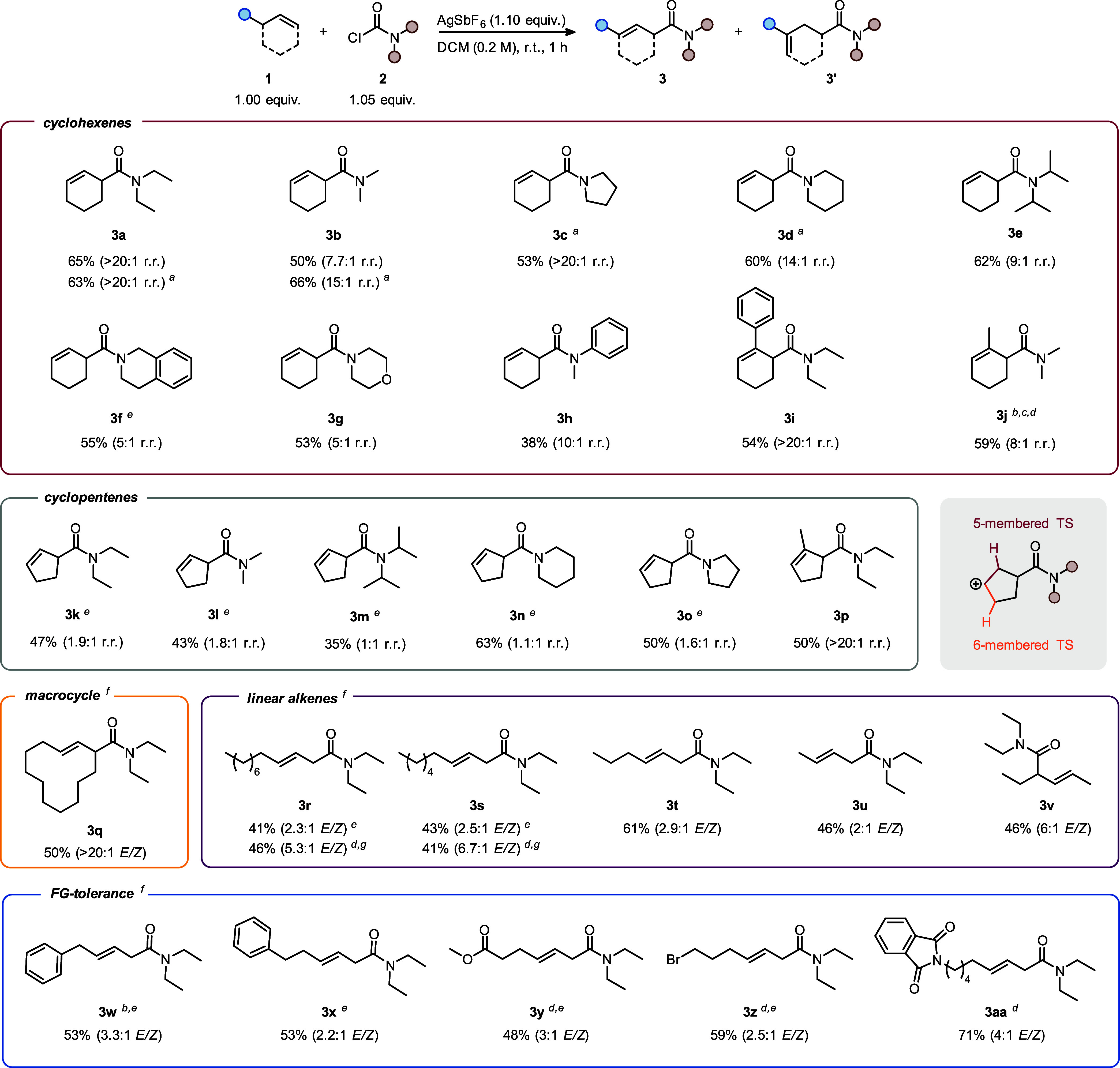
Substrate Scope AgOTf. AgNTf_2_. The minor regioisomer results from olefin isomerization and addition of the resulting methylenecyclohexane. 2.0 equiv of carbamoyl chloride and 2.1 equiv of AgSbF_6_ were used. The isomers were separable by column chromatography. The β,γ-unsaturated product was formed regioselectively. Yield determined by NMR analysis of the crude reaction mixture, using mesitylene as an internal standard. r.r. = regioisomeric ratio.

A possible explanation for this can be found in the decreased basicity of the amide, prolonging the lifetime of the β-amidic carbocation (cf. **II** in Scheme 2), thus facilitating the 1,2-hydride shift route. Similarly, sterically demanding substituents at nitrogen may also hamper direct elimination, resulting in similar outcomes (cf. **3e**). Substituted cyclohexenes might be expected to stabilize the β-amidic carbocation, disfavoring 1,2-hydride shifts and resulting in highly regioselective transformations. This is indeed the case for **3i**, where an activated olefin results in the regioselective formation of the β,γ-unsaturated amide, whereas methyl-substituted product **3j** was formed as an 8:1 mixture, favoring the desired product over a different regioisomer—a result that we ascribe to *in situ* alkene isomerization prior to carbamoylation (see the Supporting Information for details).

Cyclopentenes, in turn, show similar yields but poorer regioselectivities (**3k**–**3p**). Whereas, in general, the formation of the β,γ-unsaturated amide is still favored (**3k, 3l, 3o**); these compounds seemingly exhibit a higher tendency toward 1,2-hydride shift and subsequent δ-deprotonation. Notably, while cyclohexenes show notable selectivity (due to 5-membered transition-state elimination outcompeting the 7-membered congener) even from the intermediate after hydride-shift (see **IV**, Scheme 2), cyclopentenes see competition between 5- and 6-membered transition states, likely leading to significantly lower selectivity (inset, Scheme 3). Thus, whereas **3k, 3l** and **3o** still slightly favored β,γ-unsaturation, **3m** and **3n** were formed without regioselectivity. 1-Methylcyclopentene, on the other hand, restored selectivity for the formation of the β,γ-unsaturated product, delivering **3p** with excellent regioselectivity.

Unexpectedly, a mixture of *cis*- and *trans*-cyclododecene resulted in the fully regio- and stereoselective formation of **3q**. A diverse set of linear olefins was also investigated, all of them resulting in the formation of stereoisomeric *E*/*Z* mixtures of β,γ-unsaturated amides, which were formed with complete regioselectivity. Terminal alkenes afforded the desired products in yields ranging from 43% to 61% and stereoselectivities in the range of 2.1–3.8:1 (**3r**, **3s**, **3t**, and **3u**). Of note, the use of an excess of the carbamoyl chloride and the silver salt was found to result in higher stereoselectivity (**3r**, **3s**). An internal olefin was also found to undergo the reaction, with **3v** being formed in similar yield but with markedly higher stereoselectivity. Additional linear unsaturated amides bearing phenyl groups (**3w, 3x**), an ester (**3y**), a bromide (**3z**) and a phthalimide (**3aa**) were also formed, showcasing good functional group tolerance of our transformation.

As mentioned previously, β,γ-unsaturated amides are substrates for numerous functionalization reactions. In order to exemplify the utility of our method, a TEMPO oxidation,^13^ convergently transforming the *E*/*Z* stereoisomeric mixture **3ab** (without purification) into a single γ-oxidized-α,β-unsaturated amide (**4a**), was performed in a telescoped fashion (Scheme 4a).

**Scheme 4:**
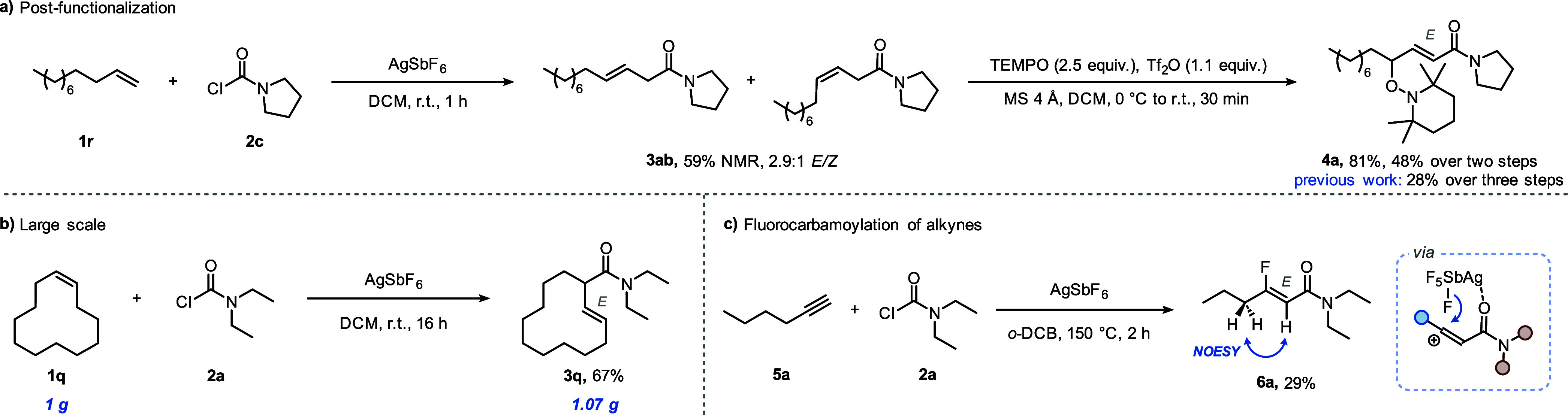
Method Applications

Notably, with our streamlined approach, **4a** was formed in only two steps from commercial starting materials and was obtained in an overall yield of 48%, a significant improvement from the previously reported three-step and 28%-yield synthesis. Pleasingly, our method is also amenable to gram-scale synthesis (Scheme 4b; 67% vs 50%, on 0.2 mmol scale).

Having explored the reactivity of alkenes under these conditions, we briefly turned our attention to the potential functionalization of alkynes (Scheme 4c). When treating alkyne **5a** with carbamoyl chloride **2a** under our reaction conditions, we were intrigued to observe the formation of β-fluoro-(*E*)-α,β-unsaturated amide **6a** as the only isolable product. While optimization of the initial observation was attempted (see the Supporting Information for further details), and 1,2-dichlorobenzene was established as the solvent allowing for the highest yield and cleanest reaction profile,^40^ the product was formed in only 29% yield. We propose that the exclusive formation of the *E*-configured alkene could result from coordination of silver to the amide carbonyl and *syn*-selective delivery of fluoride from the associated hexafluoroantimonate (see inset, Scheme 4c).

In conclusion, we have developed a straightforward method for the regioselective formation of β,γ-unsaturated amides. The novel retrosynthetic disconnection, along with good functional group tolerance and mild conditions, makes this reaction a valuable tool for synthesizing β,γ-unsaturated amides from feedstock unactivated alkenes and readily accessible carbamoyl chlorides. We have demonstrated synthetic utility by performing a large-scale reaction, as well as a telescoped and convergent postfunctionalization through TEMPO oxidation.

## Data Availability

The data underlying this study are available in the published article and its Supporting Information.

## Supporting Information Available

General experimental procedures, NMR spectra and full characterization of all substances not yet described in the literature, and details of mechanistic studies (PDF)
