# Supplementary material for: Regioselective Synthesis of β,γ-Unsaturated Amides from Unactivated Alkenes
Source: J Org Chem. 2025 Mar 8;90(11):4121–6. doi: 10.1021/acs.joc.5c00093 (PMC11934130; doi:10.1021/acs.joc.5c00093)
Supplement: Supplementary file 1 — jo5c00093_si_001.pdf [file jo5c00093_si_001.pdf]

Regioselective Synthesis of β,γ-Unsaturated Amides from Unactivated Alkenes

Sabela Vega-Ces[a], Bogdan R. Brutiu[a], Daniel Kaiser[a] and Nuno Maulide[a]*

[a] Institute of Organic Chemistry, University of Vienna, Währinger Straße 38, 1090 Vienna (Austria)
E-Mail: nuno.maulide@univie.ac.at, Homepage: http://maulide.univie.ac.at

Table of Contents

1. General Information S2
2. Optimization S3
   2.1. Optimization Table S3
   2.2. Triflate Adduct S4
3. Mechanistic Experiments S5
   3.1. Intramolecular Deprotonation by the Amide S5
   3.2. Evaluating Re-protonation/Elimination Route S8
4. Problematic/Failed Substrates S8
5. β-Fluoro-α,β-Unsaturated Amides Synthesis from Alkynes S9
6. Chloroformates S10
7. Experimental Procedures S11
   7.1. Synthesis of Starting Materials S11
      7.1.1. Synthesis of Non-Commercially Available Olefins & Amines S11
      7.1.2. General Procedure A: Synthesis of Carbamoyl Chlorides by Phosgenation S11
   7.2. Synthesis of β,γ-Unsaturated Amides from Unactivated Alkenes S16
   7.3. Further Functionalization S56
      7.3.1. Synthesis of α,β-Unsaturated γ-OTMP Amides S56
      7.3.2. Synthesis of additional α,β-Unsaturated γ-OTMP Amides S58
      7.3.3. Synthesis of β-Fluoro-α,β-Unsaturated Amides S59
8. NMR Spectra S61
9. References S107

1. General Information

Unless otherwise stated, all glassware was flame-dried before use and all reactions were performed under an atmosphere of argon. All solvents were distilled from appropriate drying agents prior to use. All reagents were used as received from commercial suppliers unless otherwise stated. Reaction progress was monitored by thin layer chromatography (TLC) performed on aluminum plates coated with silica gel F254 with 0.2 mm thickness. Chromatograms were visualized by fluorescence quenching with UV light at 254 nm or by staining using potassium permanganate. Flash column chromatography was performed using silica gel 60 (230-400 mesh, Merck and co.). Neat infrared spectra were recorded using a Perkin-Elmer Spectrum 100 FT-IR spectrometer. Wavenumbers ($\tilde{\nu}_{max}$) are reported in cm^{-1}. Mass spectra were obtained on a maXis UHR ESI-Qq-TOF mass spectrometer (Bruker Daltonics, Bremen, Germany) in the positive and/or negative ion mode by direct infusion, using electrospray ionization (ESI) and electron ionization–mass spectrometry (EI–MS). The sum formulas of the detected ions were determined using Bruker Compass DataAnalysis 4.1 based on the mass accuracy ($\Delta m/z \leq 5$ ppm) and isotopic pattern matching (SmartFormula algorithm). Details on chromatographic conditions are indicated under each compound. All ^{1}H NMR and ^{13}C NMR spectra were recorded using a Bruker AV-400 or AV-700 spectrometer at 300 K. Chemical shifts are given in parts per million (ppm, δ), referenced to the solvent peak of $CDCl_3$, defined at δ = 7.26 ppm (^{1}H NMR) and δ = 77.16 ppm (^{13}C NMR) or CD_2Cl_2, defined at δ = 5.32 ppm (^{1}H NMR) and δ = 54.00 ppm (^{13}C NMR). Coupling constants are quoted in Hz (*J*). ^{1}H NMR splitting patterns are designated as singlet (s), doublet (d), triplet (t), quartet (q), pentet (p) as they appeared in the spectrum. If the appearance of a signal differs from the expected splitting pattern, the observed pattern is designated as apparent (app). Splitting patterns that could not be interpreted or easily visualized are designated as multiplet (m) or broad (br).

2. Optimization

2.1. Optimization Table

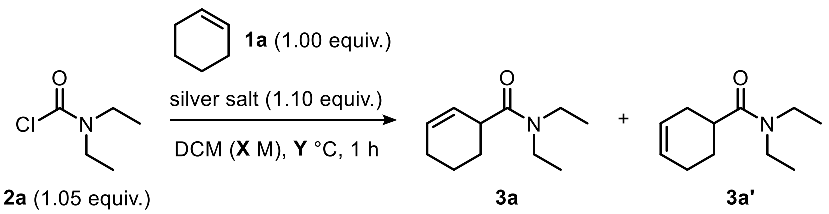

Entry	time	T (°C)	solvent	silver salt (equiv.)	1a/2a/salt eq.	conc. (M)	r.r.	NMR yield (%)
1	10 min	0	DCM	$AgSbF_6$ (1.1)	1.0/1.05/1.1	0.1	14:1	65
2	10 min	0	DCM	$AgSbF_6$ (1.1)	1.0/1.05/1.1	0.1	>20:1	48
3	15 min	0	DCM	$AgSbF_6$ (1.1)	1.0/1.05/1.1	0.1	18:1	66
4	30 min	0	DCM	$AgSbF_6$ (1.1)	1.0/1.05/1.1	0.1	>20:1	38
5	45 min	0	DCM	$AgSbF_6$ (1.1)	1.0/1.05/1.1	0.1	13:1	75
6	1 h	0	DCM	$AgSbF_6$ (1.1)	1.0/1.05/1.1	0.1	>20:1	57
7	1 h	-78	DCM	$AgSbF_6$ (1.1)	1.0/1.05/1.1	0.1	13:1	62
8	1 h	0	DCM	$AgSbF_6$ (1.1)	1.0/1.05/1.1	0.1	>20:1	60
9	1 h	23	DCM	$AgSbF_6$ (1.1)	1.0/1.05/1.1	0.1	>20:1	76
10	1 h	35	DCM	$AgSbF_6$ (1.1)	1.0/1.05/1.1	0.1	12:1	82
11	1 h	23	$CHCl_3$	$AgSbF_6$ (1.1)	1.0/1.05/1.1	0.1	15:1	53
12	1 h	23	HFIP	$AgSbF_6$ (1.1)	1.0/1.05/1.1	0.1	n.d.	n.d.
13	1 h	23	nitromethane	$AgSbF_6$ (1.1)	1.0/1.05/1.1	0.1	2:1	36
14	1 h	23	ACN	$AgSbF_6$ (1.1)	1.0/1.05/1.1	0.1	3:1	3
15	1 h	23	1,2-difluorobenzene	$AgSbF_6$ (1.1)	1.0/1.05/1.1	0.1	15:1	64
16	1 h	23	cyclohexane	$AgSbF_6$ (1.1)	1.0/1.05/1.1	0.1	n.d.	8
17	1 h	23	DCM	AgOTf (1.1)	1.0/1.05/1.1	0.1	>20:1	66
18	1 h	23	DCM	$AgBF_4$ (1.1)	1.0/1.05/1.1	0.1	8:1	8
19	1 h	23	DCM	$AgPF_6$ (1.1)	1.0/1.05/1.1	0.1	n.d.	n.d.
20	1 h	23	DCM	Ag_2CO_3 (1.1)	1.0/1.05/1.1	0.1	1.5:1	0
21	1 h	23	DCM	$AgNO_3$ (1.1)	1.0/1.05/1.1	0.1	n.d.	n.d.
22	1 h	23	DCM	no Ag	1.0/1.05/1.1	0.1	n.d.	n.d.
23	1 h	23	DCM	$AgNTf_2$ (1.1)	1.0/1.05/1.1	0.1	16:1	87
24	1 h	23	DCM	AgOTf (1.1)	1.0/1.05/1.1	0.1	>20:1	79
25	1 h	23	DCM	$AgNTf_2$ (1.1)	1.0/1.05/1.1	0.1	12:1	91
26	1 h	23	DCM	$AgSbF_6$ (1.1)	1.0/1.05/1.1	0.05	11:1	76

**27**	1 h	23	DCM	$AgSbF_6$ (1.1)	1.0/1.05/1.1	0.1	11:1	82
**28**	1 h	23	DCM	$AgSbF_6$ (1.1)	1.0/1.05/1.1	0.5	12:1	80
**29**	1 h	23	DCM	$AgSbF_6$ (1.1)	1.0/1.05/1.1	0.05	13:1	81
**30**	1 h	23	DCM	$AgSbF_6$ (1.1)	1.0/1.05/1.1	0.1	13:1	82
**31**	**1 h**	**23**	**DCM**	**$AgSbF_6$ (1.1)**	1.0/1.05/1.1	**0.2**	**13:1**	**82**
**32**	1 h	23	DCM	$AgSbF_6$ (1.1)	1.0/1.05/1.1	0.5	>20:1	73
**33**	1 h	23	DCM	$AgSbF_6$ (1.1)	1.0/1.05/1.1	0.1	12:1	77
**34**	1 h	23	DCM	$AgSbF_6$ (1.1)	1.0/1.05/1.1	0.1	13:1	75
**35**	1 h	23	DCM	$AgSbF_6$ (1.1)	1.0/1.05/1.1	0.1	13:1	80
**36**	1 h	18	DCM	$AgSbF_6$ (1.1)	1.0/1.05/1.1	0.1	13:1	87
**37**	1 h	25	DCM	$AgSbF_6$ (1.1)	1.0/1.05/1.1	0.1	12:1	72
**38**	1 h	0 to 23	DCM	$AgSbF_6$ (1.1)	1.0/1.05/1.1	0.1	13:1	78
**39**	1 h	0 to 23	DCM	AgOTf (1.1)	1.0/1.05/1.1	0.1	>20:1	53
**40**	1 h	0 to 23	DCM	$AgNTf_2$ (1.1)	1.0/1.05/1.1	0.1	13:1	69
**41**	1 h	0 to 23	DCM	$AgSbF_6$ (1.1)	1.0/1.05/1.1	0.1	13:1	70
**42**	1 h	0 to 23	DCM	AgOTf (1.1)	1.0/1.05/1.1	0.1	>20:1	55
**43**	1 h	0 to 23	DCM	$AgNTf_2$ (1.1)	1.0/1.05/1.1	0.1	12:1	70
**44**	1 h	23	DCM	$AgSbF_6$ (1.1)	1.0/1.05/1.1	0.4	12:1	81
**45**	1 h	23	DCM	AgOTf (1.1)	1.0/1.05/1.1	0.4	>20:1	72
**46**	1 h	23	DCM	$AgNTf_2$ (1.1)	1.0/1.05/1.1	0.4	13:1	82
**47**	1 h	23	DCM	$AgSbF_6$ (1.1)	3.0/1.05/1.1	0.2	13:1	76
**48**	1 h	23	DCM	$AgSbF_6$ (1.1)	1.0/1.05/3.5	0.2	11:1	43

r.r. – regioisomeric ratio; HFIP – hexafluoroisopropanol; ACN – acetonitrile; n.d. – not detected.

Yield determined by NMR analysis of the crude reaction mixture, using mesitylene as an internal standard.

2.2. Triflate Adduct

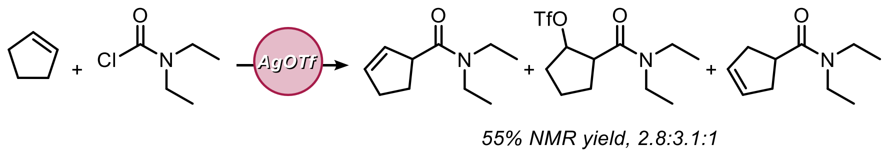

When silver triflate was used instead of silver hexafluoroantimonate, a side-product resulting from triflate addition was observed for some substrates—specifically, cyclopentenes and some linear olefins. Notably, in these cases, the triflate adduct was often found to be the major component of the product mixture. It was confirmed by HSQC and COSY that the triflate adduct formed on the β-position (Figure S1).

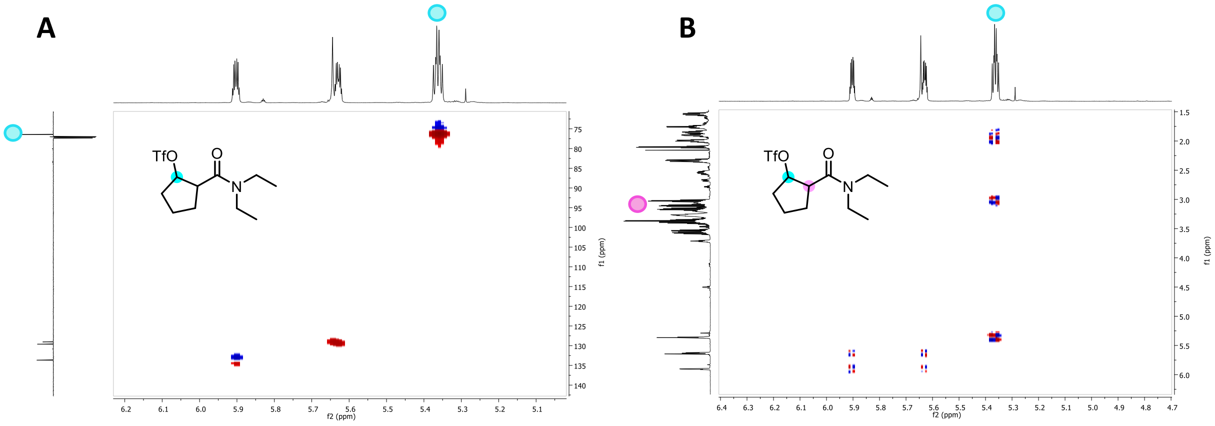

Figure S1. A) HSQC spectrum of crude mixture including triflate adduct; **B)** COSY spectrum of crude mixture including triflate adduct

3. Mechanistic Experiments

3.1. Intramolecular Deprotonation by the Amide

When the reaction was performed using CD_2Cl_2 and monitored, a broad singlet around 14 ppm was observed in the ^{1}H-NMR spectrum before the work up, which was assumed to correspond to the protonated amide (Figure S2A, middle spectrum). The hypothesis of in-situ deprotonation to form the alkene was further supported by an experiment using an additional base, Cs_2CO_3 without observing any change in the reaction outcome. Additionally, the independence of the yield from the concentration of the reaction (see the Optimization Table, Section 2.1), hints at intramolecular deprotonation.

Moreover, the spectrum obtained when *N*,*N*-dimethylcyclohex-2-ene-1-carboxamide was treated with triflic acid provided an additional comparison (Figure S2A, bottom spectrum). Upon comparing the ^{1}H-NMR (Figure S2) and ^{13}C-NMR (Figure S3) spectra of the neutral, isolated *N*,*N*-dimethylcyclohex-2-ene-1-carboxamide (used as a standard), the monitored reaction with silver hexafluoroantimonate, and *N*,*N*-dimethylcyclohex-2-ene-1-carboxamide with triflic acid, a clear downfield shift of several peaks was observed in the latter two cases. In the ^{1}H-NMR spectrum, the α-protons shifted from 3.39 ppm to 3.87 ppm, and the protons of the two methyl groups shifted from 3.04 ppm to 3.38 ppm (Figure S2, B). In the ^{13}C-NMR spectrum, there was also a clear shift of the *N*-dimethyl carbon atoms (Figure S3, B). The most significant change, however, was observed for the carbonyl carbon, which shifted from 174.8 ppm to 178.4 ppm (Figure S3, A)—a characteristic shift for imidate formation.[1]

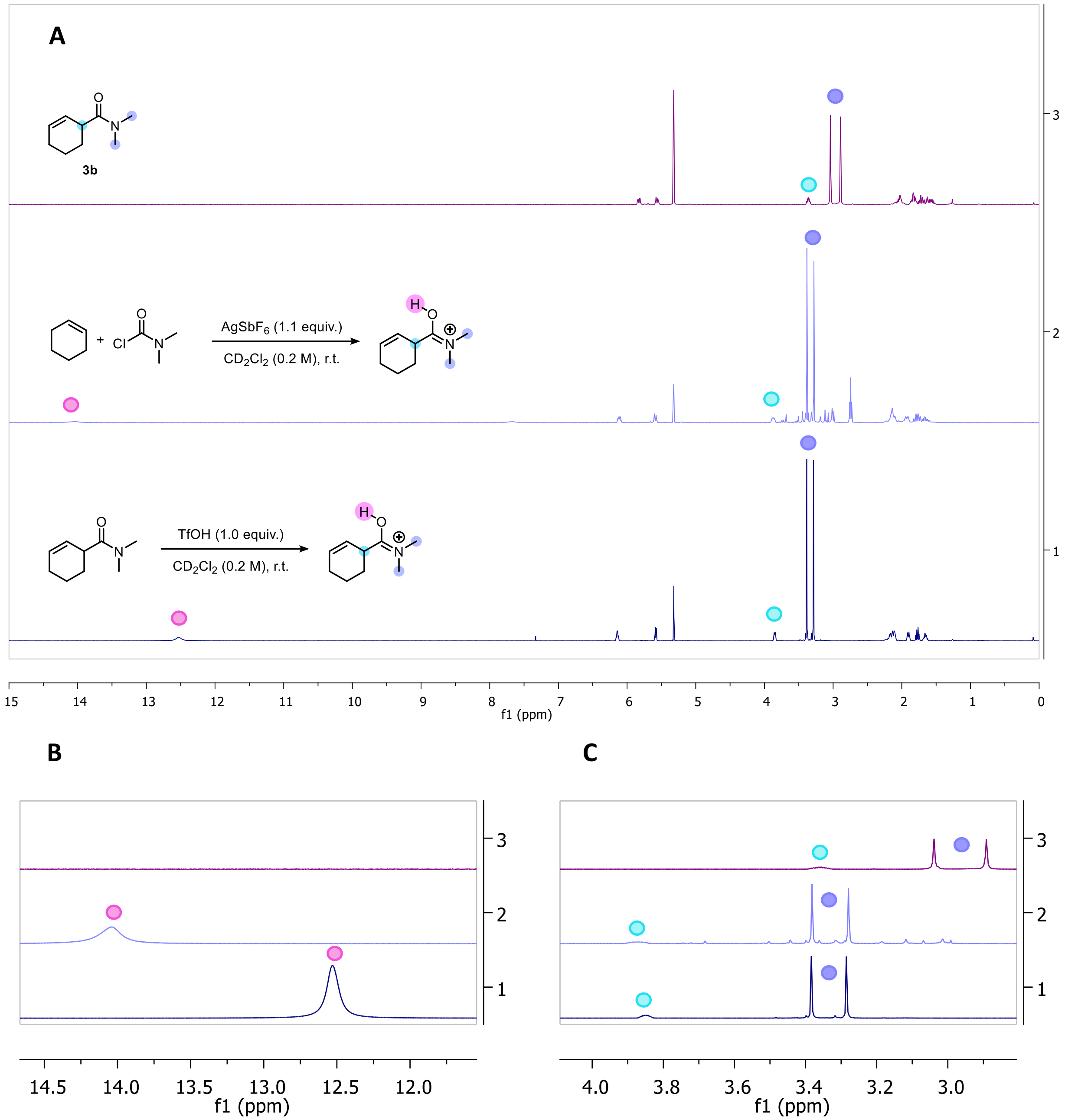

Figure S2. A) Comparison of the ^{1}H-NMR spectra of neutral isolated β,γ-unsaturated product (**3b**) (top), reaction monitoring using standard conditions in CD_2Cl_2 for the formation of **3b** (middle), and protonation of isolated **3b** in CD_2Cl_2 using triflic acid (bottom); **B)** Zoom on the 14.5 to 11.5 ppm range to compare highly deshielded protons (imidate, pink dot); **C)** Zoom on the 4.0 to 2.5 ppm range to compare *N*-methyl protons (violet dot) and α-protons (cyan dot).

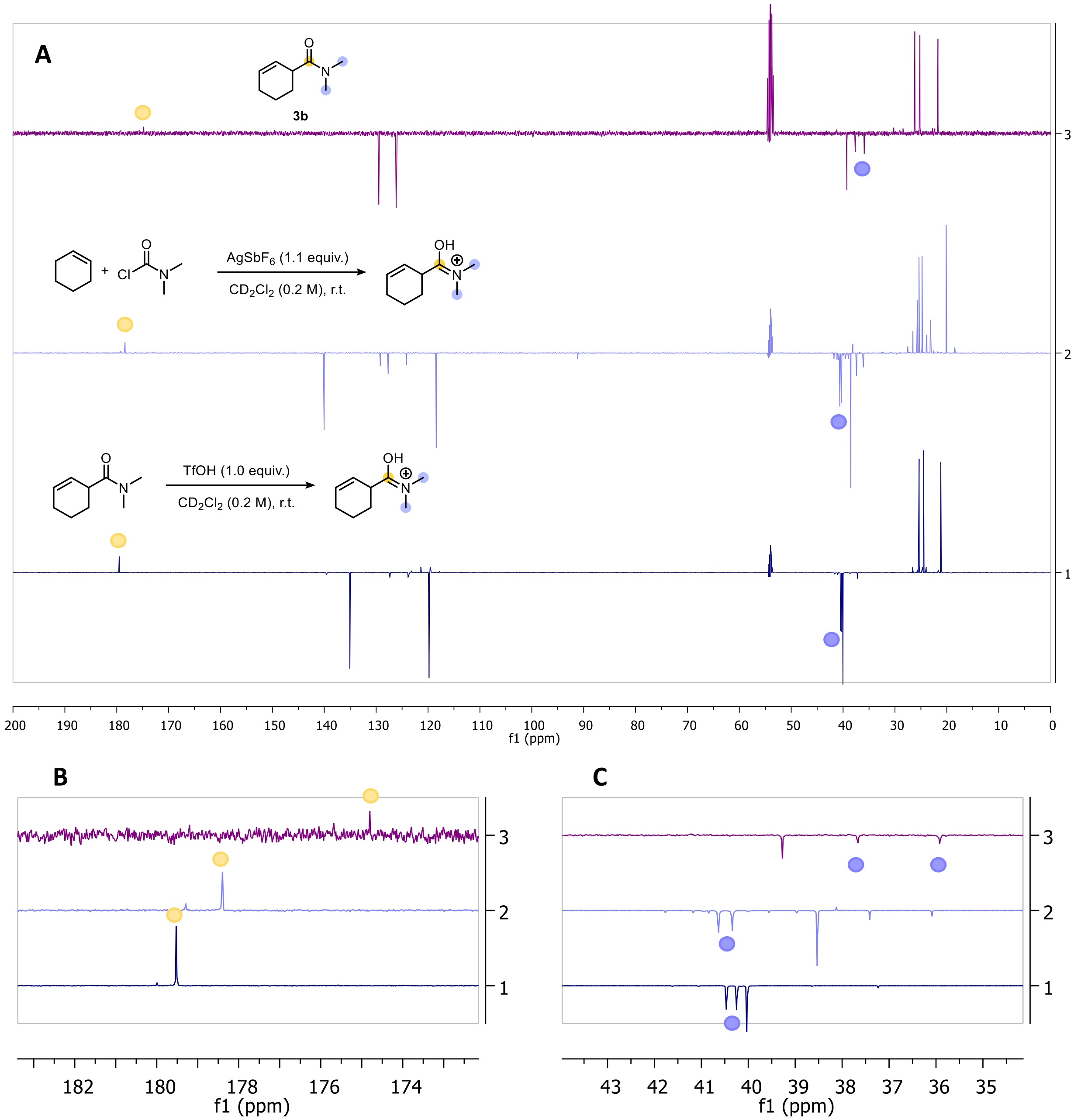

Figure S3. A) Comparison of the ^{13}C-NMR spectra of neutral isolated β,γ-unsaturated product (**3b**) (top), reaction monitoring using standard conditions in CD_2Cl_2 for the formation of **3b** (middle), and protonation of isolated **3b** in CD_2Cl_2 using triflic acid (bottom); **B)** Zoom on the 185 to 170 ppm range to compare highly deshielded carbons (imidate, yellow dot); **C)** Zoom on the 43 to 35 ppm range to compare *N*-methyl carbons (violet dot).

3.2. Evaluating Re-protonation/Elimination Route

Considering the lack of experimental evidence for the hydride shift pathway and the necessity of an explanation for the formation of the minor regioisomer (γ,δ-unsaturated amide, **3a'**), an alternative route was hypothesized. The olefin of the product (**3a**) might be protonated due to the presence of acidic protonated amides in solution (pKa = –0.5). This would generate carbocations located at the β- and γ-positions in similar quantities, opening a pathway to the minor regioisomer (**3a'**). This alternative was eventually discarded after conducting a mechanistic experiment in which a regioisomeric mixture of *N*,*N*-diethylcyclohexenecarboxamides (**3a** and **3a'**) in dichloromethane was treated with 1.0 equivalent of deuterated triflic acid at room temperature. The reaction was monitored by ^{1}H-NMR, and neither deuterium incorporation, nor olefin isomerization were detected. This led to the conclusion that no protonation/deprotonation events are involved.

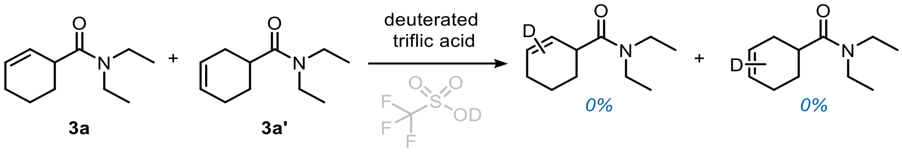

4. Problematic/Failed Substrates

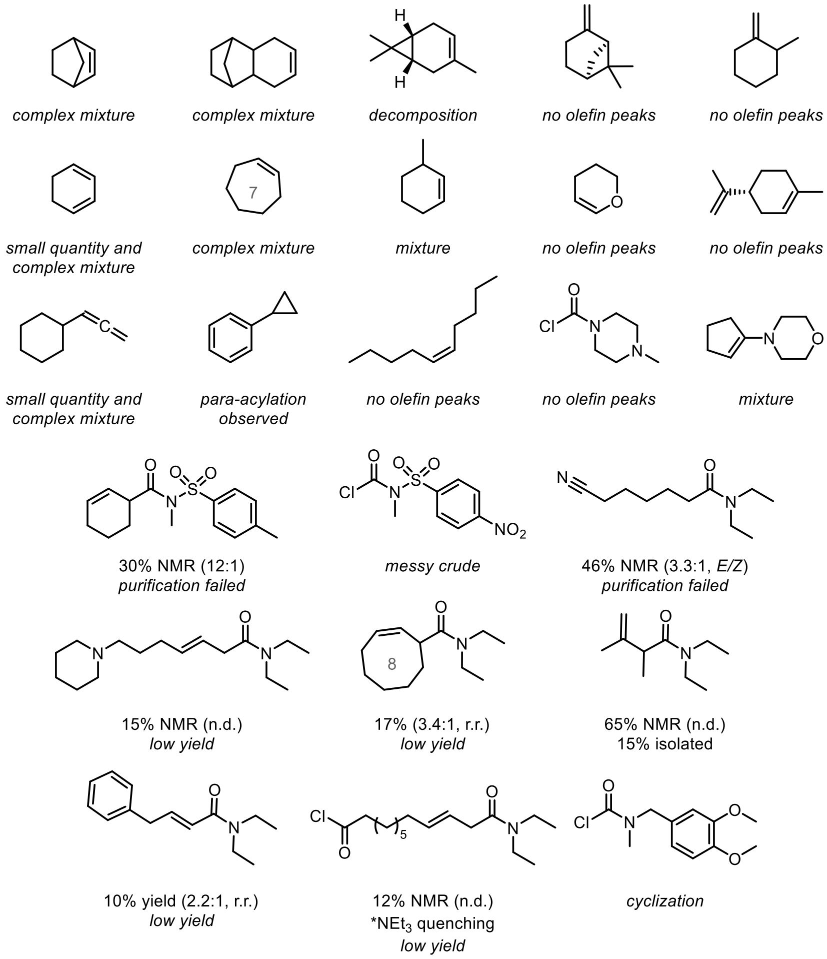

5. β-Fluoro-α,β-Unsaturated Amides Synthesis from Alkynes

When an alkyne was treated with a carbamoyl chloride and a silver salt, the β-fluoro-α,β-unsaturated amide was formed.

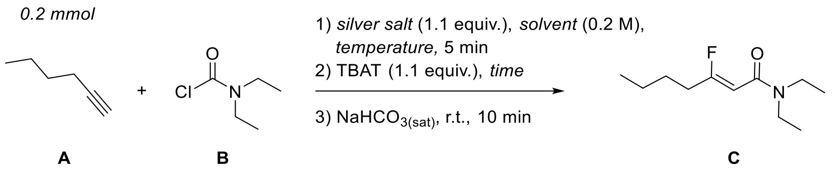

Entry	A (equiv.)	B (equiv.)	silver salt	solvent	T (°C)	time (h)	Quench	NMR yield (%)
**1**	1.0	1.05	$AgSbF_6$	DCM	r.t.	1	$NaHCO_3$	20
**2**	1.0	1.05	$AgSbF_6$	DCM	0	1	$NaHCO_3$	12
**3**	1.0	1.05	$AgSbF_6$	DCM	-78	1	$NaHCO_3$	10
**4**	1.0	1.05	$AgNTf_2$	DCM	0	1	$NaHCO_3$	n.d.
**5**	1.0	1.05	$AgNTf_2$	DCM	-78	1	$NaHCO_3$	n.d.
**6**	1.0	1.05	$AgSbF_6$	DCM	0	2	$NaHCO_3$	10
**7**	1.0	1.05	$AgNTf_2$	DCM	0	2	$NaHCO_3$	n.d.
**8**	1.0	1.05	$AgBF_4$	DCM	0	2	$NaHCO_3$	n.d.
**9**	1.0	1.05	$AgSbF_6$	*o*-DCB	0	2	$NaHCO_3$	15
**10**	1.0	1.05	$AgNTf_2$	*o*-DCB	0	2	$NaHCO_3$	n.d.
**11**	1.0	1.05	$AgSbF_6$	trifluorotoluene	0	2	$NaHCO_3$	15
**12**	1.0	1.05	$AgNTf_2$	trifluorotoluene	0	2	$NaHCO_3$	n.d.
**13**	1.0	1.05	$AgSbF_6$	*o*-DCB	r.t.	3	$NaHCO_3$	12
**14**	1.0	1.05	$AgSbF_6$	*o*-DCB	50	3	$NaHCO_3$	20
**15**	1.0	1.05	$AgSbF_6$	*o*-DCB	100	3	$NaHCO_3$	22
**16**	1.0	1.05	$AgSbF_6$	*o*-DCB	150	o.n.	$NaHCO_3$	6
**17**	1.0	1.05	$AgSbF_6$	*o*-DCB	150	o.n.	$NaHCO_3$	8
**18**	1.0	1.05	$AgSbF_6$	HFIP	150	o.n.	$NaHCO_3$	n.d.
**19**	1.0	1.05	$AgSbF_6$	*o*-DCB	150	2	$NaHCO_3$	26
**20**	1.0	1.05	$AgSbF_6$	*o*-DCB	-10 to 150	1	$NaHCO_3$	21
**21**	1.0	1.05	$AgSbF_6$	*o*-DCB	-10 to 150	1	$NaHCO_3$	10
**22**	4.5	1.05	$AgSbF_6$	*o*-DCB	150	2	$NaHCO_3$	26
**23**	1.0	5.0	$AgSbF_6$	*o*-DCB	150	2	$NaHCO_3$	n.d.
**24**	**1.0**	**1.05**	**$AgSbF_6$ (1.1)**	***o*-DCB**	**150**	**2**	**NEt_3**	**29**

r.r. – regioisomeric ratio; DCM – dichloromethane; *o*-DCB – 1,2-dichlorobenzene; HFIP – hexafluoroisopropanol; n.d. – not detected. Yield determined by NMR analysis of the crude reaction mixture, using mesitylene as an internal standard.

It was observed that higher temperatures and quenching with triethylamine instead of sodium bicarbonate (avoiding the aqueous work up), improved the yield up to a 29%.

6. Chloroformates

The higher basicity of amides, when compared to ketones, seems to favor proton abstraction over oxocarbenium ion formation. This shift in apparent preference prompted us to consider chloroformates, the ester-congeners of acyl chlorides and carbamoyl chlorides, as alternative reagents. Given the fact that the pK_a values of protonated esters (pK_a ca. –6) lie between those of protonated amides (pK_a ca. –0.5) and protonated ketones (pK_a ca. –7), we were interested in further exploring the reactivity of chloroformates in similar contexts..[2] However, when employing $AgSbF_6$, we observed the exclusive formation of the corresponding fluoroformate **C**, while other silver salts led to no reaction or to non-specific decomposition of the starting materials.

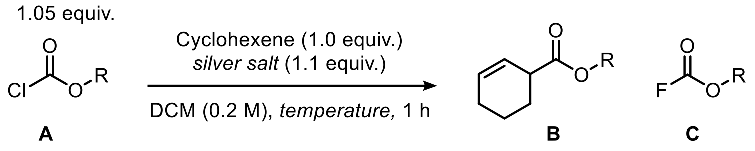

Entry	R	Silver salt	T (°C)	A (%)	B (%)	C (%)
**1**	Isopropenyl	$AgSbF_6$	23	n.d.	n.d.	n.d.
**2**	Phenyl	$AgSbF_6$	23	n.d.	n.d.	59
**3**	Methyl	$AgSbF_6$	23	n.d.	n.d.	n.d.
**4**	Isopropenyl	$AgSbF_6$	-10	n.d.	n.d.	n.d.
**5**	Phenyl	$AgSbF_6$	-10	n.d.	n.d.	71
**6**	Methyl	$AgSbF_6$	-10	n.d.	n.d.	n.d.
**7**	Phenyl	$AgSbF_6$	-78	n.d.	n.d.	96
**8**	Methyl	$AgSbF_6$	-78	n.d.	n.d.	n.d.
**9**	Phenyl	AgOTf	0	100	n.d.	n.d.
**10**	Phenyl	$AgNTf_2$	0	75	n.d.	n.d.
**11**	Phenyl	AgOTf	r.t.	n.d.	n.d.	n.d.
**12**	Phenyl	$AgNTf_2$	r.t.	n.d.	n.d.	n.d.
**13**	Phenyl	AgOTf	40	n.d.	n.d.	n.d.
**14**	Phenyl	$AgNTf_2$	40	n.d.	n.d.	n.d.

7. Experimental Procedures

7.1. Synthesis of Starting Materials

7.1.1. Synthesis of Non-Commercially Available Olefins & Amines

The following starting materials were prepared according to the literature: **7ab,**[3] **7ac,**[3] **1aa,**[4] **1y**.[5]

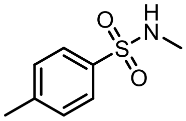

7ac

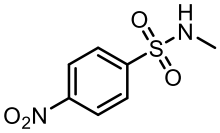

7ad

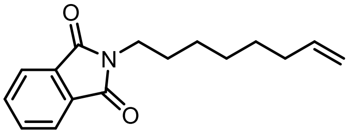

1aa

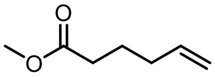

1y

7.1.2. General Procedure A: Synthesis of Carbamoyl Chlorides by Phosgenation

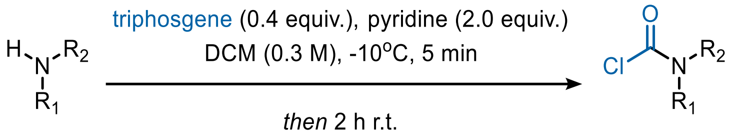

All reactions were run on a 0.5 mmol scale.

A solution of secondary amine (**7**) (1.0 equiv.) and pyridine (2.0 equiv.) in dichloromethane (0.3 M) was cooled to –10°C. Triphosgene ($OC(OCCl_3)_2$, 0.4 equiv.), previously weighed in a closed vial, was added in one portion. After stirring for 5 minutes, the resulting mixture was allowed to warm to room temperature (23 °C) and stirred for 2 h. The reaction was diluted with dichloromethane, quenched by the addition of hydrochloric acid (HCl, 1.0 M, aq.), and the product was extracted using dichloromethane (CH_2Cl_2), the combined organic layers were washed with brine and dried over anhydrous magnesium sulfate ($MgSO_4$). The solvent was removed *in vacuo* and the crude material was analyzed by NMR, adding mesitylene (0.5 equiv.) as an internal standard to calculate the NMR yield (%). The crude product (**2**) was used for the next step without any further purification.

3,4-Dihydroisoquinoline-2(1*H*)-carbonyl chloride (2f)

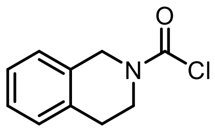

2f
$C_{10}H_{10}ClNO$
MW: 195.65

Synthesized following **General Procedure A** using 1,2,3,4-tetrahydroisoquinoline (62.6 µL, 0.50 mmol, 1.00 equiv.), triphosgene (59.4 mg, 0.20 mmol, 0.40 equiv.), pyridine (80.9 µL, 1.00 mmol, 2.00 equiv.) and dichloromethane (0.30 M, 1.7 mL). The crude material (yellow oil, 92% NMR yield (rotamers A/B 55:45)) was employed in the next step without further purification.

^{1}H NMR (400 MHz, CDCl$_3$): δ 7.29 – 7.22 (m, 2H), 7.22 – 7.17 (m, 1H), 7.17 – 7.11 (m, 1H), 4.85 (s, 1H), 4.77 (s, 1H), 3.93 (t, *J* = 6.0 Hz, 1H), 3.86 (t, *J* = 6.0 Hz, 1H), 3.01 – 2.91 (m, 2H) ppm.

^{13}C{^{1}H} NMR (101 MHz, CDCl$_3$): δ 148.8, 148.7, 137.8, 134.1, 134.0, 132.1, 131.8, 128.8, 128.6, 127.3, 126.9, 126.9, 126.4, 126.3, 50.1, 48.1, 46.6, 44.4, 29.0, 28.6 ppm.

IR (neat) $\tilde{\nu}_{max}$: 3025, 2924, 2852, 1698, 1653, 1602, 1576, 1539, 1458, 1426, 1390, 1369, 1316, 1293, 1224, 1159, 1143, 1091, 1064, 1034, 1021, 771, 748, 706 cm^{-1}.

HRMS – GC (+EI) *m/z*: [M]$^+$ Calcd for $C_{10}H_{10}ClNO^+$ 195.0446; Found 195.0443.

N-Methyl-*N*-phenylcarbamoyl chloride (2h)

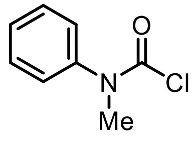

2h
C_8H_8ClNO
MW: 169.61

Synthesized following **General Procedure A** using *N*-methylaniline (54.2 µL, 0.50 mmol, 1.00 equiv.), triphosgene (59.4 mg, 0.20 mmol, 0.40 equiv.), pyridine (80.9 µL, 1.00 mmol, 2.00 equiv.) and dichloromethane (0.30 M, 1.7 mL). The crude material (brown dense oil, 80% NMR yield) was employed in the next step without further purification.

^{1}H NMR (400 MHz, $CDCl_3$): δ 7.52 – 7.34 (m, 3H), 7.32 – 7.18 (m, 2H), 3.38 (s, 3H) ppm.

^{13}C{^{1}H} NMR (101 MHz, $CDCl_3$): δ 155.7, 139.7, 129.8 (2C), 128.7, 127.6 (2C) ppm. *The N-methyl carbon could not be observed.*

IR (neat) $\tilde{\nu}_{max}$: 2924, 2853, 1736, 1596, 1495, 1455, 1416, 1355, 1310, 1289, 1251, 1220, 1173, 1111, 1074, 1046, 1031, 1017, 1001, 855, 771, 696, 686, 666, 559, 524, 474, 418 cm^{-1}.

HRMS – GC (+EI) *m/z*: $[M]^+$ Calcd for $C_8H_8ClNO^+$ 169.0289; Found 169.0285.

***N*-Methyl-*N*-tosylcarbamoyl chloride (2ac)**

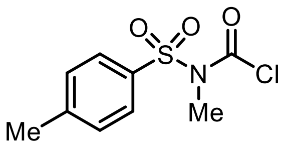

2ac
$C_9H_{10}ClNO_3S$
MW: 247.69

Synthesized following **General Procedure A** using *N*-methyl-*p*-toluenesulfonamide (**7ac**) (92.6 mg, 0.50 mmol, 1.00 equiv.), triphosgene (59.4 mg, 0.20 mmol, 0.40 equiv.), pyridine (80.9 µL, 1.00 mmol, 2.00 equiv.) and dichloromethane (0.30 M, 1.7 mL). The crude material (yellow wax, 67% NMR yield) was employed in the next step without further purification.

^{1}H NMR (400 MHz, $CDCl_3$): δ 7.87 (d, *J* = 8.4 Hz, 2H), 7.36 (d, *J* = 8.0 Hz, 2H), 3.56 (s, 3H), 2.46 (s, 3H) ppm.

^{13}C{^{1}H} NMR (101 MHz, $CDCl_3$): δ 146.2, 134.3, 129.9 (2C), 129.0 (2C), 36.6, 21.9 ppm. *The quaternary carbon in position 4 of the substituted benzene (bound to the methyl group) could not be observed.*

IR (neat) $\tilde{\nu}_{max}$: 3321, 3027, 2921, 2850, 1762, 1744, 1597, 1494, 1456, 1402, 1370, 1327, 1307, 1292, 1249, 1219, 1190, 1174, 1163, 1122, 1092, 1068, 1011, 836, 814, 771, 702, 680, 631, 550 cm^{-1}.

HRMS (ESI$^+$) *m/z*: $[M+Na]^+$ Calcd for $C_9H_{10}ClNO_3SNa^+$; Found 269.9962.

N-Methyl-*N*-nosylcarbamoyl chloride (2ad)

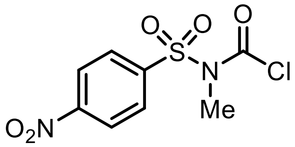

2ad
$C_8H_7ClN_2O_5S$
MW: 278.66

Synthesized following **General Procedure A** using *N*-methyl-p-toluenesulfonamide (**7ad**) (92.6 mg, 0.50 mmol, 1.00 equiv.), triphosgene (59.4 mg, 0.20 mmol, 0.40 equiv.), pyridine (80.9 µL, 1.00 mmol, 2.00 equiv.) and dichloromethane (0.30 M, 1.7 mL). The crude material was submitted a second time to the exact same **General Procedure A** due to low conversion (brown wax, 83% NMR yield after two phosgenations) then was employed in the next step without further purification.

^{1}H NMR (400 MHz, CDCl$_3$): δ 8.40 (d, *J* = 8.9 Hz, 2H), 8.18 (d, *J* = 8.8 Hz, 2H), 3.61 (s, 3H) ppm.

^{13}C{^{1}H} NMR (101 MHz, CDCl$_3$): δ 151.3, 148.3, 142.6, 130.5 (2C), 124.4 (2C), 36.7 ppm.

IR (neat) $\tilde{\nu}_{max}$: 3328, 3029, 2929, 2853, 1773, 1752, 1565, 1499, 1456, 1408, 1373, 1295, 1255, 1221, 1194, 1178, 1166, 1129, 1092, 1068, 1017, 836, 814, 771, 702, 680 cm^{-1}.

HRMS – GC (+EI) *m/z*: [M]$^+$ Calcd for $C_8H_7ClN_2O_5S^+$ 277.9759; Found 277.9562.

7.2. Synthesis of β,γ-Unsaturated Amides from Unactivated Alkenes

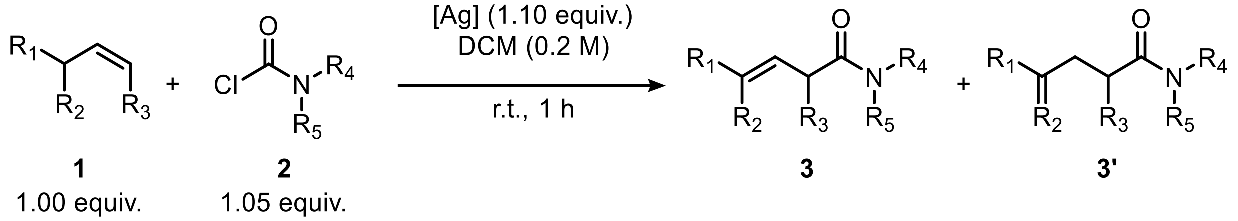

General Procedure B

The olefin (**1**) (1.00 equiv.) and the carbamoyl chloride (**2**) (1.05 equiv.) were dissolved in DCM (0.2 M). Silver trifluoromethanesulfonate (AgOTf, 1.10 equiv.) was subsequently added at ambient temperature (23 °C) and the resulting mixture was allowed to stir at the same temperature for 1 h. Then, $NaHCO_{3(sat)}$ was added and the resulting biphasic solution was stirred for 10 min. The phases were then separated and the aqueous layer was extracted with DCM. The combined organic layers were washed with brine and dried over anhydrous magnesium sulfate. After filtration, the solvent was removed *in vacuo* and the crude material was analyzed by NMR, adding mesitylene (0.50 equiv.) as an internal standard to calculate the NMR yield (%). The crude product was further purified by flash column chromatography on silica gel employing a gradient of ethyl acetate in *n*-heptane, grading from 10% to 80%, after which the final compounds (**3**) were obtained.

General Procedure C

As General Procedure B, using $AgSbF_6$ instead of AgOTf.

General Procedure D

As General Procedure B, using $AgNTf_2$ instead of AgOTf.

***N,N*-Diethylcyclohex-2-ene-1-carboxamide (3a)**

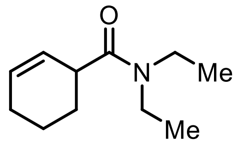

3a
$C_{11}H_{19}NO$
MW: 181.28

Synthesized following **General Procedure B** using cyclohexene (20.3 µL, 0.20 mmol, 1.00 equiv.), diethylcarbamoyl chloride (26.6 µL, 0.21 mmol, 1.05 equiv.), silver trifluoromethanesulfonate (57.7 mg, 0.22 mmol, 1.10 equiv.) and dichloromethane (0.2 M, 1.0 mL). The crude material (72% NMR yield, >20:1 r.r.) was purified by flash column chromatography on silica gel (gradient of EtOAc in *n*-heptane from 10% to 80%) to give the title compound (22.8 mg, 0.13 mmol, 63%, >20:1 r.r.) as a colorless oil.

^{1}H NMR (400 MHz, $CDCl_3$): δ 5.95 – 5.70 (m, 1H), 5.50 (dd, *J* = 10.1, 2.0 Hz, 1H), 3.36 – 3.27 (m, 4H), 3.27 – 3.20 (m, 1H), 2.10 – 2.00 (m, 1H), 2.00 – 1.89 (m, 1H), 1.89 – 1.80 (m, 1H), 1.80 – 1.72 (m, 2H), 1.59 – 1.46 (m, 1H), 1.15 (t, *J* = 7.1 Hz, 3H), 1.05 (t, *J* = 7.1 Hz, 3H) ppm.

^{13}C{^{1}H} NMR (101 MHz, $CDCl_3$): δ 174.1, 129.5, 125.6, 41.9, 40.2, 38.8, 26.1, 24.6, 21.2, 14.9, 13.1 ppm.

IR (neat) $\tilde{\nu}_{max}$: 3270, 3024, 2971, 2933, 2873, 2837, 2206, 2162, 2019, 1741, 1637, 1480, 1428, 1380, 1363, 1282, 1246, 1144, 759 cm^{-1}.

HRMS (ESI^{+}) *m/z:* $[M+H]^+$ Calcd for $C_{11}H_{20}NO^+$ 182.1539; Found 182.1543.

***N,N*-Dimethylcyclohex-2-ene-1-carboxamide (3b)**

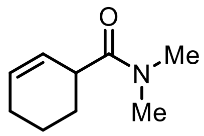

3b
$C_9H_{15}NO$
MW: 153.23

Synthesized following **General Procedure B** using cyclohexene (20.3 µL, 0.20 mmol, 1.00 equiv.), dimethylcarbamoyl chloride (19.3 µL, 0.21 mmol, 1.05 equiv.), silver trifluoromethanesulfonate (57.7 mg, 0.22 mmol, 1.10 equiv.) and dichloromethane (0.2 M, 1.0 mL). The crude material (70% NMR yield, 15:1 r.r.) was purified by flash column chromatography on silica gel (gradient of EtOAc in *n*-heptane from 10% to 80%) to give the title compound (20.3 mg, 0.13 mmol, 66%, 15:1 r.r.) as a yellow oil.

^{1}H NMR (400 MHz, CDCl$_3$): δ 5.87 – 5.79 (m, 1H), 5.59 – 5.53 (m, 1H), 3.38 – 3.30 (m, 1H), 3.04 (s, 3H), 2.92 (s, 3H), 2.11 – 1.96 (m, 2H), 1.89 – 1.70 (m, 3H), 1.60 – 1.49 (m, 1H) ppm.

^{13}C{^{1}H} NMR (101 MHz, CDCl$_3$): δ 174.7, 129.5, 125.1, 38.9, 37.3, 35.8, 25.6, 24.7, 21.2 ppm.

IR (neat) $\tilde{\nu}_{max}$: 3025, 2930, 2172, 2025, 1742, 1641, 1498, 1450, 1395, 1258, 1194, 1147, 1130, 1083, 1048, 758 cm^{-1}.

HRMS (ESI$^+$) *m/z:* [M+H]$^+$ Calcd for $C_9H_{16}NO^+$ 154.1226; Found 154.1230.

Cyclohex-2-en-1-yl(pyrrolidin-1-yl)methanone (3c)

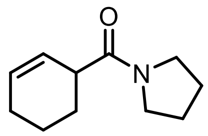

3c
$C_{11}H_{17}NO$
MW: 179.26

Synthesized following **General Procedure B** using cyclohexene (20.3 µL, 0.20 mmol, 1.00 equiv.), 1-pyrrolidinecarbonyl chloride (23.2 µL, 0.21 mmol, 1.05 equiv.), silver trifluoromethanesulfonate (57.7 mg, 0.22 mmol, 1.10 equiv.) and dichloromethane (0.2 M, 1.0 mL). The crude material (60% NMR yield, >20:1 r.r.) was purified by flash column chromatography on silica gel (gradient of EtOAc in *n*-heptane from 10% to 80%) to give the title compound (19.1 mg, 0.11 mmol, 53%, >20:1 r.r.) as a colorless oil.

^{1}H NMR (600 MHz, $CDCl_3$): δ 5.82 – 5.79 (m, 1H), 5.55 – 5.53 (dd, *J* = 10.0, 1.5 Hz, 1H), 3.46 – 3.40 (m, 4H), 3.16 – 3.15 (m, 1H), 2.06 – 2.02 (m, 1H), 1.96 (m, 1H), 1.91 – 1.89 (m, 2H), 1.81 – 1.77 (m, 5H), 1.52 – 1.50 (m, 1H) ppm.

$^{13}C\{^1H\}$ NMR (151 MHz, $CDCl_3$): δ 173.4, 129.6, 124.9, 46.5, 45.9, 40.9, 26.3, 25.3, 24.6, 24.2, 21.1 ppm.

IR (neat) $\tilde{\nu}_{max}$: 3231, 3023, 2934, 2872, 1742, 1635, 1427, 1340, 1304, 1226, 1192, 1038 cm^{-1}.

HRMS (ESI$^+$) *m/z*: $[M+H]^+$ Calcd for $C_{11}H_{17}NO^+$ 180.1383; Found 180.1387.

Cyclohex-2-en-1-yl(piperidin-1-yl)methanone (3d)

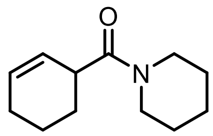

3d
$C_{12}H_{19}NO$
MW: 193.29

Synthesized following **General Procedure B** using cyclohexene (20.3 µL, 0.20 mmol, 1.00 equiv.), 1-piperidinecarbonyl chloride (26.3 µL, 0.21 mmol, 1.05 equiv.), silver trifluoromethanesulfonate (57.7 mg, 0.22 mmol, 1.10 equiv.) and dichloromethane (0.2 M, 1.0 mL). The crude material (60% NMR yield, 14:1 r.r.) was purified by flash column chromatography on silica gel (gradient of EtOAc in *n*-heptane from 10% to 80%) to give the title compound (23.2 mg, 0.12 mmol, 60%, 14:1 r.r.) as a colorless oil.

^{1}H NMR (600 MHz, $CDCl_3$): δ 5.89 – 5.77 (m, 1H), 5.58 (d, *J* = 10.1 Hz, 1H), 3.61 – 3.49 (m, 2H), 3.49 – 3.38 (m, 2H), 3.37 – 3.27 (m, 1H), 2.10 – 1.93 (m, 2H), 1.88 – 1.73 (m, 3H), 1.67 – 1.59 (m, 2H), 1.59 – 1.45 (m, 5H) ppm.

^{13}C{^{1}H} NMR (151 MHz, $CDCl_3$): δ 173.0, 129.4, 125.6, 46.8, 43.0, 38.8, 26.9, 26.0, 25.8, 24.82 (2C), 21.3 ppm.

IR (neat) $\tilde{\nu}_{max}$: 3259, 2934, 2856, 1633, 1434, 1352, 1272, 1249, 1219, 1137, 1124, 1090, 1022, 750 cm^{-1}.

HRMS (ESI$^+$) *m/z*: $[M+H]^+$ Calcd for $C_{12}H_{20}NO^+$ 194.1539; Found 194.1544.

***N,N*-Diisopropylcyclohex-2-ene-1-carboxamide (3e)**

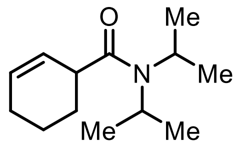

3e
$C_{13}H_{23}NO$
MW: 209.33

Synthesized following **General Procedure C** using cyclohexene (60.8 µL, 0.60 mmol, 1.00 equiv.), *N*,*N*-diispropylcarbamoyl chloride (103 mg, 0.63 mmol, 1.05 equiv.), silver hexafluoroantimonate (57.7 mg, 0.22 mmol, 1.10 equiv.) and dichloromethane (0.2 M, 3.0 mL). The crude material (62% NMR yield, 8.5:1 r.r.) was purified by flash column chromatography on silica gel (gradient of EtOAc in *n*-heptane from 10% to 80%) to give the title compound (77.4 mg, 0.37 mmol, 62%, 9:1 r.r.) as a colorless oil.

^{1}H NMR (400 MHz, $CDCl_3$): δ 5.85 – 5.76 (m, 0.9H), 5.66 (s, 0.2H), 5.53 (dd, *J* = 10.1, 2.1 Hz, 0.9H), 4.00 (m, 1H), 3.45 (s, 0.9H), 3.23 (m, 0.9H), 2.60 (m, 0.1H), 2.37 – 2.23 (m, 0.1H), 2.12 – 1.89 (m, 2H), 1.88 – 1.68 (m, 3H), 1.61 – 1.45 (m, 1H), 1.33 (m, 6H), 1.19 (m, 6H) ppm.

$^{13}C\{^1H\}$ NMR (101 MHz, $CDCl_3$): δ 173.8 (0.1C), 173.7 (0.9C), 129.2 (0.9C), 126.5 (0.1C), 126.2 (0.1C), 125.9 (0.9C), 48.1 (0.2C), 45.8 (1.8C), 45.6 (0.1C), 40.6 (0.9C), 28.5 (0.1C), 26.1 (0.9C), 26.0 (0.1C), 25.1 (0.1C), 24.7 (0.9C), 21.3 (0.9C), 20.8 (2C), 20.7 (2C) ppm.

IR (neat) $\tilde{\nu}_{max}$: 2965, 2931, 2872, 1633, 1437, 1370, 1322, 1301, 1264, 1248, 1212, 1152, 1133, 1044, 898, 755, 647, 606 cm^{-1}.

HRMS (ESI$^+$) *m/z*: $[M+H]^+$ Calcd for $C_{13}H_{24}NO^+$ 210.1852; Found 210.1853.

Cyclohex-2-en-1-yl(3,4-dihydroisoquinolin-2(1*H*)-yl)methanone (3f)

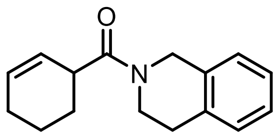

3f
$C_{16}H_{19}NO$
MW: 241.33
isolated isomer

Synthesized following **General Procedure C** using cyclohexene (20.3 µL, 0.20 mmol, 1.00 equiv.), 3,4-dihydroisoquinoline-2(1*H*)-carbonyl chloride (**2f**) (41.1 mg, 0.21 mmol, 1.05 equiv.), silver hexafluoroantimonate (57.7 mg, 0.22 mmol, 1.10 equiv.) and dichloromethane (0.2 M, 1.0 mL). The crude material (61% NMR yield, 5:1 r.r.) was purified by flash column chromatography on silica gel (gradient of EtOAc in *n*-heptane from 10% to 80%) to give the title compounds (27.0 mg, 0.11 mmol, 55%, 5:1 r.r.; 22.5 mg **3f** (46%) and 4.5 mg **3f'** (9%)) as colorless oils.

^{1}H NMR (600 MHz, $CDCl_3$): δ 7.23 – 7.08 (m, 4H), 5.93 – 5.84 (m, 1H), 5.68 – 5.56 (m, 1H), 4.78 – 4.67 (m, 2H), 3.89 – 3.72 (m, 2H), 3.49 – 3.39 (m, 1H), 2.97 – 2.82 (m, 2H), 2.16 – 1.97 (m, 2H), 1.94 – 1.79 (m, 3H), 1.67 – 1.54 (m, 1H) ppm. *Rotameric effects increase the number of peaks.*

^{13}C{^{1}H} NMR (151 MHz, $CDCl_3$): δ 173.9, 173.6, 135.4, 134.8, 134.1, 134.0, 133.8, 133.0, 129.9, 129.8, 129.2, 129.0, 128.7, 128.5, 127.1, 126.9, 126.7, 126.61, 126.57, 126.5, 126.3, 126.1, 125.2, 125.1, 49.0, 47.7, 44.8, 44.7, 43.4, 40.3, 39.5, 39.3, 30.0, 28.9, 28.6, 26.0, 25.8, 24.8, 21.3, 21.2 ppm. *Rotameric effects increase the number of peaks.*

IR (neat) $\tilde{\nu}_{max}$: 3458, 3024, 2922, 2857, 1633, 1435, 1332, 1281, 1253, 1195, 1180, 1113, 1049, 978, 929, 751, 668 cm^{-1}.

HRMS (ESI$^+$) *m/z*: Calcd for $[M+H]^+$ Calcd for $C_{16}H_{20}NO^+$ 242.1539; Found 242.1544.

Cyclohex-3-en-1-yl(3,4-dihydroisoquinolin-2(1*H*)-yl)methanone (3f')

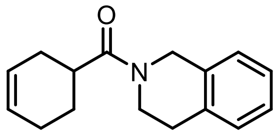

3f'
$C_{16}H_{19}NO$
MW: 241.33
isolated isomer

^{1}H NMR (600 MHz, CDCl$_3$): δ 7.23 – 7.08 (m, 4H), 5.72 (s, 2H), 4.75 (d, *J* = 8.5 Hz, 1H), 4.69 (s, 1H), 3.90 – 3.81 (m, 1H), 3.78 – 3.71 (m, 1H), 2.95 – 2.89 (m, 1H), 2.89 – 2.77 (m, 2H), 2.45 – 2.30 (m, 1H), 2.18 – 2.04 (m, 3H), 1.90 – 1.70 (m, 2H) ppm. *Rotameric effects increase the number of peaks.*

^{13}C{^{1}H} NMR (151 MHz, CDCl$_3$): δ 168.4 (1C), 134.1 (1C), 133.8 (1C), 129.8, 129.2, 128.5, 127.1, 126.9, 126.0, 126.6, 126.5, 126.1, 125.2, 47.7 (1C), 44.7 (1C), 43.4 (1C), 40.3 (1C), 39.3 (1C), 29.9 (1C),2 4.8 (1C) ppm. *Rotameric effects increase the number of peaks.*

IR (neat) $\tilde{\nu}_{max}$: 3022, 2923, 2853, 1637, 1435, 1365, 1292, 1225, 1195, 1180, 1111, 1049, 978, 929, 751, 668, 646 cm^{-1}.

HRMS (ESI$^+$) *m/z*: [M+H]$^+$ Calcd for $C_{16}H_{20}NO^+$ 242.1539; Found 242.1544.

Cyclohex-2-en-1-yl(morpholino)methanone (3g)

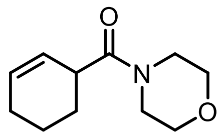

3g
$C_{11}H_{17}NO_2$
MW: 195.26

Synthesized following **General Procedure C** using cyclohexene (81 µL, 0.80 mmol, 1.00 equiv.), 4-morpholinecarbonyl chloride (98 µL, 0.84 mmol, 1.05 equiv.), silver hexafluoroantimonate (305.0 mg, 0.88 mmol, 1.10 equiv.) and dichloromethane (0.2 M, 4.0 mL). The crude material (63% NMR yield, 6:1 r.r.) was purified by flash column chromatography on silica gel (gradient of EtOAc in *n*-heptane from 10% to 80%) to give the title compound (83.3 mg, 0.43 mmol, 53%, 5:1 r.r.) as a colorless oil.

^{1}H NMR (600 MHz, CDCl$_3$): δ 5.89 – 5.81 (m, 0.83H), 5.71 – 5.63 (m, 0.33H), 5.55 (dd, *J* = 10.1, 2.3 Hz, 0.83H), 3.71 – 3.62 (m, 4H), 3.62 – 3.45 (m, 4H), 3.33 – 3.24 (m, 0.83H), 2.72 – 2.62 (m, 0.17H), 2.38 – 2.26 (m, 0.17H), 2.12 – 1.91 (m, 2.17H), 1.90 – 1.66 (m, 2.83H), 1.62 – 1.48 (m, 0.83H) ppm.

^{13}C{^{1}H} NMR (151 MHz, CDCl$_3$): δ 174.5 (0.17C), 173.4 (0.83C), 129.9 (0.83C), 126.6 (0.17C), 125.7 (0.17C), 124.8 (0.83C), 67.1 (1C), 66.9 (1C), 46.3 (0.83C), 46.1 (0.17C), 42.2 (0.83C), 42.1 (0.17C), 38.6 (0.83C), 36.3 (0.17C), 28.1 (0.17C), 25.8 (0.83C), 25.7 (0.17C), 24.9 (0.17C), 24.7 (0.83C), 21.1 (0.83C) ppm.

IR (neat) $\tilde{v}_{max}$: 3457, 3296, 2924, 2857, 1634, 1433, 1328, 1300, 1272, 1229, 1194, 1115, 1068, 1035, 1019, 1000, 957, 898, 875, 850, 824, 802, 771, 703, 667, 629.66, 574, 538, 520, 503, 473 cm^{-1}.

HRMS (ESI$^+$) *m/z*: $[M+H]^+$ Calcd for $C_{11}H_{18}NO_2^+$ 196.1332; Found 196.1328.

N-Methyl-N-phenylcyclohex-2-ene-1-carboxamide (3h)

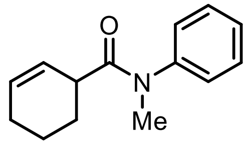

3h
$C_{14}H_{17}NO$
MW: 215.30

Synthesized following **General Procedure C** using cyclohexene (20.3 µL, 0.20 mmol, 1.00 equiv.), *N*-methyl-*N*-phenylcarbamoyl chloride (**2h**) (35.6 mg, 0.21 mmol, 1.05 equiv.), silver hexafluoroantimonate (75.6 mg, 0.22 mmol, 1.10 equiv.) and dichloromethane (0.2 M, 1.0 mL). The crude material (55% NMR yield, 7:1 r.r.) was purified by flash column chromatography on silica gel (gradient of EtOAc in *n*-heptane from 10% to 80%) to give the title compound (14.0 mg, 0.07 mmol, 38%, 10:1 r.r.) as a colorless oil.

^{1}H NMR (600 MHz, $CDCl_3$): δ 7.42 (t, *J* = 7.5 Hz, 2H), 7.33 (t, *J* = 7.4 Hz, 1H), 7.20 (dd, *J* = 5.2, 3.2 Hz, 2H), 5.85 – 5.75 (m, 0.91H), 5.62 – 5.53 (m, 0.18H), 5.50 (d, *J* = 9.7 Hz, 0.91H), 3.26 (s, 3H), 3.16 – 3.03 (m, 0.91H), 2.50 – 2.39 (m, 0.09H), 2.39 – 2.27 (m, 0.09H), 2.12 – 1.98 (m, 1H), 1.95 – 1.84 (m, 1H), 1.84 – 1.72 (m, 2H), 1.71 – 1.59 (m, 0.91H), 1.41 – 1.26 (m, 1H) ppm.

^{13}C{^{1}H} NMR (151 MHz, $CDCl_3$): δ 175.3 (0.91C), 175.1 (0.09C), 144.4 (0.91C), 144.3 (0.09C), 129.9 (0.91C), 129.6 (0.91C) 127.9 (0.91C), 127.5 (0.91C), 127.4 (0.18C), 126.3 (0.18C), 125.9 (0.09C), 125.3 (0.91C), 39.4 (1C), 37.9 (0.91C), 37.4 (0.09C), 28.3 (0.09C), 26.2 (0.91C), 26.0 (0.09C), 24.7 (0.09C), 24.5 (0.91C), 21.0 (0.91C) ppm. *2 Carbon peaks cannot be seen in the aromatic region due to rotameric effects and overlapping.*

IR (neat) $\tilde{\nu}_{max}$: 3025, 2932, 2862, 2836, 1652, 1594, 1494, 1451, 1418, 1377, 1347, 1327, 1307, 1248, 1222, 1158, 1119, 1074, 1035, 992, 927, 898, 795, 773, 701, 679, 570 cm^{-1}.

HRMS – GC (+EI) *m/z*: $[M]^+$ Calcd for $C_{14}H_{17}NO^+$ 215.1310; Found 215.1299.

***N*,*N*-Diethyl-2,3,4,5-tetrahydro-[1,1'-biphenyl]-2-carboxamide (3i)**

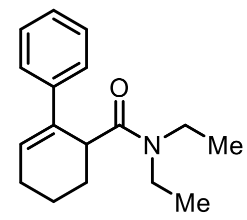

3i
$C_{17}H_{23}NO$
MW: 257.38

Synthesized following **General Procedure C** using 1-phenyl-1-cyclohexene (95.9 µL, 0.60 mmol, 1.00 equiv.), diethylcarbamoyl chloride (79.8 µL, 0.63 mmol, 1.05 equiv.), silver hexafluoroantimonate (85.4 mg, 0.66 mmol, 1.10 equiv.) and dichloromethane (0.2 M, 3 mL). The crude material (55% NMR yield) was purified by flash column chromatography on silica gel (gradient of EtOAc in *n*-heptane from 10% to 80%) to give the title compound (83.1 mg, 0.32 mmol, 54%) as a colorless oil.

^{1}H NMR (400 MHz, $CDCl_3$): δ 7.29 – 7.13 (m, 5H), 6.12 (td, *J* = 4.0, 1.4 Hz, 1H), 3.80 (ddd, *J* = 7.3, 3.8, 1.7 Hz, 1H), 3.39 (dt, *J* = 14.4, 7.3 Hz, 1H), 3.35 – 3.19 (m, 3H), 2.38 – 2.27 (m, 1H), 2.25 – 2.13 (m, 1H), 2.01 – 1.83 (m, 3H), 1.63 (ddd, *J* = 7.9, 6.3, 2.6 Hz, 1H), 1.16 (t, *J* = 7.1 Hz, 3H), 0.92 (t, *J* = 7.1 Hz, 3H) ppm.

^{13}C{^{1}H} NMR (101 MHz, $CDCl_3$): δ 173.2 (1C), 142.6, 136.7, 128.4, 128.2, 126.9, 126.6, 125.5, 126.1, 42.0, 41.1, 40.1, 27.5, 25.6, 19.5, 14.8, 12.7 ppm.

IR (neat) $\tilde{\nu}_{max}$: 2971, 2931, 2870, 1666, 1624, 1479, 1444, 1429, 1379, 1361, 1345, 1313, 1286, 1248, 1217, 1134, 1096, 1072, 1031, 1000, 980, 945, 907, 877, 836, 806, 752, 697, 665, 637 cm^{-1}.

HRMS (ESI^+) *m/z*: $[M+H]^+$ Calcd for $C_{17}H_{24}NO^+$ 258.1852; Found 258.1851.

_N,N_-2-Trimethylcyclohex-2-ene-1-carboxamide (3j)

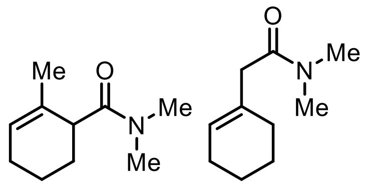

major **3j** minor

$C_{10}H_{17}NO$

MW: 167.25

Synthesized following **General Procedure C** using 1-methyl-1-cyclohexene (60.6 µL, 0.50 mmol, 1.00 equiv.), dimethylcarbamoyl chloride (92.1 µL, 1.00 mmol, 2.00 equiv.), silver hexafluoroantimonate (361.0 mg, 1.05 mmol, 2.1 equiv.) and dichloromethane (0.2 M, 2.5 mL). The crude material (80% NMR yield, 4.7:1) was purified by flash column chromatography on silica gel (gradient of EtOAc in *n*-heptane from 10% to 80%) to give the title compound (83.1 mg, 0.32 mmol, 59%, 8:1 r.r. as a colorless oil.

^{1}H NMR (600 MHz, $CDCl_3$): δ 5.57 (m, 0.89H), 5.41 (m, 0.11H), 3.25 (m, 0.89H), 3.06 (s, 2.67H), 2.99 – 2.89 (m, 3.33H), 2.17 – 1.87 (m, 2.33H), 1.86 – 1.65 (m, 2.67H), 1.63 – 1.53 (m, 3.11H), 1.44 (m, 1H) ppm.

^{13}C{^{1}H} NMR (151 MHz, $CDCl_3$): δ 174.6 (0.89C), 171.3 (0.11C), 131.9 (0.11C), 131.7 (0.89C), 125.0 (0.89C), 124.0 (0.11C) 43.4 (0.11C), 42.9 (0.89C), 37.7 (0.11C), 37.6 (0.89C), 35.9 (0.89C), 35.5 (0.11C), 28.6 (0.11C), 26.7 (0.89C), 25.3 (0.11C), 25.0 (0.89C), 22.8 (0.11C), 22.3 (0.89C), 22.2 (0.11C), 20.2 (0.89C) ppm.

IR (neat) $\tilde{\nu}_{max}$: 2929, 2859, 2836, 1631, 1493, 1446, 1392, 1283, 1256, 1218, 1130, 1094, 1055, 1032, 988, 944, 910, 898, 888, 802, 731, 699, 647, 613, 572, 532, 476, 457, 435 cm^{-1}.

HRMS (ESI$^+$) *m/z*: $[M+H]^+$ Calcd for $C_{10}H_{18}NO^+$ 168.1383; Found 168.1378.

***N*,*N*-Diethylcyclopent-2-ene-1-carboxamide (3k)**

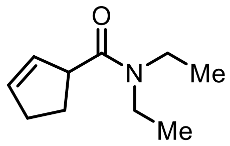

3k
$C_{10}H_{17}NO$
MW: 167.25
isolated isomer

Synthesized following **General Procedure C** using cyclopentene (53.0 µL, 0.60 mmol, 1.00 equiv.), diethylcarbamoyl chloride (79.8 µL, 0.63 mmol, 1.05 equiv.), silver hexafluoroantimonate (227.0 mg, 0.66 mmol, 1.10 equiv.) and dichloromethane (0.2 M, 3.0 mL). The crude material (69% NMR yield, 1.8:1 r.r.) was purified by flash column chromatography on silica gel (gradient of EtOAc in *n*-heptane from 10% to 80%) to give the title compounds (47.5 mg, 0.28 mmol, 47%, 1.9:1 r.r.; 31.1 mg **3k** (31%) and 16.4 mg **3k'** (16%)) as colorless oils.

^{1}H NMR (400 MHz, $CDCl_3$): δ 5.90 (m, 1H), 5.65 – 5.60 (m, 1H), 3.75 – 3.68 (m, 1H), 3.45 – 3.32 (m, 4H), 2.58 – 2.48 (m, 1H), 2.40 – 2.29 (m, 1H), 2.14 – 2.06 (m, 2H), 1.19 (t, *J* = 7.1 Hz, 3H), 1.10 (t, J = 7.1 Hz, 3H) ppm.

^{13}C{^{1}H} NMR (101 MHz, $CDCl_3$): δ 174.1 (1C), 133.7 (1C), 129.6 (1C), 48.3 (1C), 41.9 (1C), 40.2 (1C), 32.6 (1C), 27.8 (1C), 14.8 (1C), 13.1 (1C) ppm.

IR (neat) $\tilde{\nu}_{max}$: 3258, 2973, 2935, 1715, 1621, 1429, 1381, 1363, 1254, 1220, 1140, 1083, 1018, 753 cm^{-1}.

HRMS (ESI$^+$) *m/z*: $[M+H]^+$ Calcd for $C_{10}H_{18}NO^+$ 168.1383; Found 168.1386.

N,N-Diethylcyclopent-3-ene-1-carboxamide (3k')

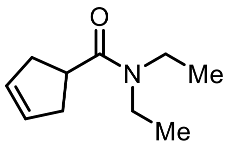

3k'
$C_{10}H_{17}NO$
MW: 167.25
isolated isomer

^{1}H NMR (400 MHz, $CDCl_3$): δ 5.65 (s, 2H), 3.42 – 3.32 (m, 4H), 3.31 – 3.22 (m, 1H), 2.72 – 2.63 (m, 2H), 2.60 – 2.51 (m, 2H), 1.18 (t, J = 7.1 Hz, 3H), 1.11 (t, J = 7.1 Hz, 3H) ppm.

^{13}C{^{1}H} NMR (101 MHz, $CDCl_3$): δ 175.1, 129.0 (2C), 42.0, 40.4, 39.2, 37.4 (2C), 14.9, 13.2 ppm.

IR (neat) $\tilde{\nu}_{max}$: 3258, 2973, 2935, 1715, 1621, 1429, 1381, 1363, 1254, 1220, 1140, 1083, 1018, 753 cm^{-1}.

HRMS (ESI$^+$) m/z: $[M+H]^+$ Calcd for $C_{10}H_{18}NO^+$ 168.1383; Found 168.1386.

_N_,_N_-Dimethylcyclopent-2-ene-1-carboxamide (3l)

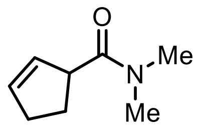

3l

$C_8H_{13}NO$

MW: 139.20

isolated isomer

Synthesized following **General Procedure C** using cyclopentene (70.7 µL, 0.80 mmol, 1.00 equiv.), diethylcarbamoyl chloride (77.3 µL, 0.84 mmol, 1.05 equiv.), silver hexafluoroantimonate (302.0 mg, 0.88 mmol, 1.10 equiv.) and dichloromethane (0.2 M, 4.0 mL). The crude material (52% NMR yield, 1.5:1 r.r.) was purified by flash column chromatography on silica gel (gradient of EtOAc in *n*-heptane from 10% to 80%) to give the title compounds (30.7 mg, 0.22 mmol, 43%, 1.8:1 r.r.; 19.7 mg **3l** (28%) and 11.0 mg **3l'** (15%)) as colorless oils.

^{1}H NMR (400 MHz, $CDCl_3$): δ 5.92 – 5.87 (m, 1H), 5.68 – 5.63 (m, 1H), 3.83 – 3.72 (m, 1H), 3.08 (s, 3H), 2.94 (s, 3H), 2.57 – 2.45 (m, 1H), 2.40 – 2.28 (m, 1H), 2.17 – 2.07 (m, 2H) ppm.

^{13}C{^{1}H} NMR (101 MHz, $CDCl_3$): δ 174.7, 133.6, 129.0, 48.5, 37.4, 35.8, 32.5, 27.2 ppm.

IR (neat) $\tilde{\nu}_{max}$: 3421, 2929, 2863, 1750, 1712, 1608, 1502, 1438, 1400, 1260, 1235, 1162, 1114, 1055, 992, 921, 876, 848, 821, 771, 735, 672, 628, 574, 538, 477, 424 cm^{-1}.

HRMS (ESI$^+$) *m/z*: $[M+Na]^+$ Calcd for $C_8H_{13}NONa^+$ 162.0889; Found 162.0889.

N,*N*-Diethylcyclopent-3-ene-1-carboxamide (3l')

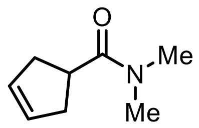

3l'
$C_8H_{13}NO$
MW: 139.20
isolated isomer

^{1}H NMR (400 MHz, $CDCl_3$): δ 5.68 – 5.61 (s, 2H), 3.32 (tt, *J* = 9.4, 6.8 Hz, 1H), 3.05 (s, 3H), 2.96 (s, 3H), 2.73 – 2.63 (m, 2H), 2.62 – 2.52 (m, 2H) ppm.

^{13}C{^{1}H} NMR (101 MHz, $CDCl_3$): δ 175.7, 129.0 (2C), 39.4, 37.4, 36.8 (2C), 35.9 ppm.

IR (neat) $\tilde{\nu}_{max}$: 2924, 2851, 1634, 1495, 1444, 1395, 1343, 1320, 1260, 1180, 1140, 1057, 947, 691, 664, 572 cm^{-1}.

HRMS (ESI$^+$) *m/z*: $[M+H]^+$ Calcd for $C_8H_{14}NO^+$ 140.1070; Found 140.1070.

N,N-Diisopropylcyclopent-2-ene-1-carboxamide (3m)

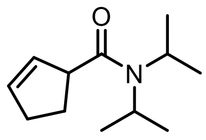

3m
$C_{12}H_{21}NO$
MW: 195.31
isolated isomer

Synthesized following **General Procedure C** using cyclopentene (88.4 µL, 1.00 mmol, 1.00 equiv.), *N,N*-diisopropylcarbamoyl chloride (172.0 mg, 1.05 mmol, 1.05 equiv.), silver hexafluoroantimonate (378.0 mg, 1.10 mmol, 1.10 equiv.) and dichloromethane (0.2 M, 5.0 mL). The crude material (49% NMR yield, 1.3:1 r.r.) was purified by flash column chromatography on silica gel (gradient of EtOAc in *n*-heptane from 10% to 80%) to give the title compounds (68.3 mg, 0.35 mmol, 35%, 1.1:1 r.r.; 35.8 mg **3m** (18%) and 32.5 mg **3m'** (17%)) as colorless oils.

^{1}H NMR (400 MHz, $CDCl_3$): δ 5.91 – 5.87 (m, 1H), 5.69 – 5.63 (m, 1H), 4.15 (dt, *J* = 13.7, 6.7 Hz, 1H), 3.75 – 3.66 (m, 1H), 3.46 (s, 1H), 2.58 – 2.46 (m, 1H), 2.41 – 2.29 (m, 1H), 2.10 (m, 2H), 1.37 (m, 6H), 1.30 – 1.15 (m, 6H) ppm.

$^{13}C\{^1H\}$ NMR (101 MHz, $CDCl_3$): δ 172.0, 133.2, 129.8, 52.4, 50.2, 45.8, 32.6, 27.6, 20.9 (2C), 20.8 (2C) ppm.

IR (neat) $\tilde{\nu}_{max}$: 2961, 2923, 2851, 1730, 1719, 1710, 1640, 1629, 1610, 1591, 1465, 1441, 1369, 1338, 1304, 1273, 1212, 1155, 1133, 1080, 1043, 968, 947, 772, 682, 528, 427, 405 cm^{-1}.

HRMS – (ESI$^+$) *m/z*: $[M+H]^+$ Calcd for $C_{12}H_{22}NO^+$ 196.1696; Found 196.1695.

N,N-Diisopropylcyclopent-3-ene-1-carboxamide (3m')

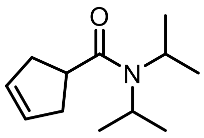

3m'
$C_{12}H_{21}NO$
MW: 195.31
isolated isomer

^{1}H NMR (400 MHz, $CDCl_3$): δ 5.68 – 5.62 (m, 2H), 4.09 (dd, J = 12.8, 6.2 Hz, 1H), 3.50 (s, 1H), 3.25 (tt, J = 9.5, 6.9 Hz, 1H), 2.76 – 2.65 (m, 2H), 2.58 – 2.48 (m, 2H), 1.38 (d, J = 6.8 Hz, 6H), 1.22 (d, J = 6.7 Hz, 6H) ppm.

^{13}C{^{1}H} NMR (101 MHz, $CDCl_3$): δ 154.0, 129.0 (2C), 45.78 (2C), 41.1, 37.1 (2C), 20.9 (4C) ppm.

IR (neat) $\tilde{\nu}_{max}$: 2963, 2925, 2871, 2851, 1719, 1632, 1436, 1369, 1337, 1317, 1303, 1271, 1211, 1151, 1132, 1080, 1043, 968, 947, 908, 897, 871, 833, 801, 771, 722, 688, 621, 592, 525, 482, 454, 423, 408 cm^{-1}.

HRMS – (ESI$^+$) m/z: [M+H]$^+$ Calcd for $C_{12}H_{22}NO^+$ 196.1695; Found 196.1696.

Cyclopent-2-en-1-yl(piperidin-1-yl)methanone (3n)

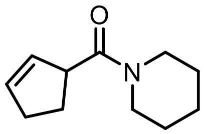

3n
$C_{11}H_{17}NO$
MW: 179.26
isolated isomer

Synthesized following **General Procedure C** using cyclopentene (53.0 µL, 0.60 mmol, 1.00 equiv.), diethylcarbamoyl chloride (78.8 µL, 0.63 mmol, 1.05 equiv.), silver hexafluoroantimonate (229.0 mg, 0.66 mmol, 1.10 equiv.) and dichloromethane (0.2 M, 3.0 mL). The crude material (62% NMR yield, 1.1:1 r.r.) was purified by flash column chromatography on silica gel (gradient of EtOAc in *n*-heptane from 10% to 80%) to give the title compounds (67.8 mg, 0.38 mmol, 63%, 1.1:1 r.r.; 35.5 mg **3n** (33%) and 32.3 mg **3n'** (30%)) as colorless oils.

^{1}H NMR (400 MHz, CDCl$_3$): δ 5.88 (dd, *J* = 5.6, 2.3 Hz, 1H), 5.65 (dt, *J* = 7.7, 2.1 Hz, 1H), 3.74 (td, *J* = 6.7, 4.7 Hz, 1H), 3.61 – 3.43 (m, 4H), 2.56 – 2.45 (m, 1H), 2.40 – 2.28 (m, 1H), 2.19 – 2.03 (m, 2H), 1.68 – 1.61 (m, 2H), 1.61 – 1.47 (m, 4H) ppm.

^{13}C{^{1}H} NMR (101 MHz, CDCl$_3$): δ 172.9, 133.4, 129.4, 48.4, 46.7, 43.0, 32.5, 27.4, 26.8, 25.7, 24.8 ppm.

IR (neat) $\tilde{\nu}_{max}$: 3402, 2935, 2856, 1712, 1605, 1443, 1369, 1352, 1282, 1255, 1228, 1175, 1148, 1126, 1025, 1003, 985, 955, 896, 870, 853, 770, 735, 701, 667, 629, 599, 568, 529, 479, 410 cm^{-1}.

HRMS (ESI$^+$) *m/z*: [M+H]$^+$ Calcd for $C_{11}H_{18}NO^+$ 180.1383; Found 180.1378.

Cyclopent-3-en-1-yl(piperidin-1-yl)methanone (3n')

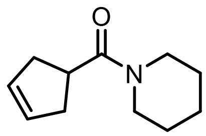

3n'
$C_{11}H_{17}NO$
MW: 179.26
isolated isomer

^{1}H NMR (400 MHz, CDCl$_3$): δ 5.64 (s, 2H), 3.61 – 3.52 (m, 2H), 3.49 – 3.41 (m, 2H), 3.29 (tt, J = 9.5, 6.7 Hz, 1H), 2.76 – 2.64 (m, 2H), 2.61 – 2.49 (m, 2H), 1.69 – 1.60 (m, 2H), 1.59 – 1.49 (m, 4H) ppm.

^{13}C{^{1}H} NMR (101 MHz, CDCl$_3$): δ 173.8, 129.0 (2C), 46.8, 43.2, 39.3, 36.8 (2C), 26.8, 25.7, 24.8 ppm.

IR (neat) $\tilde{\nu}_{max}$: 3389, 2934, 2855, 1713, 1615, 1441, 1368, 1352, 1248, 1224, 1174, 1138, 1124, 1019, 952, 906, 853, 841, 801, 770, 700, 667, 646, 631, 604, 538, 473, 440, 425, 418 cm^{-1}.

HRMS (ESI$^+$) *m/z*: $[M+H]^+$ Calcd for $C_{11}H_{18}NO$ 180.1383; Found 180.1378.

Cyclopent-2-en-1-yl(pyrrolidin-1-yl)methanone (3o)

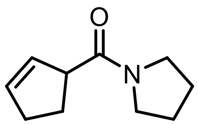

3o
$C_{10}H_{15}NO$
MW: 165.24
isolated isomer

Synthesized following **General Procedure C** using cyclopentene (53.0 µL, 0.60 mmol, 1.00 equiv.), diethylcarbamoyl chloride (69.6 µL, 0.63 mmol, 1.05 equiv.), silver hexafluoroantimonate (229.0 mg, 0.66 mmol, 1.10 equiv.) and dichloromethane (0.2 M, 3.0 mL). The crude material (68% NMR yield, 2:1 r.r.) was purified by flash column chromatography on silica gel (gradient of EtOAc in *n*-heptane from 10% to 80%) to give the title compounds (49.7 mg, 0.30 mmol, 50%, 1.6:1 r.r.; 30.6 mg **3o** (31%) and 19.1 mg **3o'** (19%)) as colorless oils.

^{1}H NMR (400 MHz, CDCl$_3$): δ 5.90 (dd, *J* = 5.6, 2.3 Hz, 1H), 5.69 – 5.62 (m, 1H), 3.67 – 3.59 (m, 1H), 3.50 (t, *J* = 6.8 Hz, 2H), 3.45 (t, *J* = 6.9 Hz, 2H), 2.57 – 2.46 (m, 1H), 2.39 – 2.27 (m, 1H), 2.19 – 2.04 (m, 2H), 1.99 – 1.90 (m, 2H), 1.87 – 1.79 (m, 2H) ppm.

^{13}C{^{1}H} NMR (101 MHz, CDCl$_3$): δ 173.3, 133.8, 128.9, 50.2, 46.6, 46.0, 32.6, 26.9, 26.3, 24.4 ppm.

IR (neat) $\tilde{\nu}_{max}$: 3389, 2955, 2879, 1713, 1624, 1604, 1540, 1447, 1342, 1269, 1228, 1171, 1117, 1045, 990, 872, 771, 666 cm^{-1}.

HRMS (ESI$^+$) *m/z*: [M+H]$^+$ Calcd for $C_{10}H_{16}NO^+$ 166.1226; Found 166.1221.

Cyclopent-3-en-1-yl(pyrrolidin-1-yl)methanone (3o')

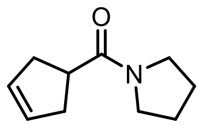

3o'
$C_{10}H_{15}NO$
MW: 165.24
isolated isomer

^{1}H NMR (400 MHz, CDCl$_3$): δ 5.65 (s, 2H), 3.47 (t, *J* = 6.8 Hz, 4H), 3.24 – 3.14 (tt, *J* = 9.4, 7.0 Hz, 1H), 2.74 – 2.64 (m, 2H), 2.63 – 2.53 (m, 2H), 2.00 – 1.91 (m, 2H), 1.89 – 1.80 (m, 2H) ppm.

^{13}C{^{1}H} NMR (101 MHz, CDCl$_3$): δ 174.5, 129.0 (2C), 46.7, 46.1, 41.2, 36.5 (2C), 26.3, 24.5 ppm.

IR (neat) $\tilde{\nu}_{max}$: 3322, 2924, 2876, 1715, 1613, 1549, 1441, 1342, 1296, 1254, 1226, 1171, 1113, 1042, 990, 916, 890, 840, 771, 699, 669, 647, 575, 536, 511, 426, 417 cm^{-1}.

HRMS (ESI$^+$) *m/z*: [M+H]$^+$ Calcd for $C_{10}H_{16}NO^+$ 166.1226; Found 166.1222.

***N,N*-Diethyl-2-methylcyclopent-2-ene-1-carboxamide (3p)**

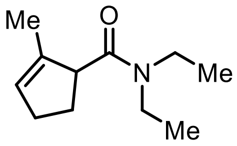

3p
$C_{11}H_{19}NO$
MW: 181.28

Synthesized following **General Procedure C** using 1-methylcyclopentene (84.2 µL, 0.80 mmol, 1.00 equiv.), diethylcarbamoyl chloride (106.0 µL, 0.84 mmol, 1.05 equiv.), silver hexafluoroantimonate (302.0 mg, 0.88 mmol, 1.10 equiv.) and dichloromethane (0.2 M, 4.0 mL). The crude material (57% NMR yield) was purified by flash column chromatography on silica gel (gradient of EtOAc in *n*-heptane from 10% to 80%) to give the title compound (72.2 mg, 0.40 mmol, 50%, >20:1) as a colorless oil.

^{1}H NMR (400 MHz, $CDCl_3$): δ 5.56 – 5.49 (m, 1H), 3.65 – 3.56 (m, 1H), 3.51 – 3.28 (m, 4H), 2.54 – 2.41 (m, 1H), 2.35 – 2.23 (m, 1H), 2.22 – 2.10 (m, 1H), 2.08 – 1.96 (m, 1H), 1.69 (s, 3H), 1.20 (t, *J* = 7.1 Hz, 3H), 1.12 (t, *J* = 7.1 Hz, 3H) ppm.

$^{13}C\{^1H\}$ NMR (101 MHz, $CDCl_3$): δ 174.4, 139.0, 128.3, 50.7, 42.2, 40.6, 32.0, 29.6, 15.4, 15.1, 13.3 ppm.

IR (neat) $\tilde{\nu}_{max}$: 2969, 2934, 2873, 2853, 1698, 1633, 1481, 1444, 1428, 1379, 1362, 1322, 1250, 1219, 1172, 1134, 1096, 1080, 1026, 999, 943, 843, 814, 773, 619, 441 cm^{-1}.

HRMS (ESI$^+$) *m/z:* $[M+H]^+$ Calcd for $C_{11}H_{20}NO$ 182.1539; Found 182.1539.

(*E*)-*N*,*N*-Diethylcyclododec-2-ene-1-carboxamide (3q)

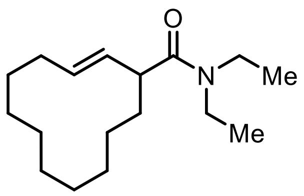

3q
$C_{17}H_{31}NO$
MW: 265.44

Small scale: Synthesized following **General Procedure C** using cyclododecene (*cis*/*trans*-mixture) (154.0 µL, 0.80 mmol, 1.00 equiv.), diethylcarbamoyl chloride (106.0 µL, 0.84 mmol, 1.05 equiv.), silver hexafluoroantimonate (302.0 mg, 0.88 mmol, 1.10 equiv.) and dichloromethane (0.2 M, 4.0 mL). The crude material (53% NMR yield) was purified by flash column chromatography on silica gel (gradient of EtOAc in *n*-heptane from 10% to 80%) to give the title compound (107.0 mg, 0.40 mmol, 50%) as a colorless oil.

Large scale: In a flame-dried 100 mL Schlenk round bottom flask under $Ar_{(g)}$ atmosphere, silver hexafluoroantimonate (2.27 g, 6.61 mmol, 1.10 equiv.) was dissolved in dichloromethane (0.2 M, 30.0 mL). With vigorous stirring, diethylcarbamoyl chloride (800 µL, 6.31 mmol, 1.05 equiv.) was carefully added and, immediately after, cyclododecene (*cis*/*trans*- mixture) (1.16 mL, 1.00 g, 6.01 mmol, 1.00 equiv.) was added dropwise. The mixture was left stirring for 16 h. 30 mL of a saturated solution of $NaHCO_{3(aq)}$ were added and the resulting mixture was left to stir for 10 min. The product was extracted using DCM (3 × 30 mL), the combined organic layers were washed with brine (1 × 30 mL) and dried over anhydrous magnesium sulfate. After filtration, the solvent was removed *in vacuo* and the crude material (65% NMR yield) was purified by flash column chromatography on silica gel (gradient of EtOAc in *n*-heptane from 10% to 80%) to give the title compound (1.07 g, 4.03 mmol, 67%) as a colorless oil.

^{1}H NMR (700 MHz, $CDCl_3$): δ 5.54 – 5.37 (m, 2H), 3.43 – 3.18 (m, 4H), 3.10 – 2.96 (m, 1H), 2.23 – 2.10 (m, 1H), 1.98 – 1.84 (m, 1H), 1.66 – 1.51 (m, 3H), 1.51 – 1.36 (m, 2H), 1.36 – 1.18 (m, 11H), 1.14 (t, *J* = 7.1 Hz, 3H), 0.99 (t, *J* = 7.1 Hz, 3H) ppm.

$^{13}C\{^{1}H\}$ NMR (151 MHz, $CDCl_3$): δ 174.0, 133.2, 130.4, 46.5, 41.8, 40.3, 32.2, 30.6, 26.2, 25.4, 25.2, 25.1, 24.8, 24.4, 23.9, 15.0, 13.2 ppm.

IR (neat) $\tilde{\nu}_{max}$: 2969, 2926, 2856, 1633, 1480, 1460, 1445, 1428, 1379, 1362, 1275, 1237, 1220, 1133, 1096, 1080, 979, 754, 624, 409 cm^{-1}.

HRMS (ESI^+) *m/z*: $[M+H]^+$ Calcd for $C_{17}H_{32}NO^+$ 266.2479; Found 266.2485.

(*E*)-*N*,*N*-Diethyldodec-3-enamide (3r)

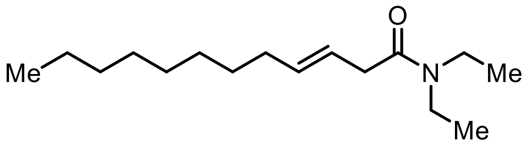

3r
$C_{16}H_{31}NO$
MW: 253.43
isolated isomer

Synthesized following **General Procedure C** using 1-undecene (103.0 µL, 0.50 mmol, 1.00 equiv.), diethylcarbamoyl chloride (127.0 µL, 2.00 mmol, 1.05 equiv.), silver hexafluoroantimonate 361.0 mg, 2.10 mmol, 1.10 equiv.) and dichloromethane (0.2 M, 2.5 mL). The crude material (57% NMR yield; 2.3:1 *E/Z*) was purified by flash column chromatography on silica gel (gradient of EtOAc in *n*-heptane from 10% to 80%) to give the title compounds (57.4 mg, 0.22 mmol, 43%, 2.1:1 *E/Z;* 38.9 mg **3r** (29%) and 18.5 mg **3r'** (14%)) as orange oils.

^{1}H NMR (400 MHz, $CDCl_3$): δ 5.59 – 5.44 (m, 2H), 3.34 (q, *J* = 7.1 Hz, 2H), 3.28 (q, *J* = 7.1 Hz, 2H), 3.03 (d, *J* = 5.3 Hz, 2H), 2.00 (dd, *J* = 13.2, 7.0 Hz, 2H), 1.37 – 1.21 (m, 12H), 1.14 (t, *J* = 7.2 Hz, 3H), 1.08 (t, *J* = 7.1 Hz, 3H), 0.85 (t, *J* = 6.8 Hz, 3H).

$^{13}C\{^{1}H\}$ NMR (101 MHz, $CDCl_3$): δ 170.8, 133.7, 123.3, 42.1, 40.1, 37.7, 32.6, 32.0, 30.0, 29.4, 29.3, 29.2, 22.8, 14.4, 14.2, 13.1.

IR (neat) $\tilde{\nu}_{max}$: 2957, 2924, 2854, 1732, 1642, 1458, 1430, 1379, 1363, 1316, 1255, 1220, 1140, 1119, 1097, 1073, 971, 772 cm^{-1}.

HRMS (ESI^{+}) *m/z*: $[M+Na]^{+}$ Calcd for $C_{16}H_{31}NONa$ 276.2303; Found 276.2296.

(*Z*)-*N*,*N*-Diethyldodec-3-enamide (3r'')

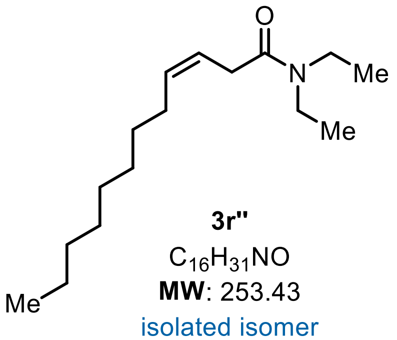

3r''
$C_{16}H_{31}NO$
MW: 253.43
isolated isomer

^{1}H NMR (400 MHz, $CDCl_3$): δ 5.61 – 5.51 (m, 2H), 3.37 (q, *J* = 7.1 Hz, 2H), 3.33 – 3.28 (m, 2H), 3.10 (d, *J* = 6.1 Hz, 2H), 2.07 – 2.02 (m, 2H), 1.39 – 1.34 (m, 2H), 1.32 – 1.23 (m, 12H), 1.18 (t, *J* = 7.1 Hz, 3H), 1.11 (dd, *J* = 9.6, 4.6 Hz, 3H), 0.88 (t, *J* = 7.1 Hz, 3H).

^{13}C{^{1}H} NMR (101 MHz, $CDCl_3$): δ 170.9, 132.7, 122.7, 42.2, 40.3, 32.7, 32.0, 29.6, 29.5, 29.5, 29.4, 27.7, 22.8, 14.5, 14.3, 13.2.

IR (neat) $\tilde{\nu}_{max}$: 2957, 2924, 2854, 1738, 1642, 1459, 1429, 1398, 1379, 1363, 1349, 1318, 1250, 1222, 1139, 1097, 1072, 800, 759 cm^{-1}.

HRMS (ESI$^+$) *m/z*: $[M+H]^+$ Calcd for $C_{16}H_{32}NO^+$ 254.2478; Found 254.2479.

(*E*)-*N*,*N*-Diethyldec-3-enamide (3s)

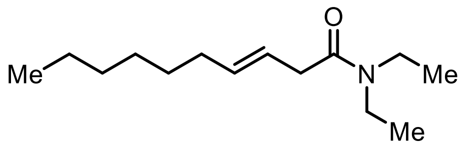

3s
$C_{14}H_{27}NO$
MW: 225.38

Synthesized following **General Procedure C** using 1-nonene (86.5 µL, 0.50 mmol, 1.00 equiv.), diethylcarbamoyl chloride (66.5 µL, 0.53 mmol, 1.05 equiv.), silver hexafluoroantimonate (189.0 mg, 0.55 mmol, 1.10 equiv.) and dichloromethane (0.2 M, 2.5 mL). The crude material (44% NMR yield, 2.5:1 *E*/*Z*) was purified by flash column chromatography on silica gel (gradient of EtOAc in *n*-heptane from 10% to 80%) to give the title compound (48.5 mg, 0.50 mmol, 43%, 2.3:1 *E*/*Z*) as a colorless oil.

^{1}H NMR (400 MHz, CDCl$_3$): δ 5.54 – 5.48 (m, 2H), 3.36 – 3.31 (q, *J* = 7.1 Hz, 2H), 3.30 – 3.24 (m, 2H), 3.07 (d, *J* = 5.4 Hz, 0.6H), 3.03 (d, *J* = 5.3 Hz, 1.40H), 2.03 – 1.97 (m, 2H), 1.34 – 1.23 (m, 10H), 1.16 (dd, *J* = 10.2, 4.1 Hz, 3H), 1.10 – 1.06 (m, 3H), 0.86– 0.82 (m, 3H).

^{13}C{^{1}H} NMR (101 MHz, CDCl$_3$): δ 170.8 (0.7C), 170.8 (0.3C), 133.77 (0.7C), 132.5 (0.3C), 123.3 (0.7C), 122.7 (0.3C), 42.1 (1C), 40.1 (1C), 37.8 (1C), 32.7 (1C), 31.8 (1C), 29.3 (1C), 29.0 (1C), 22.7 (1C), 14.5 (1C), 14.2 (1C), 13.1 (1C).

IR (neat) $\tilde{\nu}_{max}$: 2958, 2926, 2872, 2855, 1641, 1459, 1430, 1379, 1363, 1317, 1276, 1252, 1221, 1140, 1097, 1072, 969, 759 cm^{-1}.

HRMS (ESI$^+$) *m/z*: $[M+H]^+$ Calcd for $C_{14}H_{27}NO^+$ 226.2165; Found 226.2164.

(*E*)-*N*,*N*-Diethylhex-3-enamide (3t)

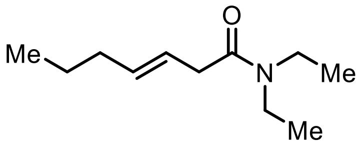

3t
$C_{11}H_{21}NO$
MW: 183.30

Synthesized following **General Procedure C** using 1-hexene (99.3 µL, 0.80 mmol, 1.00 equiv.), diethylcarbamoyl chloride (106.0 µL, 0.84 mmol, 1.05 equiv.), silver hexafluoroantimonate (302.0 mg, 0.88 mmol, 1.10 equiv.) and dichloromethane (0.2 M, 4.0 mL). The crude material (56% NMR yield, 2.2:1 *E*/*Z*) was purified by flash column chromatography on silica gel (gradient of EtOAc in *n*-heptane from 10% to 80%) to give the title compound (72.2 mg, 0.40 mmol, 61%, 2.9:1 *E*/*Z*) as a colorless oil.

^{1}H NMR (600 MHz, $CDCl_3$): δ 5.64 – 5.45 (m, 2H), 3.35 (q, *J* = 7.1 Hz, 2H), 3.29 (q, *J* = 7.2 Hz, 2H), 3.09 (d, *J* = 5.9 Hz, 0.51H), 3.04 (d, *J* = 5.8 Hz, 1.49H), 2.06 – 1.94 (m, 2H), 1.45 – 1.32 (m, 2H), 1.16 (t, *J* = 7.1 Hz, 3H), 1.10 (t, *J* = 7.1 Hz, 3H), 0.94 – 0.83 (m, 3H) ppm.

^{13}C{^{1}H} NMR (151 MHz, $CDCl_3$): δ 170.84 (0.75C), 170.82 (0.25C), 133.5 (0.75C), 132.3 (0.25C), 123.5 (0.75C), 122.9 (0.25C), 42.1 (1C), 40.2 (0.25C), 40.1 (0.75C), 37.8 (0.75C), 34.7 (0.25C), 32.7 (0.75C), 29.7 (0.25C), 22.6 (0.25C), 22.5 (0.75C), 14.46 (0.75C), 14.45 (0.25C), 13.9 (0.25C), 13.8 (0.75C), 13.14 (0.25C), 13.12 (0.75C) ppm.

IR (neat) $\tilde{\nu}_{max}$: 2962, 2931, 2873, 1636, 1457, 1429, 1379, 1363, 1316, 1251, 1221, 1140, 1097, 1072, 968, 947, 800, 701, 600, 497, 446, 419, 409 cm^{-1}.

HRMS (ESI$^+$) *m/z*: [M+H]$^+$ Calcd for $C_{11}H_{22}NO^+$ 184.1696; Found 184.1700.

(*E*)-*N*,*N*-Diethylpent-3-enamide (3u)

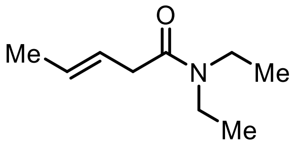

3u
$C_9H_{17}NO$
MW: 155.24

Synthesized following **General Procedure C** using 1-butene (10% in hexane) (648.0 µL, 0.80 mmol, 1.00 equiv.), diethylcarbamoyl chloride (106.0 µL, 0.84 mmol, 1.05 equiv.), silver hexafluoroantimonate (302.0 mg, 0.88 mmol, 1.10 equiv.) and dichloromethane (0.2 M, 4.0 mL). The crude material (55% NMR yield, 2:1 *E*/*Z*) was purified by flash column chromatography on silica gel (gradient of EtOAc in *n*-heptane from 10% to 80%) to give the title compound (72.2 mg, 0.40 mmol, 46%, 1.8:1 *E*/*Z*) as a colorless oil.

^{1}H NMR (600 MHz, $CDCl_3$): δ 5.66 – 5.44 (m, 2H), 3.41 – 3.22 (m, 4H), 3.12 – 3.05 (m, 0.71H), 3.05 – 2.98 (m, 1.29H), 1.72 – 1.65 (m, 2H), 1.65 – 1.58 (m, 1H), 1.15 (t, *J* = 7.4 Hz, 3H), 1.09 (t, *J* = 7.1 Hz, 3H) ppm.

^{13}C{^{1}H} NMR (151 MHz, $CDCl_3$): δ 170.79 (0.65C), 170.76 (0.35C), 128.2 (0.65C), 126.4 (0.35C), 124.6 (0.65C), 123.7 (0.35C), 42.1 (1C), 40.2 (0.35C), 40.1 (0.65C), 37.6 (0.65C), 32.3 (0.35C), 18.1 (1C), 14.43 (0.65C), 14.40 (0.35C), 13.12 (1C) ppm.

IR (neat) $\tilde{\nu}_{max}$: 3028, 2972, 2934, 2876, 1633, 1480, 1450, 1429, 1379, 1363, 1349, 1313, 1281, 1251, 1221, 1140, 1096, 1073, 1021, 967, 948, 902, 800, 682, 600, 546, 511, 499, 478, 443 cm^{-1}.

HRMS (ESI$^+$) *m/z*: $[M+H]^+$ Calcd for $C_9H_{18}NO$ 156.1383; Found 156.1386.

(*E*)-*N*,*N*,2-Triethylpent-3-enamide (3v)

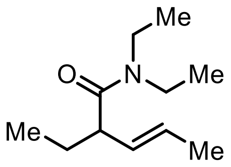

3v

$C_{11}H_{21}NO$

MW: 183.30

Synthesized following **General Procedure C** using *trans*-3-hexene (62.2 µL, 0.50 mmol, 1.00 equiv.), diethylcarbamoyl chloride (66.5 µL, 0.53 mmol, 1.05 equiv.), silver hexafluoroantimonate (189.0 mg, 0.55 mmol, 1.10 equiv.) and dichloromethane (0.2 M, 2.5 mL). The crude material (47% NMR yield, *E/Z ratio could not be identified on the crude due to overlap with impurities*) was purified by flash column chromatography on silica gel (gradient of EtOAc in *n*-heptane from 10% to 80%) to give the title compound (41.8 mg, 0.23 mmol, 46%, 6:1 *E/Z*) as a colorless oil.

^{1}H NMR (700 MHz, $CDCl_3$): δ 5.58 – 5.44 (m, 2H), 3.40 – 3.23 (m, 4.14H), 2.99 (dt, *J* = 7.1, 5.7 Hz, 0.86H), 1.81 – 1.73 (m, 1H), 1.68 – 1.65 (m, 3H), 1.53 – 1.45 (m, 1H), 1.16 (t, *J* = 7.2 Hz, 3H), 1.10 (t, *J* = 7.1 Hz, 3H), 0.85 (t, *J* = 7.4 Hz, 3H) ppm.

^{13}C{^{1}H} NMR (151 MHz, $CDCl_3$): δ 173.6 (0.14C), 173.5 (0.86C), 130.9 (0.86C), 130.4 (0.14C), 126.6 (0.86C), 124.9 (0.14C), 47.7 (0.86C), 47.1 (0.14C), 41.8 (0.86C), 41.7 (0.14C), 40.5 (0.86C), 40.3 (0.14C), 26.6 (0.14C), 26.5 (0.86C), 18.9 (0.14C), 18.0 (0.86C), 15.0 (0.86C), 14.8 (0.14C), 13.22 (0.86C), 13.18 (0.14C), 12.0 (0.86C), 11.9 (0.14C) ppm.

IR (neat) $\tilde{\nu}_{max}$: 2963, 2931, 2872, 1632, 1483, 1446, 1394, 1379, 1362, 1268, 1219, 1129, 1095, 1074, 970, 802, 750, 613, 532, 453, 433, 419 cm^{-1}.

HRMS (ESI$^+$) *m/z*: $[M+H]^+$ Calcd for $C_{11}H_{22}NO^+$ 184.1696; Found 184.1690.

(*E*)-*N*,*N*-Diethyl-5-phenylpent-3-enamide (3w)

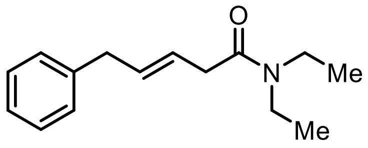

3w
$C_{15}H_{21}NO$
MW: 231.34
isolated isomer

Synthesized following **General Procedure D** using 4-phenyl-1-butene (75.1 µL, 0.50 mmol, 1.00 equiv.), diethylcarbamoyl chloride (67.3 µL, 0.53 mmol, 1.05 equiv.), silver bis(trifluoromethanesulfonyl)imide (216.0 mg, 0.55 mmol, 1.10 equiv.) and dichloromethane (0.2 M, 2.5 mL). The crude material (64% NMR yield, 3.3:1 *E*/*Z*) was purified by flash column chromatography on silica gel (gradient of EtOAc in *n*-heptane from 10% to 80%) to give the title compounds (52.7 mg, 0.23 mmol, 53%, 3.3:1 *E/Z;* 40.4 mg **3w** (41%) and 12.3 mg **3w''** (12%)) as colorless oils.

^{1}H NMR (700 MHz, $CDCl_3$): δ 7.29 – 7.26 (m, 2H), 7.20 – 7.16 (m, 3H), 5.73 – 5.65 (m, 2H), 3.41 – 3.35 (m, 4H), 3.29 (q, *J* = 7.2 Hz, 2H), 3.10 (d, *J* = 5.3 Hz, 2H), 1.15 (t, *J* = 7.2 Hz, 3H), 1.11 (t, *J* = 7.1 Hz, 3H) ppm.

$^{13}C\{^1H\}$ NMR (151 MHz, $CDCl_3$): δ 170.5, 140.5, 132.1, 128.6 (2C), 128.5 (2C), 126.1, 125.1, 42.1, 40.2, 39.1, 37.5, 14.5, 13.2 ppm.

IR (neat) $\tilde{\nu}_{max}$: 3027, 2974, 2932, 1716, 1634, 1493, 1482, 1452, 1431, 1380, 1362, 1312, 1276, 1251, 1220, 1137, 1097, 1074, 1029, 969, 771, 748, 699, 668 cm^{-1}.

HRMS (ESI$^+$) *m/z*: $[M+H]^+$ Calcd for $C_{15}H_{21}NO^+$ 231.1623; Found 231.1612.

(*Z*)-*N*,*N*-Diethyl-5-phenylpent-3-enamide (3w'')

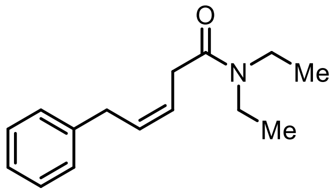

3w''
$C_{15}H_{21}NO$
MW: 231.34
isolated isomer

^{1}H NMR (700 MHz, CDCl$_3$): δ 7.30 – 7.27 (m, 2H), 7.21 – 7.18 (m, 3H), 5.80 – 5.74 (m, 2H), 3.44 (d, *J* = 4.9 Hz, 2H), 3.39 (q, *J* = 7.1 Hz, 2H), 3.30 (q, *J* = 7.2 Hz, 2H), 3.20 (d, *J* = 4.8 Hz, 2H), 1.17 (t, *J* = 7.1 Hz, 3H), 1.12 (t, *J* = 7.1 Hz, 3H) ppm.

^{13}C{^{1}H} NMR (151 MHz, CDCl$_3$): δ 170.4, 140.5, 130.6 (2C), 128.6, 128.5, 126.2 (2C), 124.1, 42.2, 40.3, 33.9, 32.5, 14.5, 13.2 ppm.

IR (neat) $\tilde{\nu}_{max}$: 3026, 2974, 2932, 2874, 1715,1634, 1482, 1451, 1432, 1380, 1362, 1314, 1257, 1219, 1176, 1139, 1097, 1072, 1029, 967, 950, 858, 828, 746, 698 cm^{-1}.

HRMS (ESI$^+$) *m/z*: [M+H]$^+$ Calcd for $C_{15}H_{21}NO^+$ 231.1623; Found 231.1618.

(*E*)-*N*,*N*-Diethyl-6-phenylhex-3-enamide (3x)

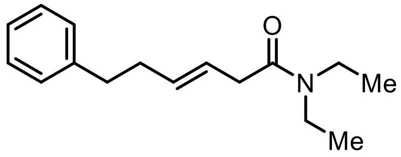

3x
$C_{16}H_{23}NO$
MW: 245.37
isolated isomer

Synthesized following **General Procedure C** using 4-phenyl-1-butene (173.0 µL, 1.00 mmol, 1.00 equiv.), diethylcarbamoyl chloride (133.0 µL, 1.05 mmol, 1.05 equiv.), silver hexafluoroantimonate (378.0 mg, 1.1 mmol, 1.10 equiv.) and dichloromethane (0.2 M, 5.0 mL). The crude material (56% NMR yield, 2.2:1 *E*/*Z*) was purified by flash column chromatography on silica gel (gradient of EtOAc in *n*-heptane from 10% to 80%) to give the title compounds (128.6 mg, 0.53 mmol, 53%, 2.2:1 *E*/*Z;* 88.4 mg **3x** (36%) and 40.2 mg **3x''** (17%)) as colorless oils.

^{1}H NMR (700 MHz, $CDCl_3$): δ 7.26 (dd, *J* = 8.6, 6.6 Hz, 2H), 7.21 – 7.14 (m, 3H), 5.69 – 5.49 (m, 2H), 3.36 (q, *J* = 7.1 Hz, 2H), 3.26 (q, *J* = 7.1 Hz, 2H), 3.04 (d, *J* = 6.1 Hz, 2H), 2.75 – 2.64 (m, 2H), 2.37 (m, 2H), 1.15 (t, *J* = 7.1 Hz, 3H), 1.10 (t, *J* = 7.1 Hz, 3H) ppm.

$^{13}C\{^{1}H\}$ NMR (151 MHz, $CDCl_3$): δ 170.7, 142.0, 132.6 (2C), 128.6, 128.4, 125.9 (2C), 124.2, 42.1, 40.2, 37.6, 35.8, 34.4, 14.5, 13.2 ppm.

IR (neat) $\tilde{\nu}_{max}$: 3027, 2973, 2932, 1633, 1495, 1479, 1454, 1430, 1379, 1363, 1312, 1281, 1251, 1221, 1135, 1096, 1071, 1030, 968, 923, 910, 772, 729, 698, 644 cm^{-1}.

HRMS (ESI^{+}) *m/z*: $[M+H]^{+}$ Calcd for $C_{16}H_{24}NO^{+}$ 246.1852; Found 246.1858.

(*Z*)-*N*,*N*-Diethyl-6-phenylhex-3-enamide (3x'')

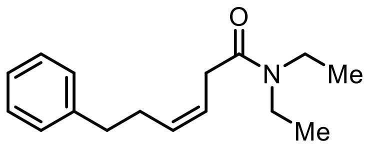

3x''
$C_{16}H_{23}NO$
MW: 245.37
isolated isomer

^{1}H NMR (700 MHz, $CDCl_3$): δ 7.31 – 7.24 (m, 2H), 7.22 – 7.15 (m, 3H), 5.67 – 5.55 (m, 2H), 3.36 (q, *J* = 7.2 Hz, 2H), 3.21 (q, *J* = 7.1 Hz, 2H), 2.96 (d, *J* = 4.9 Hz, 2H), 2.69 (t, *J* = 7.6 Hz, 2H), 2.42 – 2.33 (m, 2H), 1.16 – 1.06 (m, 6H) ppm.

^{13}C{^{1}H} NMR (151 MHz, $CDCl_3$): δ 170.6, 141.9, 131.1 (2C), 128.6, 128.4, 126.0 (2C), 123.7, 42.1, 40.2, 35.7, 32.4, 29.7, 14.5, 13.2 ppm.

IR (neat) $\tilde{\nu}_{max}$: 3025, 2972, 2931, 2873, 1634, 1480, 1453, 1429, 1379, 1362, 1348, 1316, 1251, 1220, 1135, 1096, 1082, 1071, 1029, 948, 924, 909, 798, 772, 750, 730, 699 cm^{-1}.

HRMS (ESI$^+$) *m/z*: $[M+H]^+$ Calcd for $C_{16}H_{24}NO^+$ 246.1852; Found 246.1858.

(*E*)-*N*,*N*-Diethyl-6-phenylhex-3-enamide (3y)

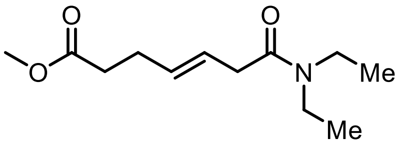

3y
$C_{12}H_{21}NO_3$
MW: 227.30
isolated isomer

Synthesized following **General Procedure C** using methyl-5-hexenoate (70.2 µL, 0.50 mmol, 1.00 equiv.), diethylcarbamoyl chloride (127.0 µL, 1.00 mmol, 2.00 equiv.), silver hexafluoroantimonate (361.0 mg, 1.05 mmol, 2.10 equiv.) and dichloromethane (0.2 M, 2.5 mL). The crude material (64% NMR yield, 3.2:1 *E*/*Z*) was purified by flash column chromatography on silica gel (gradient of EtOAc in *n*-heptane from 10% to 80%) to give the title compounds (54.7 mg, 0.24 mmol, 48%, 3:1 *E*/*Z;* 41.0 mg **3y** (36%) and 13.7 mg **3y'** (12%)) as colorless oils.

^{1}H NMR (400 MHz, CDCl$_3$): δ 5.64 (ddd, *J* = 12.8, 9.6, 5.9 Hz, 1H), 5.51 (ddd, *J* = 13.9, 8.7, 3.8 Hz, 1H), 3.65 (s, 3H), 3.35 (q, *J* = 7.1 Hz, 2H), 3.27 (q, *J* = 7.1 Hz, 2H), 3.04 (dd, *J* = 6.4, 0.8 Hz, 2H), 2.42 – 2.32 (m, 4H), 1.15 (t, *J* = 7.1 Hz, 3H), 1.10 (t, *J* = 7.1 Hz, 3H) ppm.

^{13}C{^{1}H} NMR (101 MHz, CDCl$_3$): δ 173.7, 170.5, 131.3, 125.0, 51.7, 42.2, 40.2, 37.5, 33.9, 27.9, 14.5, 13.2 ppm.

IR (neat) $\tilde{\nu}_{max}$: 2973, 2933, 1734, 1632, 1480, 1433, 1378, 1362, 1315, 1252, 1219, 1196, 1161, 1138, 1095, 1080, 1022, 970, 906, 880, 835, 773, 711, 688, 658, 599, 559, 539, 527, 504, 446, 436, 404 cm^{-1}.

HRMS (ESI$^+$) *m/z*: [M+H]$^+$ Calcd for $C_{12}H_{22}NO_3^+$ 228.1594; Found 228.1595.

(*Z*)-*N*,*N*-Diethyl-6-phenylhex-3-enamide (3y'')

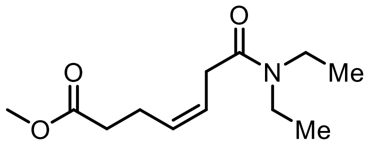

3y''
$C_{12}H_{21}NO_3$
MW: 227.30
isolated isomer

^{1}H NMR (400 MHz, $CDCl_3$): δ 5.68 (dt, *J* = 10.7, 6.9 Hz, 1H), 5.51 (dtd, *J* = 10.5, 4.4, 2.3 Hz, 1H), 3.67 (s, 3H), 3.36 (m, 2H), 3.34 – 3.28 (m, 2H), 3.14 – 3.12 (m, 2H), 2.39 (m, 4H), 1.19 (t, *J* = 7.1 Hz, 3H), 1.11 (t, *J* = 7.1 Hz, 3H) ppm.

^{13}C{^{1}H} NMR (101 MHz, $CDCl_3$): δ 170.4, 168.3, 130.0, 124.5, 51.7, 42.2, 40.3, 33.8, 32.3, 23.2, 14.5, 13.2 ppm.

IR (neat) $\tilde{\nu}_{max}$: 2970, 2932, 1735, 1633, 1433, 1363, 1304, 1251, 1219, 1196, 1159, 1136, 1080, 1043, 1024, 969, 948, 906, 875, 834, 772, 688, 666, 598, 540, 526, 481, 443, 434, 422 cm^{-1}.

HRMS (ESI$^+$) *m/z*: $[M+H]^+$ Calcd for $C_{12}H_{22}NO_3^+$ 228.1594; Found 228.1595.

(*E*)-7-Bromo-*N*,*N*-diethylhept-3-enamide (3z)

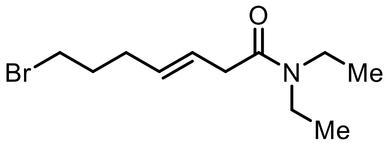

3z
$C_{11}H_{20}BrNO$
MW: 262.19
isolated isomer

Synthesized following **General Procedure C** using 6-bromo-1-hexene (67.0 µL, 0.50 mmol, 1.00 equiv.), diethylcarbamoyl chloride (127.0 µL, 1.00 mmol, 2.00 equiv.), silver hexafluoroantimonate (361.0 mg, 1.05 mmol, 2.10 equiv.) and dichloromethane (0.2 M, 2.5 mL). The crude material (64% NMR yield, 3.2:1 *E*/*Z*) was purified by flash column chromatography on silica gel (gradient of EtOAc in *n*-heptane from 10% to 80%) to give the title compounds (77.6 mg, 0.30 mmol, 59%, 2.5:1 *E/Z;* 55.4 mg **3z** (42%) and 22.2 mg **3z''** (17%)) as colorless oils.

^{1}H NMR (400 MHz, $CDCl_3$): δ 5.64 (dt, *J* = 14.3, 6.5 Hz, 1H), 5.47 (dt, *J* = 15.2, 6.7 Hz, 1H), 3.41 – 3.25 (m, 6H), 3.04 (dd, *J* = 6.5, 0.9 Hz, 2H), 2.22 – 2.15 (m, 2H), 1.96 – 1.87 (m, 2H), 1.16 (t, *J* = 7.2 Hz, 3H), 1.09 (t, *J* = 7.1 Hz, 3H) ppm.

^{13}C{^{1}H} NMR (101 MHz, $CDCl_3$): δ 170.5, 131.3, 125.2, 42.1, 40.2, 37.5, 33.3, 32.2, 30.9, 14.5, 13.1 ppm.

IR (neat) $\tilde{\nu}_{max}$: 2971, 2932, 2873, 1751, 1711, 1633, 1479, 1429, 1379, 1362, 1349, 1312, 1279, 1248, 1220, 1125, 1096, 1071, 1049, 1021, 967, 905, 773, 645, 598, 559, 497, 485, 473, 450, 435, 404 cm^{-1}.

HRMS (ESI^{+}) *m/z*: [M+H]$^{+}$ Calcd for $C_{11}H_{21}{}^{79}BrNO^{+}$ 262.0801; Found 262.0797.

(*Z*)-7-Bromo-*N*,*N*-diethylhept-3-enamide (3z'')

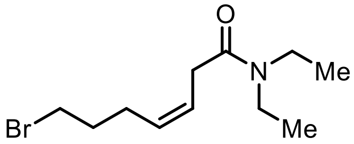

3z''
$C_{11}H_{20}BrNO$
MW: 262.19
isolated isomer

^{1}H NMR (400 MHz, CDCl$_3$): δ 5.71 (ddd, *J* = 10.8, 6.9, 1.4 Hz, 1H), 5.50 (ddd, *J* = 10.7, 9.1, 7.4 Hz, 1H), 3.43 (t, *J* = 6.4 Hz, 2H), 3.41 – 3.29 (m, 4H), 3.15 (dd, *J* = 6.9, 0.8 Hz, 2H), 2.25 (d, *J* = 7.3 Hz, 2H), 1.99 – 1.23 (m, 2H), 1.20 (t, *J* = 7.1 Hz, 3H), 1.11 (t, *J* = 7.1 Hz, 3H) ppm.

^{13}C{^{1}H} NMR (101 MHz, CDCl$_3$): δ 170.5, 130.1, 124.8, 42.2, 40.3, 33.7, 32.4, 32.2, 25.9, 14.5, 13.2 ppm.

IR (neat) $\tilde{\nu}_{max}$: 2972, 2933, 1735, 1632, 1480, 1433, 1379, 1362, 1315, 1252, 1219, 1197, 1162, 1138, 1096, 1080, 1024, 970, 949, 834, 773, 708, 687, 660, 599, 558, 496, 476, 436, 423 cm^{-1}.

HRMS (ESI$^+$) *m/z*: [M+H]$^+$ Calcd for $C_{11}H_{21}{}^{79}BrNO^+$ 262.0801; Found 262.0795.

(*E*)-8-(1,3-Dioxoisoindolin-2-yl)-*N*,*N*-diethyloct-3-enamide (3aa)

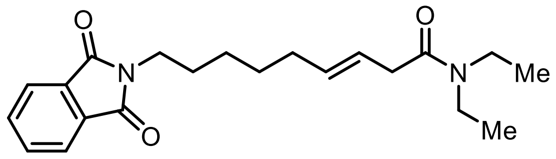

3aa
$C_{21}H_{28}N_2O_3$
MW: 356.47

Synthesized following **General Procedure C** using 2-(oct-7-en-1-yl)isoindoline-1,3-dione (**1aa**) (129.0 mg, 0.50 mmol, 1.00 equiv.), diethylcarbamoyl chloride (127.0 µL, 1.00 mmol, 2.00 equiv.), silver hexafluoroantimonate (361.0 mg, 1.05 mmol, 2.10 equiv.) and dichloromethane (0.2 M, 2.5 mL). The crude material (72% NMR yield, 4:1 *E*/*Z*) was purified by flash column chromatography on silica gel (gradient of EtOAc in *n*-heptane from 10% to 80%) to give the title compound (127.0 mg, 0.36 mmol, 71%, 4:1 *E*/*Z*) as a colorless oil.

^{1}H NMR (400 MHz, CDCl$_3$): δ 7.85 – 7.79 (m, 2H), 7.72 – 7.66 (m, 2H), 5.62 – 5.40 (m, 2H), 3.70 – 3.61 (m, 2H), 3.34 (q, *J* = 7.1 Hz, 2H), 3.28 (q, *J* = 7.1 Hz, 2H), 3.07 (d, *J* = 6.3 Hz, 0.39H), 3.02 (d, *J* = 6.0 Hz, 1.58H), 2.02 (dd, *J* = 13.9, 7.0 Hz, 2H), 1.70 – 1.60 (m, 2H), 1.45 – 1.29 (m, 4H), 1.15 (t, *J* = 7.1 Hz, 3H), 1.09 (t, *J* = 7.1 Hz, 3H) ppm.

^{13}C{^{1}H} NMR (101 MHz, CDCl$_3$): δ 170.7 (1C), 168.5 (2C), 133.98 (0.4C), 133.95 (1.6C), 133.3 (0.8C), 132.3 (1.6C), 132.0 (0.2C), 131.6 (0.4C), 123.7 (0.8C), 123.3 (2C), 123.1 (0.2C), 42.1 (0.8C), 41.8 (0.2C), 40.2 (0.2C), 40.1 (0.8C), 38.1 (0.8C), 38.0 (0.2C), 37.7 (0.8C), 32.6 (0.2C), 32.5 (0.8C), 29.0 (0.2C), 28.9 (0.8C), 28.5 (1C), 27.5 (0.2C), 26.6 (0.2C), 26.5 (0.8C), 15.0 (0.2C), 14.5 (0.8C), 13.2 (0.2C), 13.1 (0.8C) ppm.

IR (neat) $\tilde{\nu}_{max}$: 2972, 2932, 2858, 1771, 1707, 1632, 1462, 1434, 1394, 1362, 1276, 1250, 1219, 1187, 1171, 1139, 1070, 1046, 969, 948, 888, 873, 793, 750, 718, 693, 664, 618, 602, 529, 495, 476, 451, 435, 411 cm^{-1}.

HRMS (ESI$^+$) *m/z*: [M+H]$^+$ Calcd for $C_{21}H_{29}N_2O_3^+$ 357.2173; Found 357.2163.

7.3. Further Functionalization

7.3.1. Synthesis of α,β-Unsaturated γ-OTMP Amides

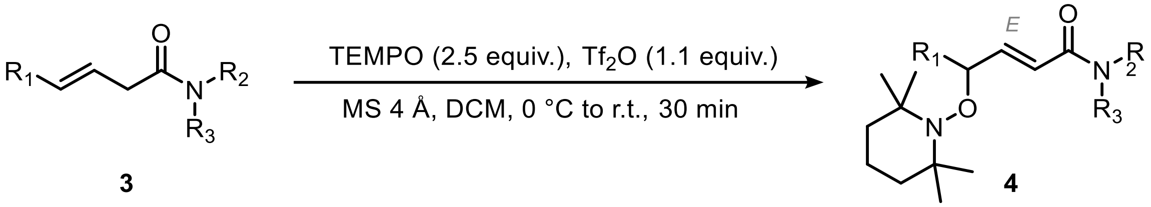

General Procedure E

To a flame-dried Schlenk flask under an atmosphere of argon containing molecular sieves (4 Å, 4.7 equiv.), were added the *E*/*Z* amide mixture (1.0 equiv.) and anhydrous dichloromethane (0.1 M). TEMPO (2.5 equiv.) was then added and the reaction mixture was cooled in an ice-water bath. Trifluoromethanesulfonic anhydride (Tf_2O, 1.1 equiv.) was subsequently added, the cooling bath was removed, and the reaction was stirred at r.t. for 30 min. Then, the reaction mixture was filtered, removing the molecular sieves, and added to a flask containing a saturated aqueous solution of $NaHCO_3$ (2.5 mL/mmol). The mixture was vigorously stirred at r.t. for 15 min, before the phases were separated, and the aqueous phase was extracted with dichloromethane (3 × 30 mL/mmol). The combined organic layers were washed with brine (1 × 10 mL) and dried over anhydrous magnesium sulfate. After filtration, the solvent was removed *in vacuo* and the crude material was analyzed by NMR adding mesitylene (0.50 equiv.) as an internal standard to calculate the NMR yield (%). The crude product was further purified by flash column chromatography on silica gel, employing a gradient of EtOAc in *n*-heptane from 10% to 80%, to afford the final compounds.

(*E*)-1-(Pyrrolidin-1-yl)-4-((2,2,6,6-tetramethylpiperidin-1-yl)oxy)dodec-2-en-1-one (4a)

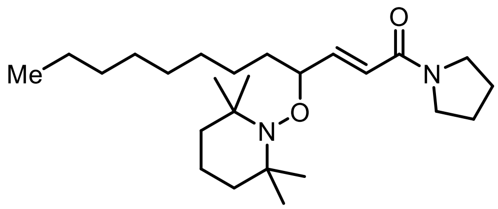

4a
$C_{25}H_{48}N_2O_2$
MW: 408.67

Synthesized following **General Procedure E** using a crude *E*/*Z* mixture 2.9:1 of 1-(pyrrolidin-1-yl)dodec-3-en-1-one (**3ab**) (74.4 mg, 0.30 mmol, 1.00 equiv.), molecular sieves (4 Å, 198 mg, 1.4 mmol, 4.7 equiv.), TEMPO (119.0 mg, 0.74 mmol, 2.5 equiv.), trifluoromethanesulfonic anhydride (54.9 µL, 0.33 mmol, 1.10 equiv.) and dichloromethane (0.1 M, 3.0 mL). The crude material was purified by flash column chromatography on silica gel (gradient of EtOAc in *n*-heptane from 10% to 80%) to give the title compound (96.9 mg, 0.24 mmol, 81%) as a yellow oil.

^{1}H NMR (400 MHz, $CDCl_3$): δ 6.86 (dd, *J* = 15.2, 8.0 Hz, 1H), 6.14 (d, *J* = 15.2 Hz, 1H), 4.26 (td, *J* = 7.9, 4.7 Hz, 1H), 3.60 – 3.46 (m, 4H), 2.01 – 1.92 (m, 2H), 1.91 – 1.82 (m, 2H), 1.75 (dd, *J* = 13.3, 4.5 Hz, 1H), 1.60 – 1.49 (m, 2H), 1.41 (s, 4H), 1.33 – 1.20 (m, 15H), 1.18 – 1.05 (m, 12H), 0.87 (t, *J* = 6.9 Hz, 3H) ppm.

^{13}C{^{1}H} NMR (101 MHz, $CDCl_3$): δ 164.5, 146.8, 121.3, 83.9, 60.0, 59.4, 46.4, 45.7, 40.1, 40.01, 33.95, 31.8, 29.7, 29.4, 29.2, 26.0, 24.9, 24.2, 22.5, 20.3 (2C), 20.2 (2C), 17.1, 14.0 ppm.

IR (neat) $\tilde{\nu}_{max}$: 2925, 2870, 2855, 1715, 1661, 1619, 1423, 1396, 1374, 1359, 1337, 1299, 1255, 1242, 1228, 1182, 1132, 1043, 1010, 983, 972, 956, 751, 720, 664, 576, 528, 510, 484, 475, 458, 439, 414, 403 cm^{-1}.

HRMS – (ESI$^+$) *m/z*: [M+H]$^+$ Calcd for $C_{25}H_{47}N_2O_2^+$ 407.3632; Found 407.3627.

7.3.2. Synthesis of additional α,β-Unsaturated γ-OTMP Amides

(*E*)-*N*,*N*-Diethyl-4-((2,2,6,6-tetramethylpiperidin-1-yl)oxy)hept-2-enamide (4b)

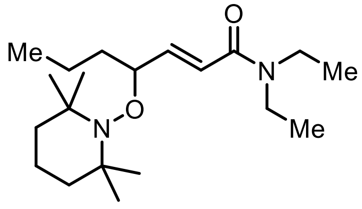

4b
$C_{20}H_{38}N_2O_2$
MW: 338.54

Synthesized following **General Procedure E** using a crude *E*/*Z* mixture 2.6:1 of *N*,*N*-diethylhept-3-enamide (**3t**) (55.9 mg, 0.31 mmol, 1.00 equiv.), molecular sieves (4 Å, 217.0 mg, 1.52 mmol, 4.7 equiv.), TEMPO (124.0 mg, 0.78 mmol, 2.5 equiv.), trifluoromethanesulfonic anhydride (59.9 µL, 0.36 mmol, 1.10 equiv.) and dichloromethane (0.1 M, 3.0 mL). The crude material was purified by flash column chromatography on silica gel (gradient of EtOAc in *n*-heptane from 10% to 80%) to give the title compound (84.9 mg, 0.25 mmol, 82%) as a yellow oil.

^{1}H NMR (400 MHz, $CDCl_3$): δ 6.84 (dd, *J* = 15.1, 7.9 Hz, 1H), 6.26 (d, *J* = 15.1 Hz, 1H), 4.30 (td, *J* = 7.8, 4.9 Hz, 1H), 3.54 – 3.31 (m, 4H), 1.81 – 1.69 (m, 1H), 1.63 – 1.49 (m, 2H), 1.40 (d, *J* = 18.2 Hz, 4H), 1.38 – 1.26 (m, 3H), 1.25 – 1.11 (m, 13H), 1.08 (m, 5H), 0.95 – 0.85 (m, 3H) ppm.

$^{13}C\{^1H\}$ NMR (101 MHz, $CDCl_3$): δ 165.3, 146.7, 120.2, 83.4, 59.7, 59.2, 41.8, 40.4, 39.9 (2C), 35.8, 34.4, 33.8, 20.0 (2C), 17.9, 16.9, 14.6, 13.8, 12.9 ppm.

IR (neat) $\tilde{\nu}_{max}$: 2965, 2930, 2871, 1660, 1620, 1446, 1426, 1375, 1359, 1273, 1241, 1220, 1182, 1132, 1096, 1080, 1044, 982, 973, 956, 925, 774, 751, 721, 529, 509, 477, 420 cm^{-1}.

HRMS – (ESI$^+$) *m/z*: $[M+H]^+$ Calcd for $C_{20}H_{39}N_2O_2^+$ 339.3006; Found 339.3001.

7.3.3. Synthesis of β-Fluoro-α,β-Unsaturated Amides

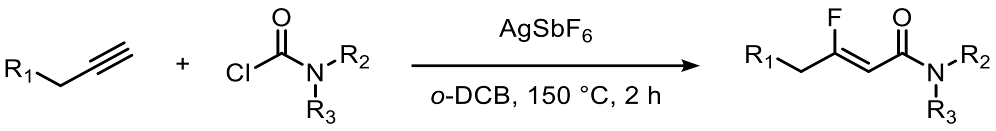

5 **2** **6**

General Procedure F

Alkyne (**5**) (1.00 equiv.) and carbamoyl chloride (**2**) (1.05 equiv.) were dissolved in *o*-DCB (0.2 M). To this, $AgSbF_6$ (1.10 equiv.) was added, and the mixture was placed in a pre-heated oil bath and allowed to stir at the same temperature. Following this time, triethylamine was added and the resulting mixture was stirred for 10 min while allowed to cool to room temperature. Triethylamine was removed *in vacuo* and the crude material was purified by flash column chromatography on silica gel employing a gradient of EtOAc in *n*-heptane from 0% to 80%, to afford the final compounds (**6**).

(*Z*)-*N*,*N*-Diethyl-3-fluorohex-2-enamide (6a)

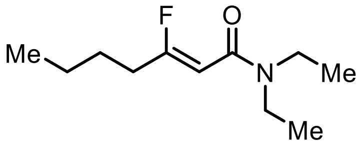

6a
$C_{11}H_{20}FNO$
MW: 201.29

Synthesized following **General Procedure F** using 1-hexyne (46.0 µL, 0.40 mmol, 1.00 equiv.), diethylcarbamoyl chloride (53.2 µL, 0.42 mmol, 1.05 equiv.), silver hexafluoroantimonate (151.0 mg, 0.44 mmol, 1.1 equiv.), *ortho*-dichlorobenzene (0.2 M, 2.0 mL). The crude material was purified by flash column chromatography on silica gel (gradient of EtOAc in *n*-heptane from 10% to 80%) to give the title compound (23.6 mg, 0.12 mmol, 29%) as a colorless oil.

^{1}H NMR (600 MHz, $CDCl_3$): δ 5.24 (d, *J* = 36.9 Hz, 1H), 3.41 (q, *J* = 7.1 Hz, 2H), 3.33 (q, *J* = 7.1 Hz, 2H), 2.29 – 2.18 (m, 2H), 1.52 (dt, *J* = 15.2, 7.5 Hz, 2H), 1.42 – 1.33 (m, 2H), 1.18 – 1.09 (m, 6H), 0.92 (t, *J* = 7.4 Hz, 3H) ppm.

^{13}C{^{1}H} NMR (151 MHz, $CDCl_3$): δ 165.0 (d, *J* = 364.9 Hz), 164.5, 100.4 (d, *J* = 12.4 Hz), 42.8, 39.5, 32.2 (d, *J* = 25.5 Hz), 27.92 (*J* = 1.4 Hz), 22.2, 14.4, 13.8, 13.1 ppm.

^{19}F NMR (659 MHz, $CDCl_3$): δ 91.41 ppm.

IR (neat) $\tilde{\nu}_{max}$: 2961, 2931, 2872, 1689, 1660, 1623, 1458, 1427, 1378, 1360, 1321, 1262, 1221, 1133, 1096, 1080, 983, 956, 888, 824, 807, 774, 510, 493, 480, 452, 435, 423 cm^{-1}.

HRMS – (ESI$^+$) *m/z*: $[M+Na]^+$ Calcd for $C_{11}H_{20}FNONa^+$ 224.1421; Found 224.1419.

8. NMR Spectra

3,4-Dihydroisoquinoline-2(1*H*)-carbonyl chloride (2f)

^{1}H NMR (400 MHz, $CDCl_3$):

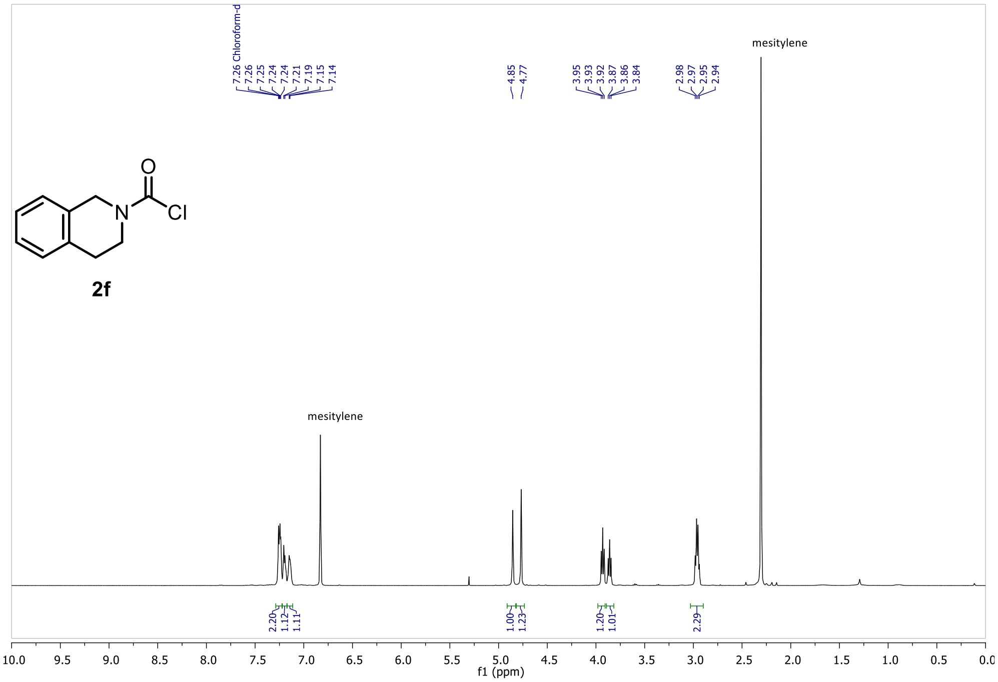

^{13}C{^{1}H} NMR (101 MHz, $CDCl_3$):

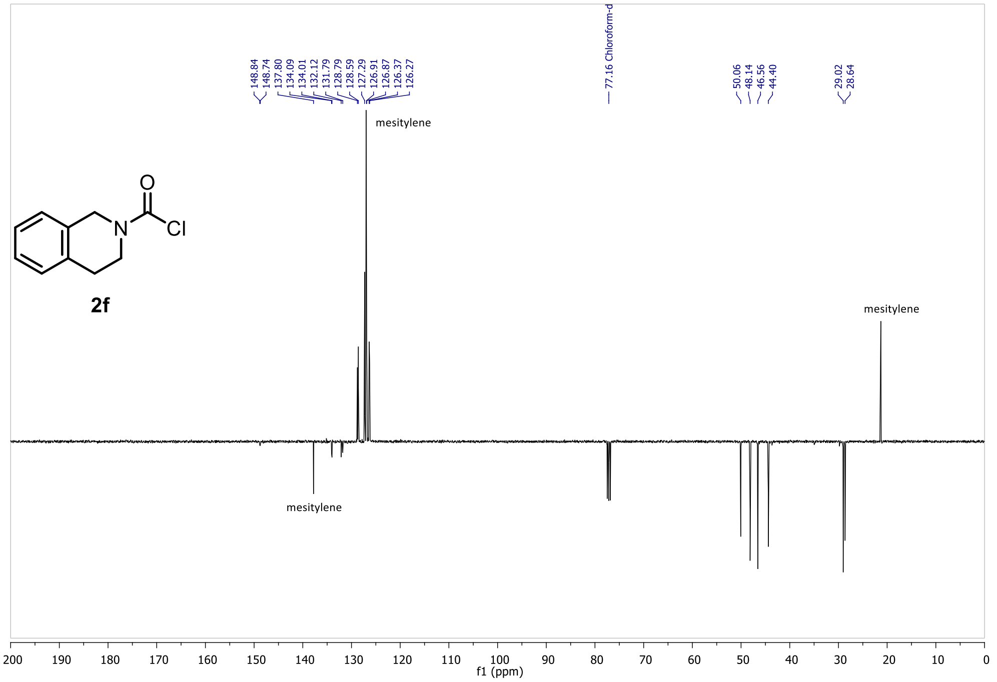

N-Methyl-*N*-phenylcarbamoyl chloride (2h)

^{1}H NMR (400 MHz, $CDCl_3$):

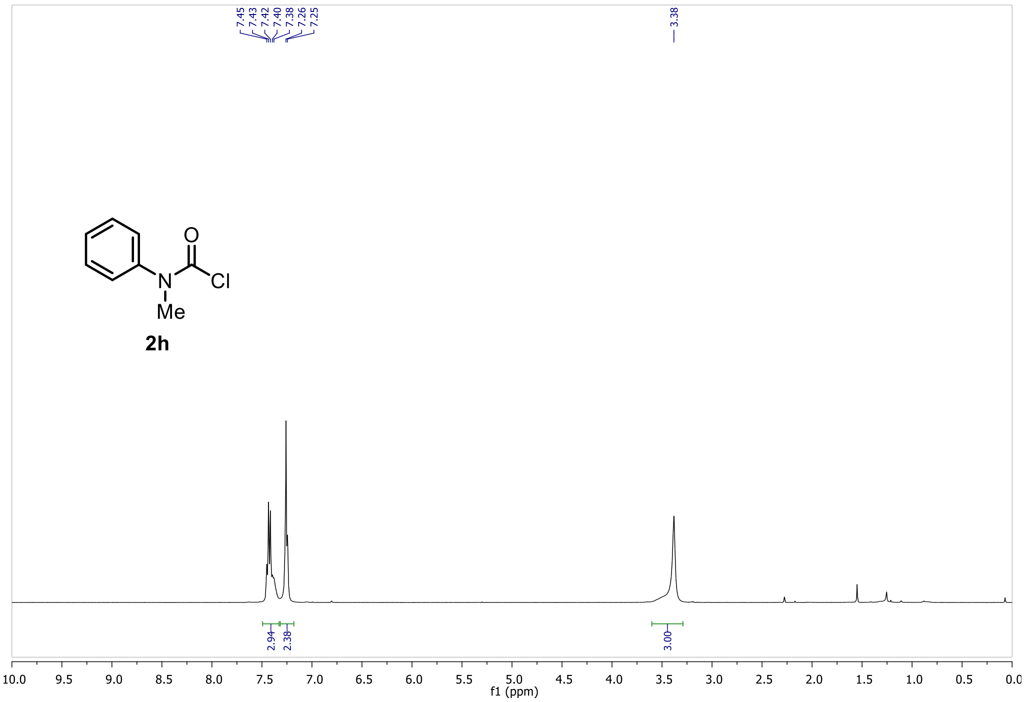

^{13}C{^{1}H} NMR (101 MHz, $CDCl_3$):

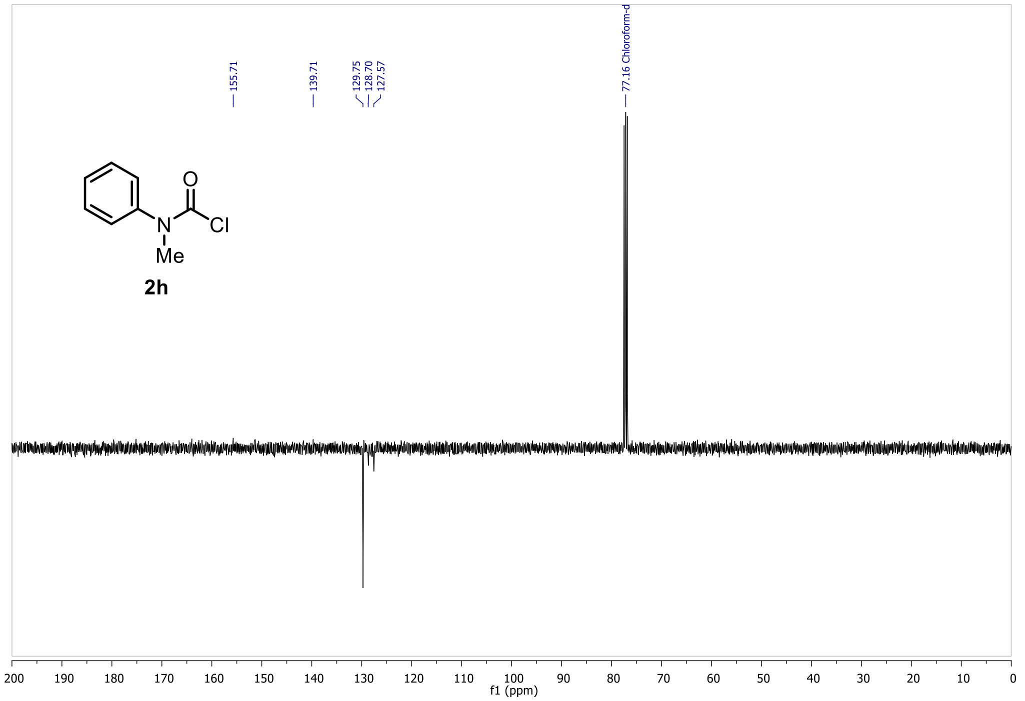

***N*-Methyl-*N*-tosylcarbamoyl chloride (2ac)**

^{1}H NMR (400 MHz, $CDCl_3$):

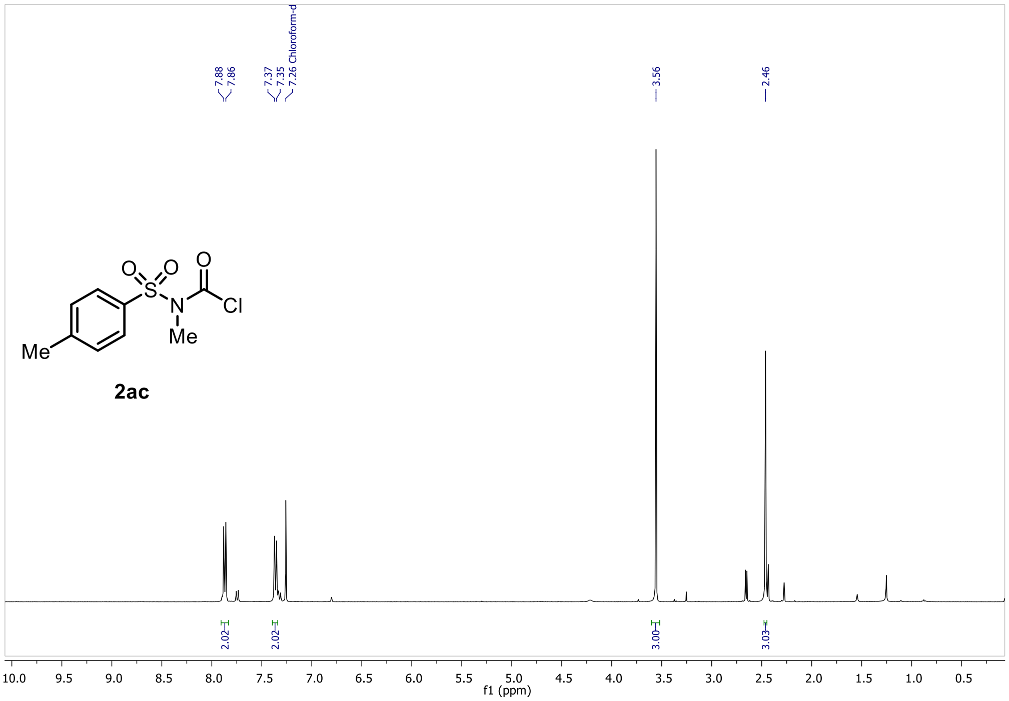

^{13}C{^{1}H} NMR (101 MHz, $CDCl_3$):

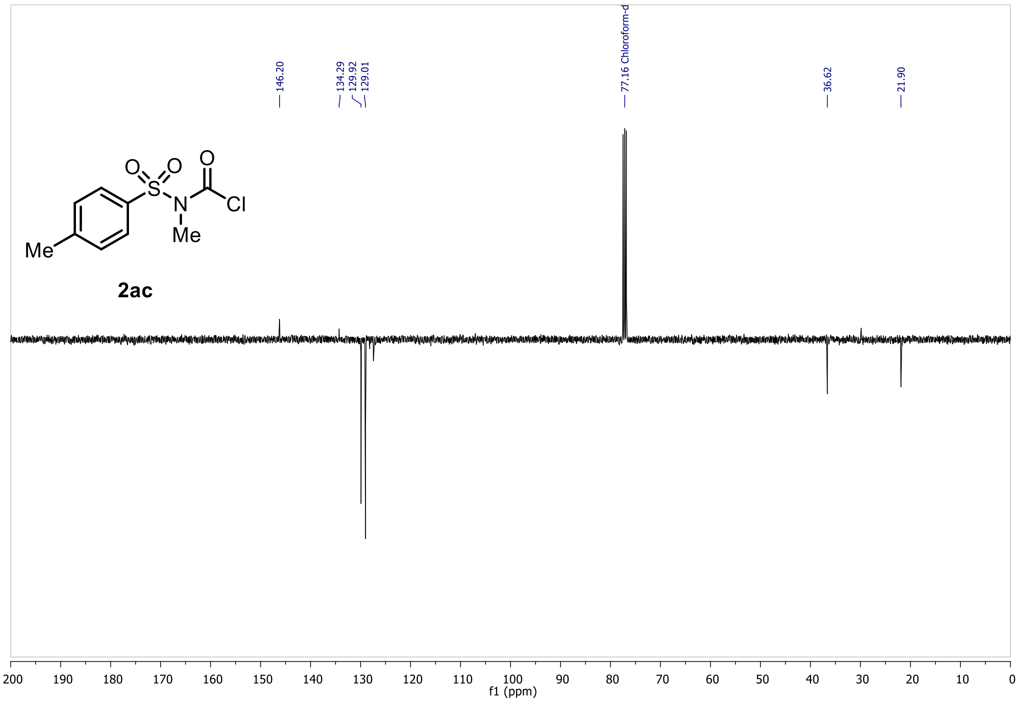

N-Methyl-*N*-nosylcarbamoyl chloride (2ad)

^{1}H NMR (400 MHz, $CDCl_3$):

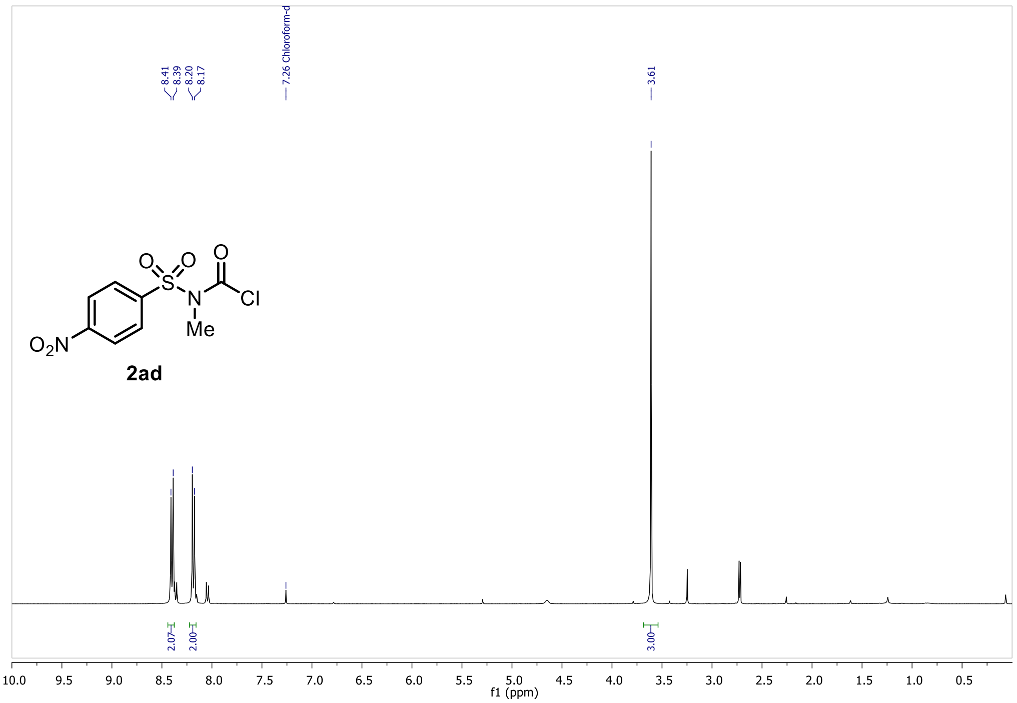

^{13}C{^{1}H} NMR (101 MHz, $CDCl_3$):

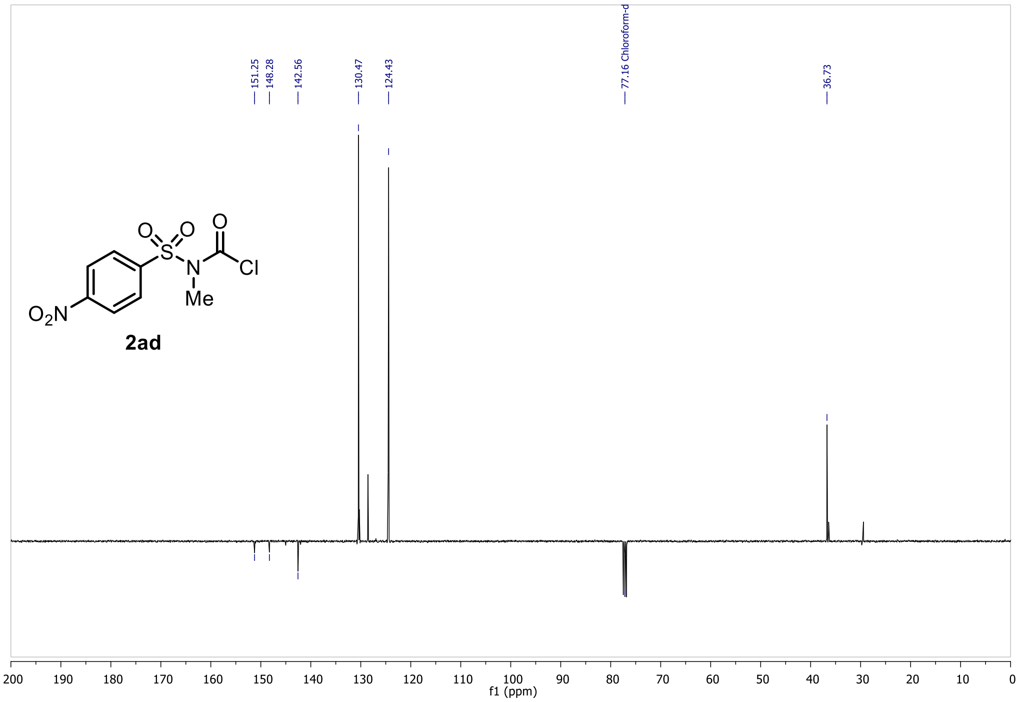

***N,N*-Diethylcyclohex-2-ene-1-carboxamide (3a)**

^{1}H NMR (400 MHz, CDCl$_3$):

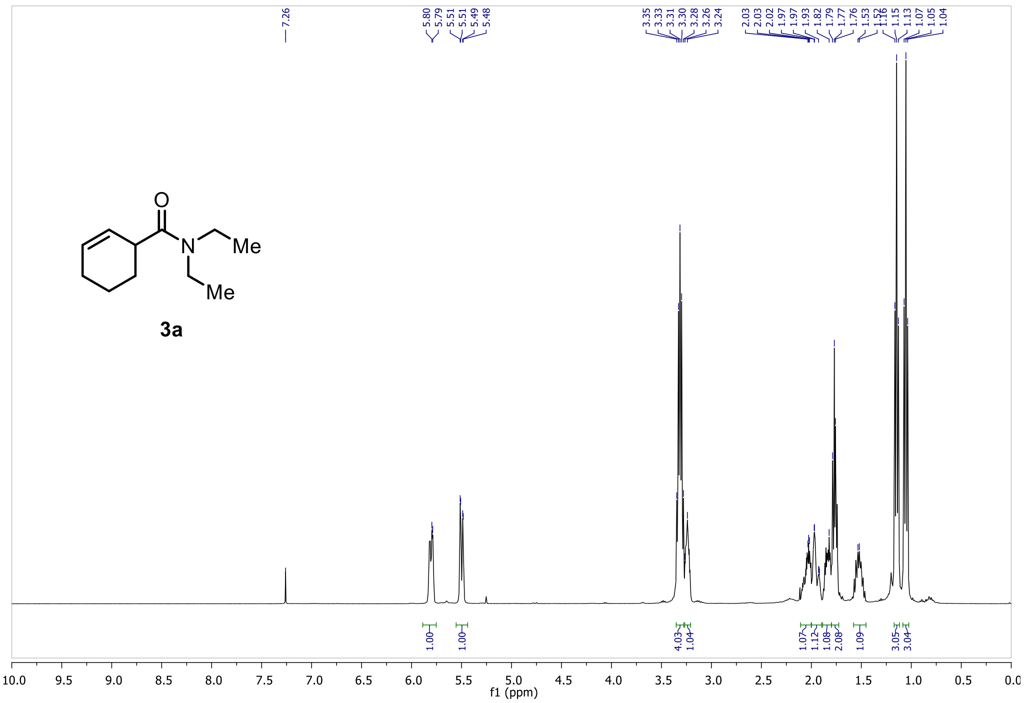

^{13}C{^{1}H} NMR (101 MHz, CDCl$_3$):

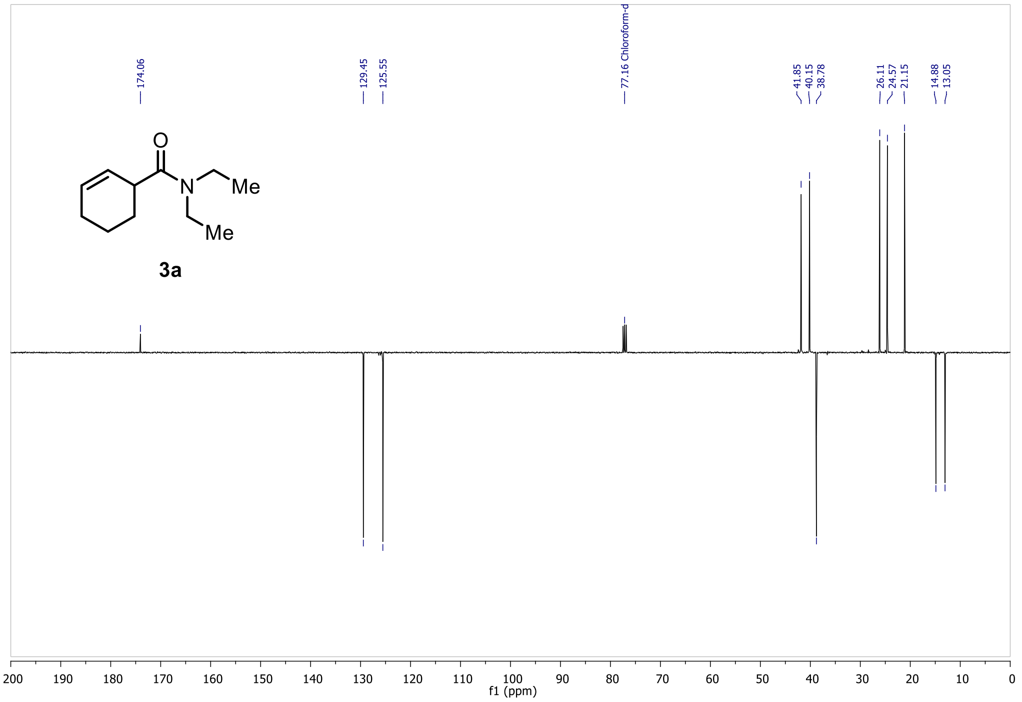

N,N-Dimethylcyclohex-2-ene-1-carboxamide (3b)

^{1}H NMR (400 MHz, $CDCl_3$):

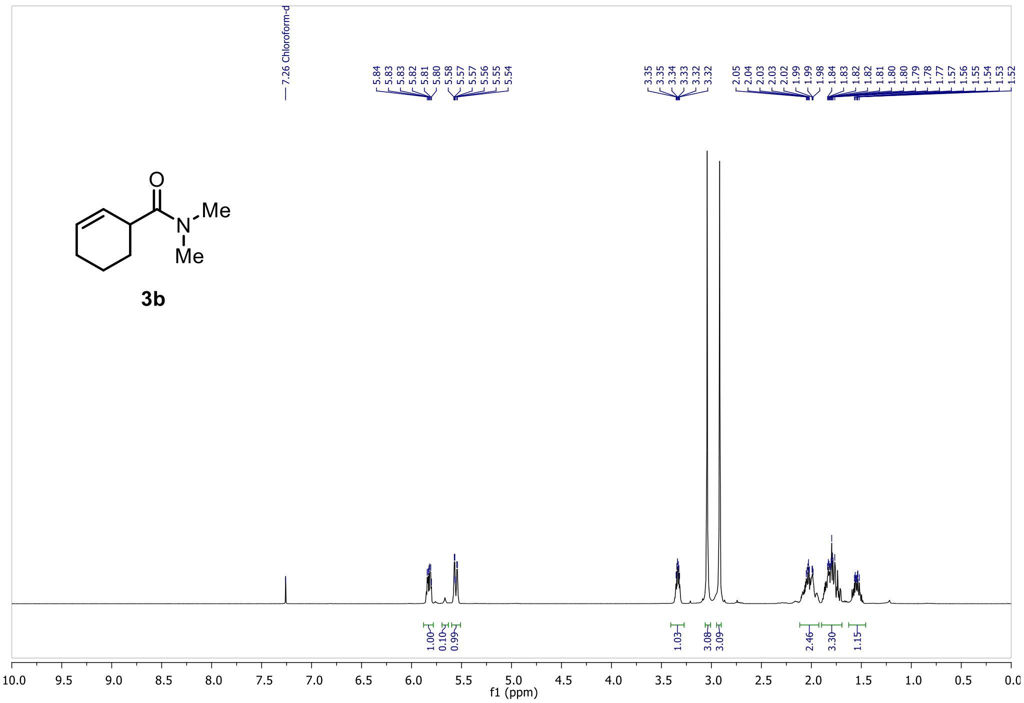

$^{13}C\{^{1}H\}$ NMR (101 MHz, $CDCl_3$):

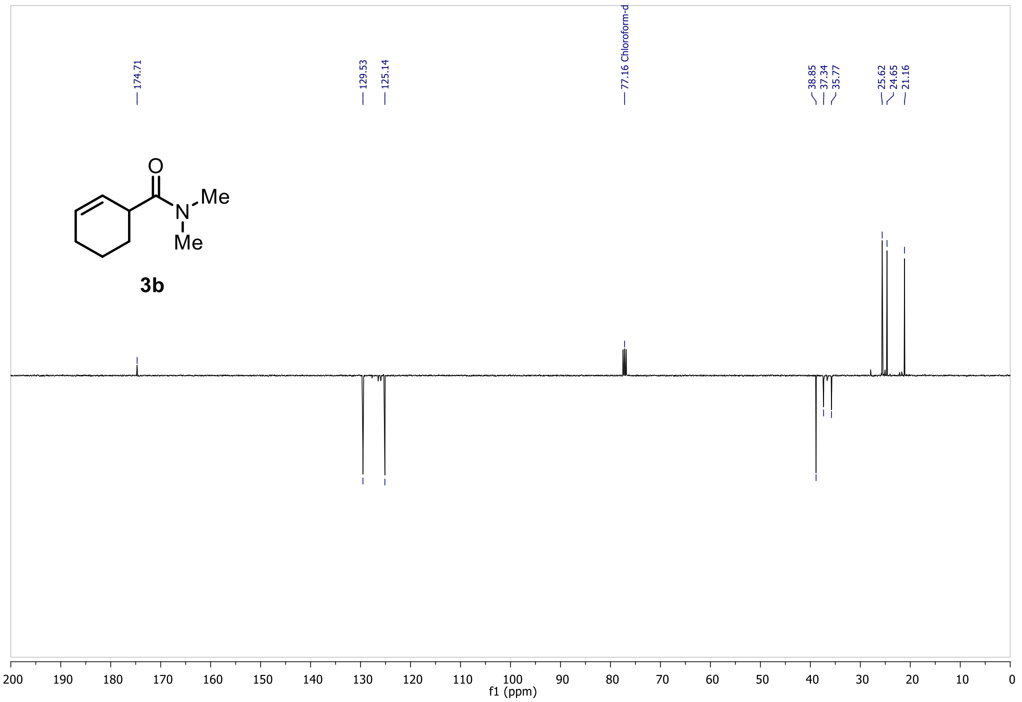

Cyclohex-2-en-1-yl(pyrrolidin-1-yl)methanone (3c)

^{1}H NMR (600 MHz, $CDCl_3$):

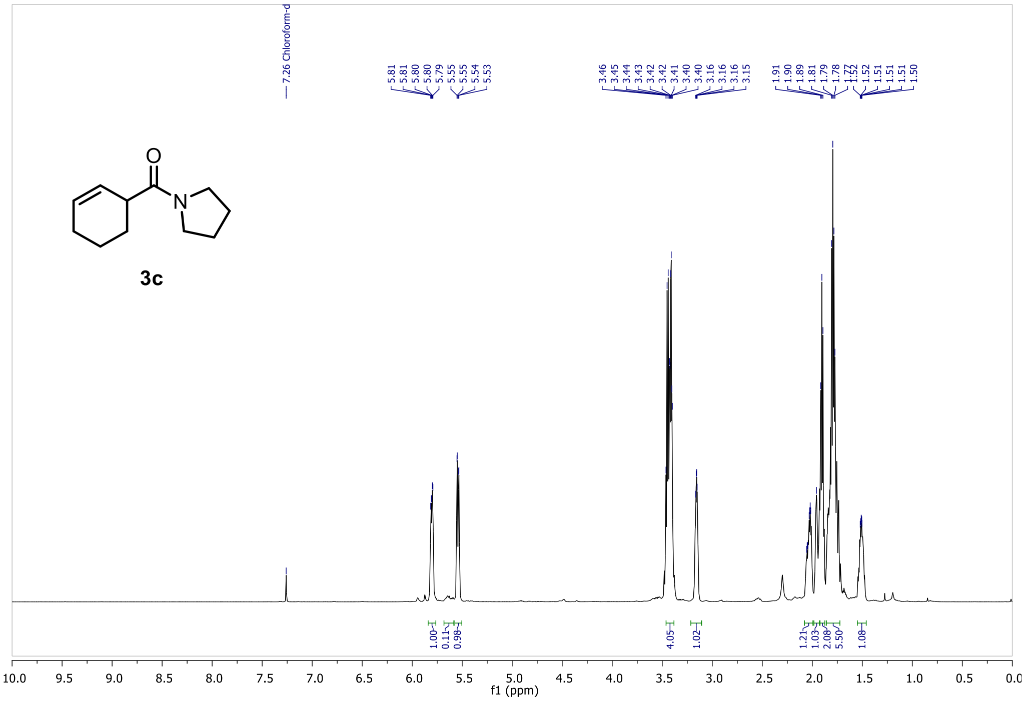

$^{13}C\{^1H\}$ NMR (151 MHz, $CDCl_3$):

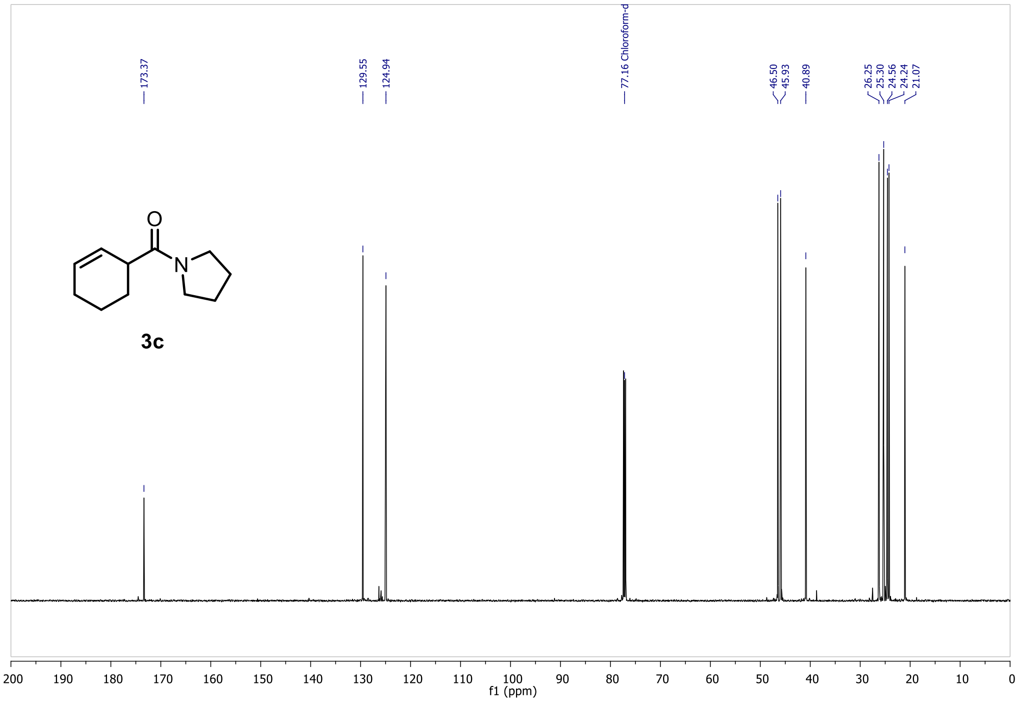

Cyclohex-2-en-1-yl(3,4-dihydroisoquinolin-2(1*H*)-yl)methanone (3d)

^{1}H NMR (600 MHz, $CDCl_3$):

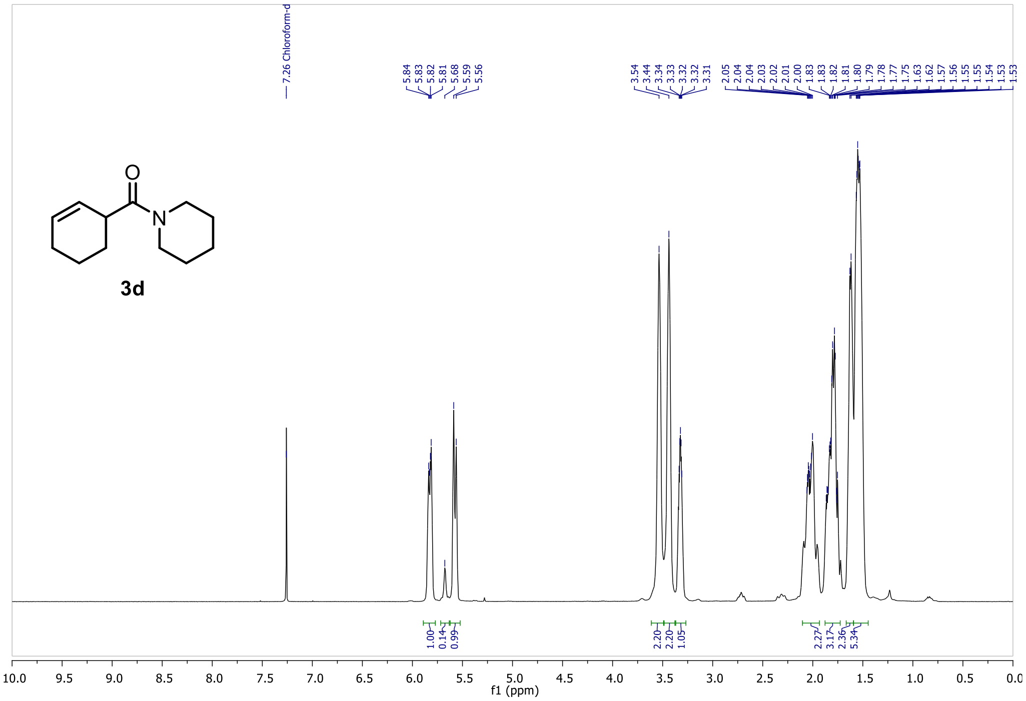

^{13}C{^{1}H} NMR (151 MHz, $CDCl_3$):

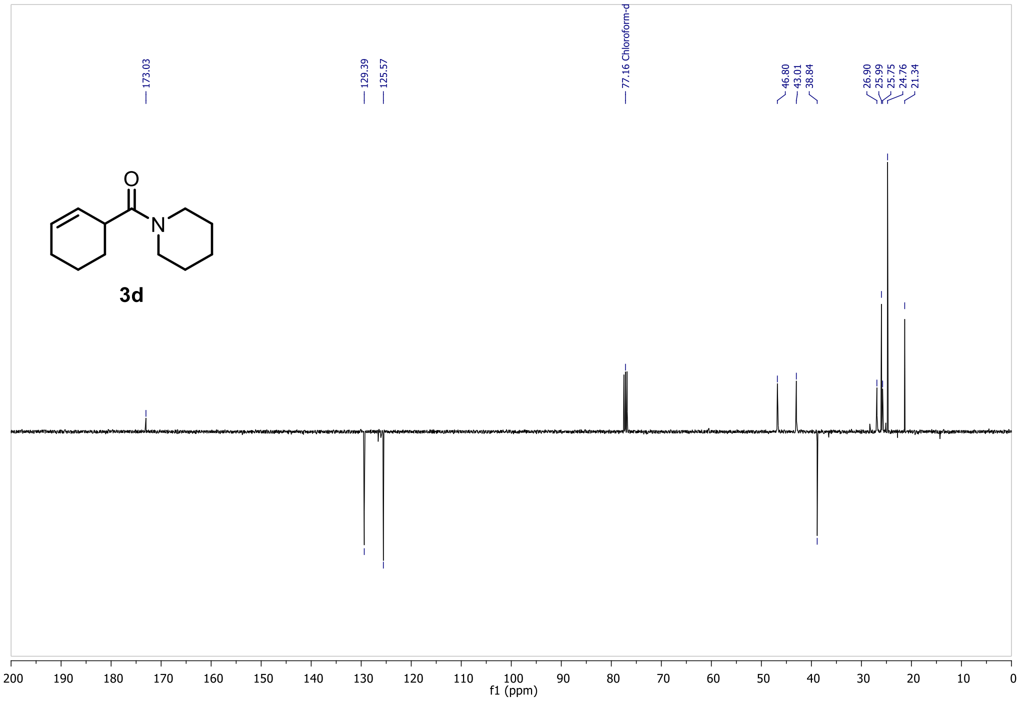

N,N-Diisopropylcyclohex-2-ene-1-carboxamide (3e)

^{1}H NMR (400 MHz, $CDCl_3$):

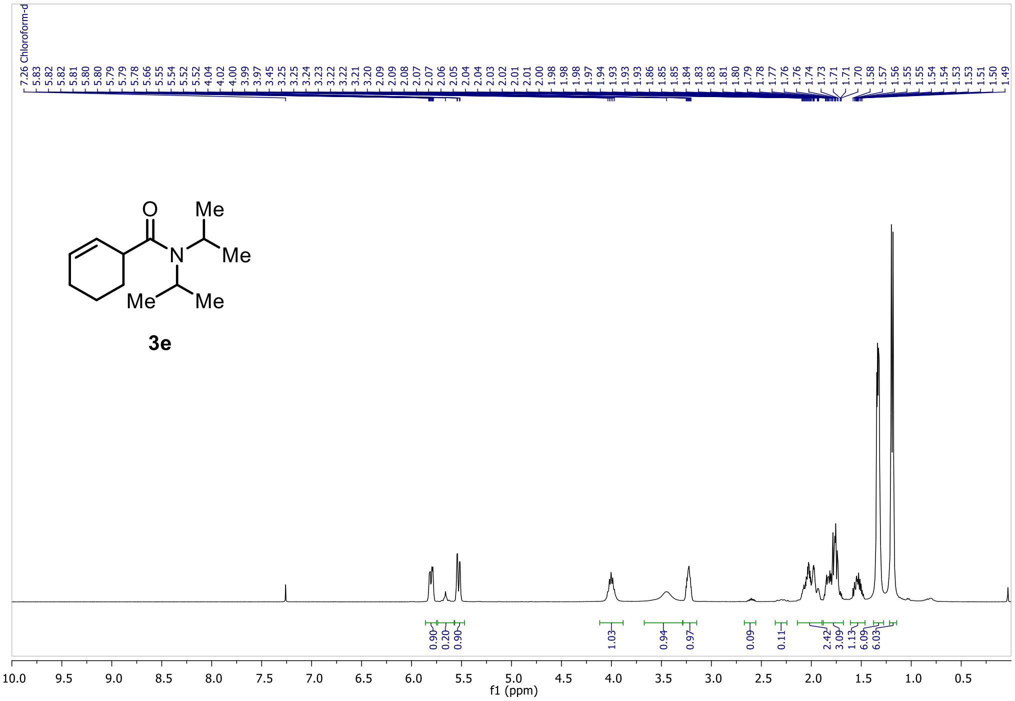

$^{13}C\{^{1}H\}$ NMR (101 MHz, $CDCl_3$):

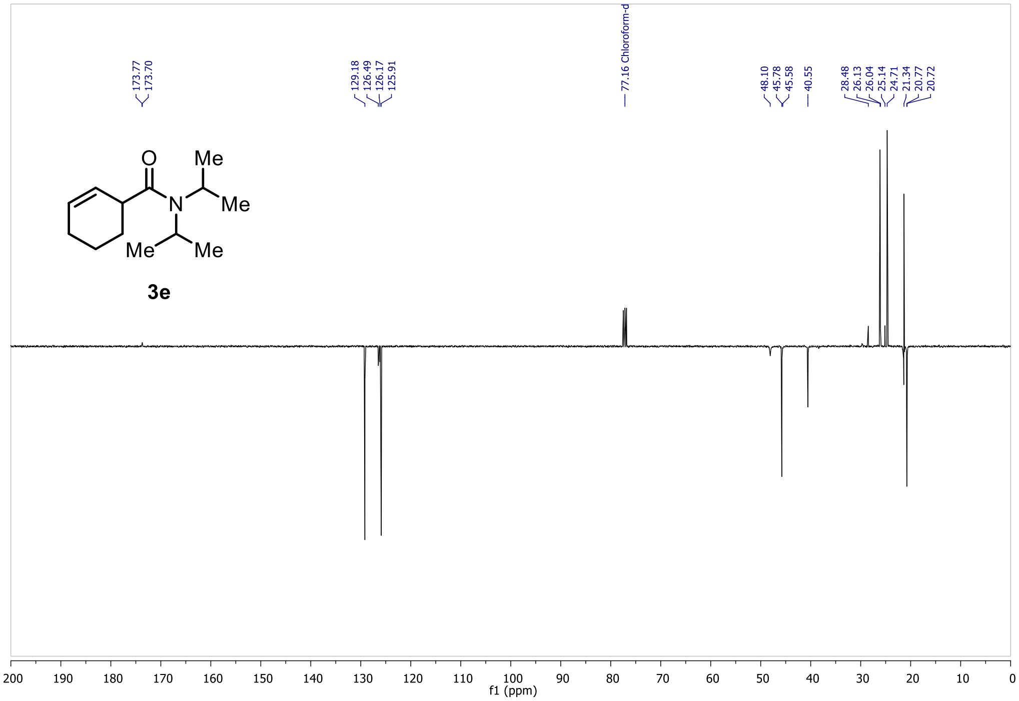

Cyclohex-2-en-1-yl(3,4-dihydroisoquinolin-2(1*H*)-yl)methanone (3f)

^{1}H NMR (600 MHz, CDCl$_3$) – *Rotameric effects increase the number of peaks*:

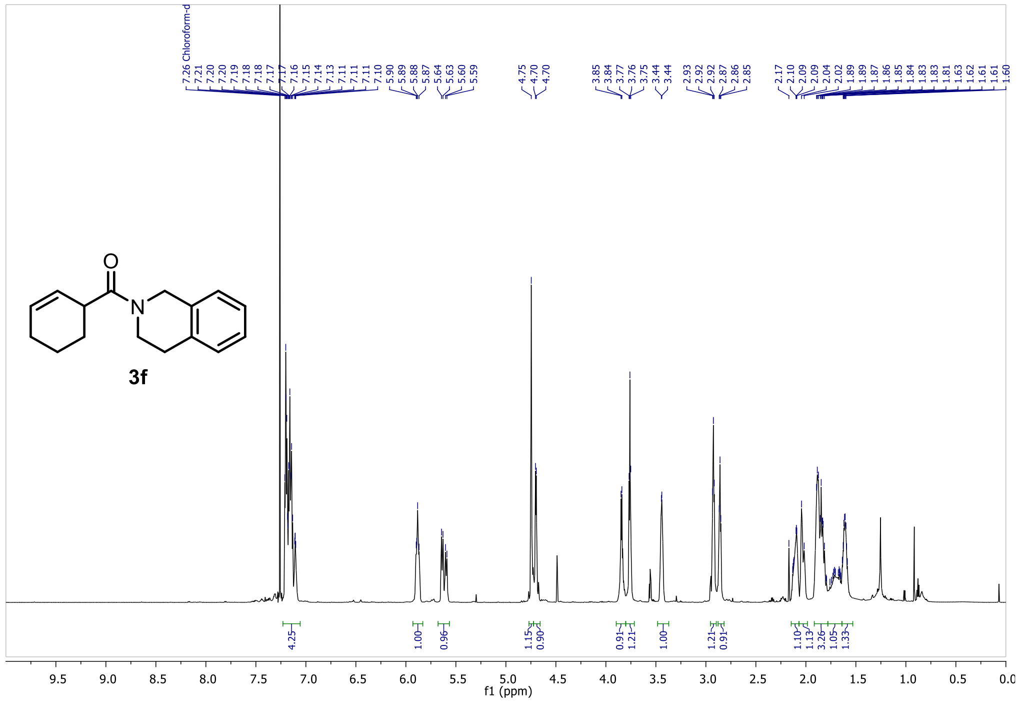

^{13}C{^{1}H} NMR (151 MHz, CDCl$_3$) – *Rotameric effects increase the number of peaks*:

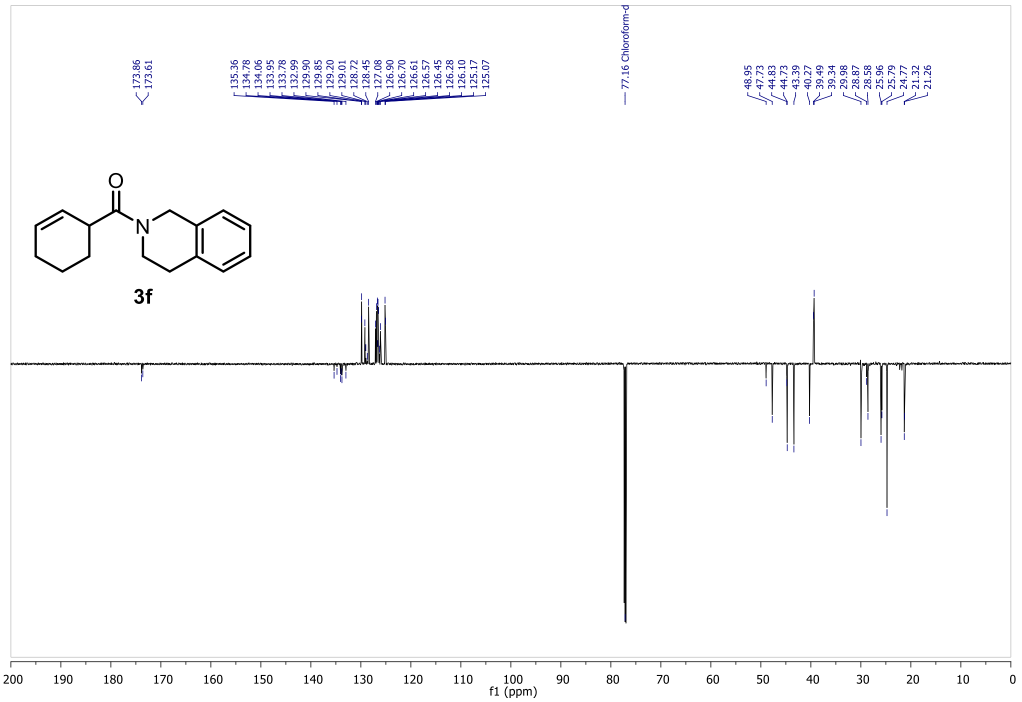

Cyclohex-3-en-1-yl(3,4-dihydroisoquinolin-2(1*H*)-yl)methanone (3f')

^{1}H NMR (600 MHz, CDCl$_3$) – *Rotameric effects increase the number of peaks*:

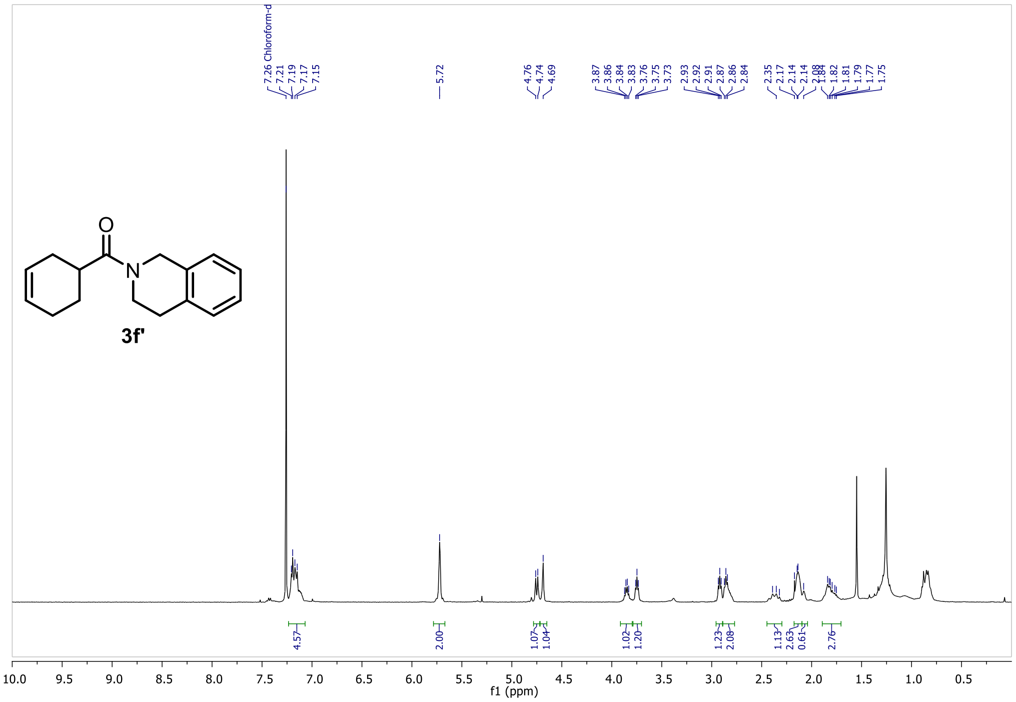

^{13}C{^{1}H} NMR (151 MHz, CDCl$_3$) – *Rotameric effects increase the number of peaks*:

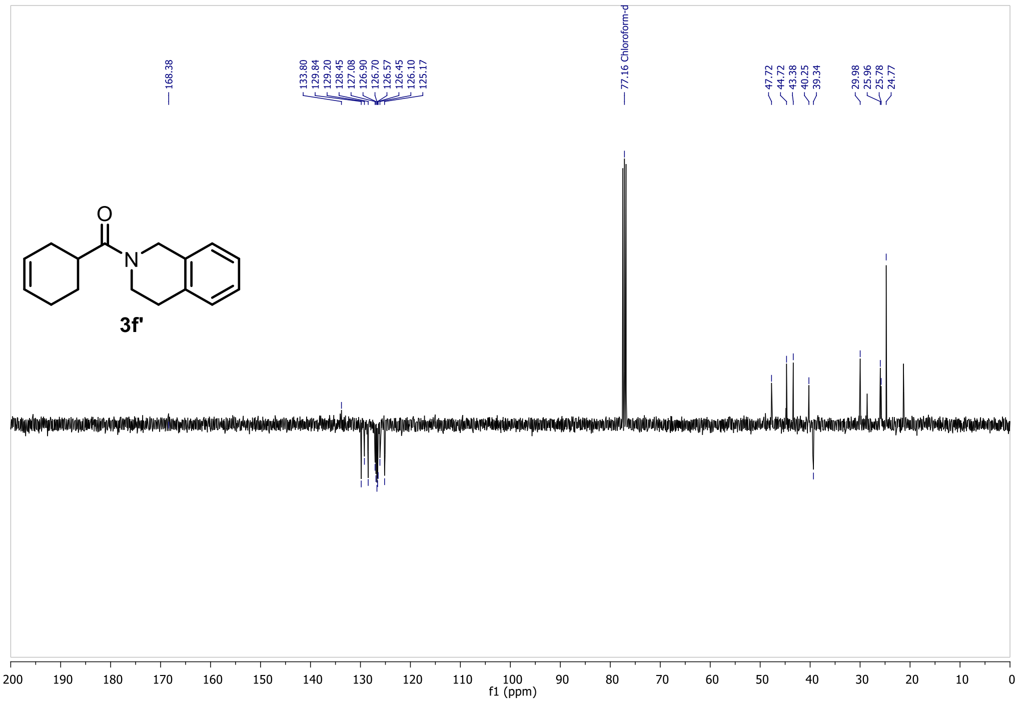

Cyclohex-2-en-1-yl(morpholino)methanone (3g)

^{1}H NMR (600 MHz, CDCl$_3$):

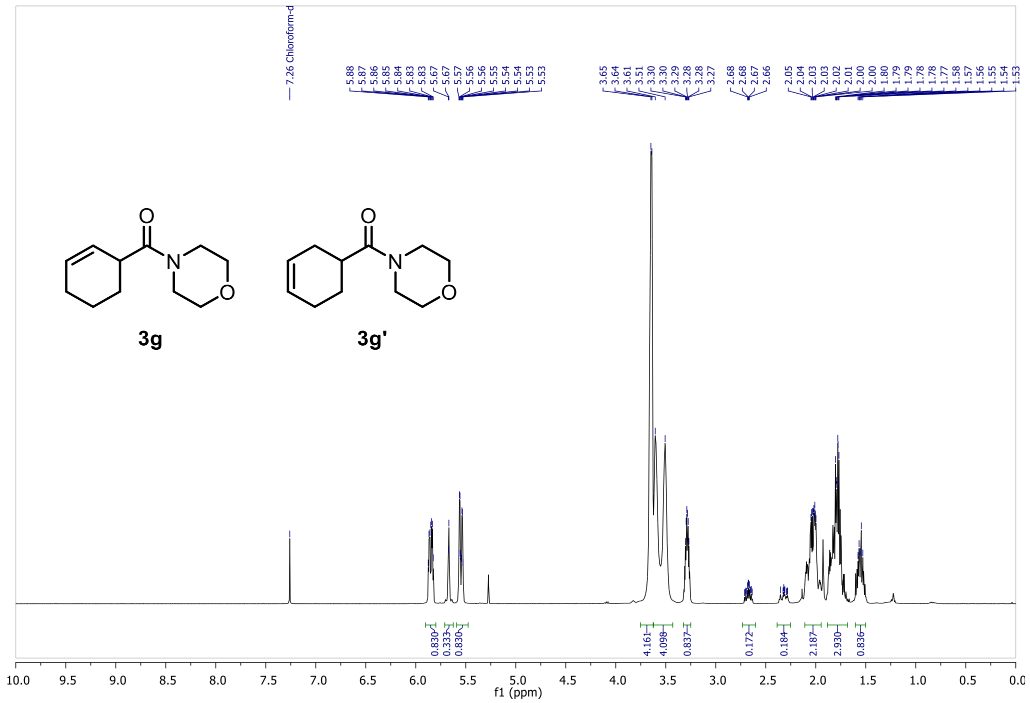

^{13}C{^{1}H} NMR (151 MHz, CDCl$_3$):

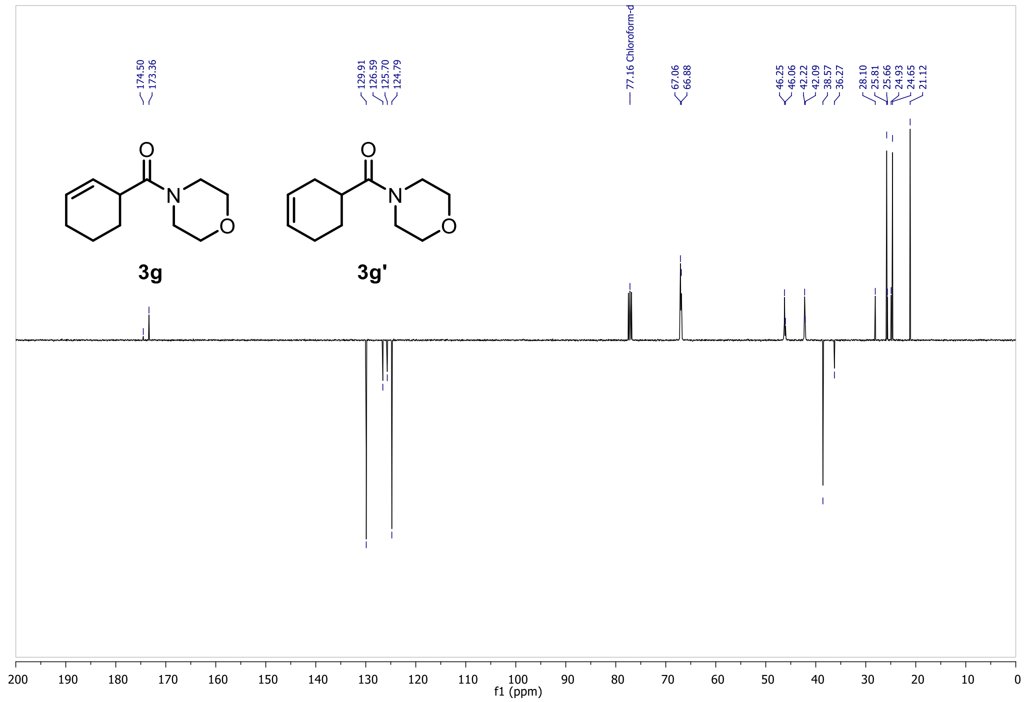

***N*-Methyl-*N*-phenylcyclohex-2-ene-1-carboxamide (3h)**

^{1}H NMR (600 MHz, $CDCl_3$):

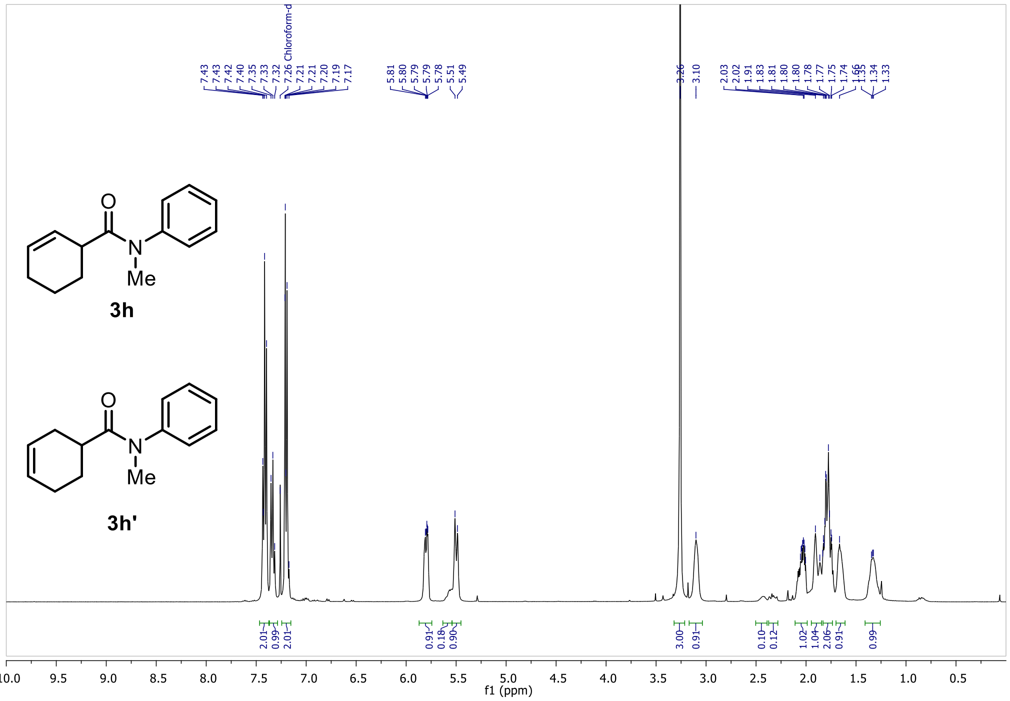

^{13}C{^{1}H} NMR (151 MHz, $CDCl_3$):

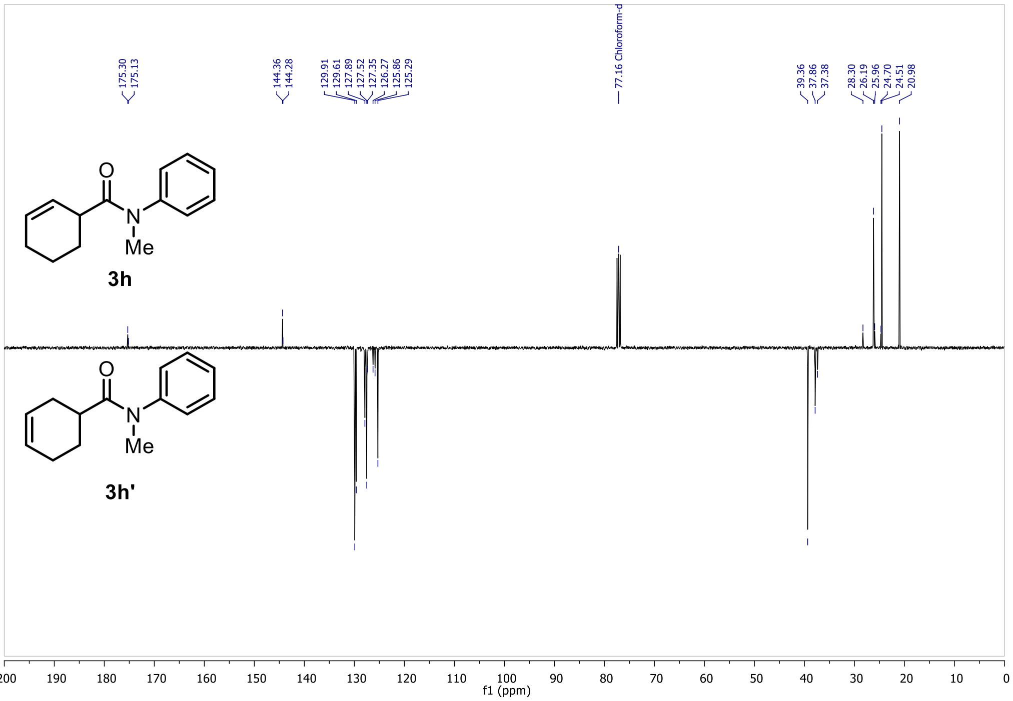

***N*,*N*-Diethyl-2,3,4,5-tetrahydro-[1,1'-biphenyl]-2-carboxamide (3i)**

^{1}H NMR (400 MHz, $CDCl_3$):

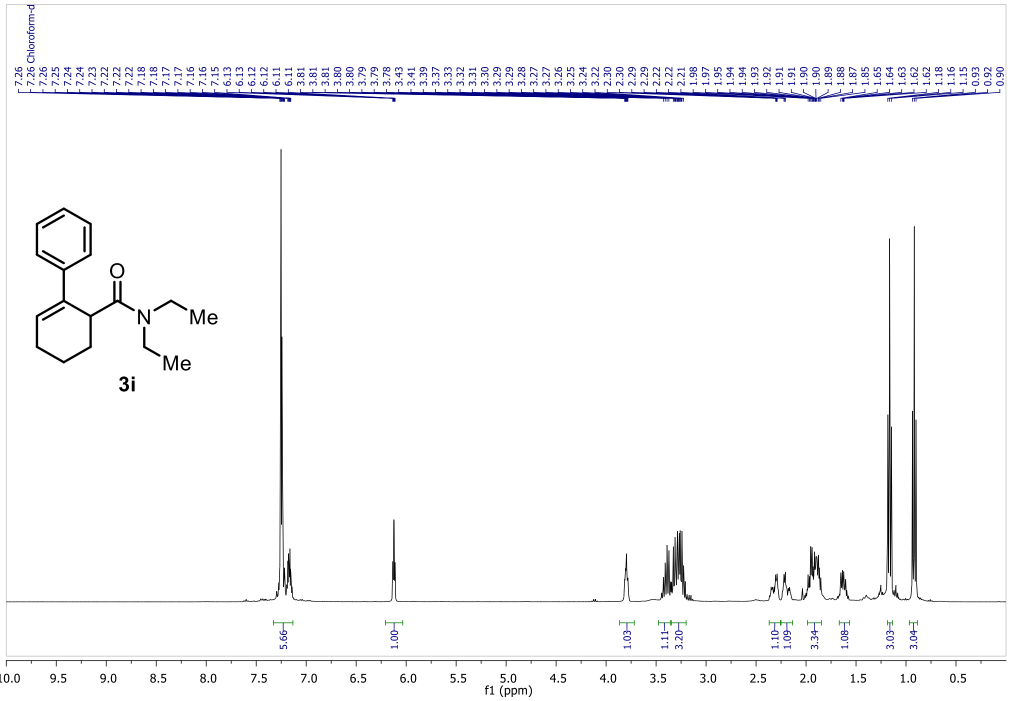

$^{13}C\{^{1}H\}$ NMR (101 MHz, $CDCl_3$):

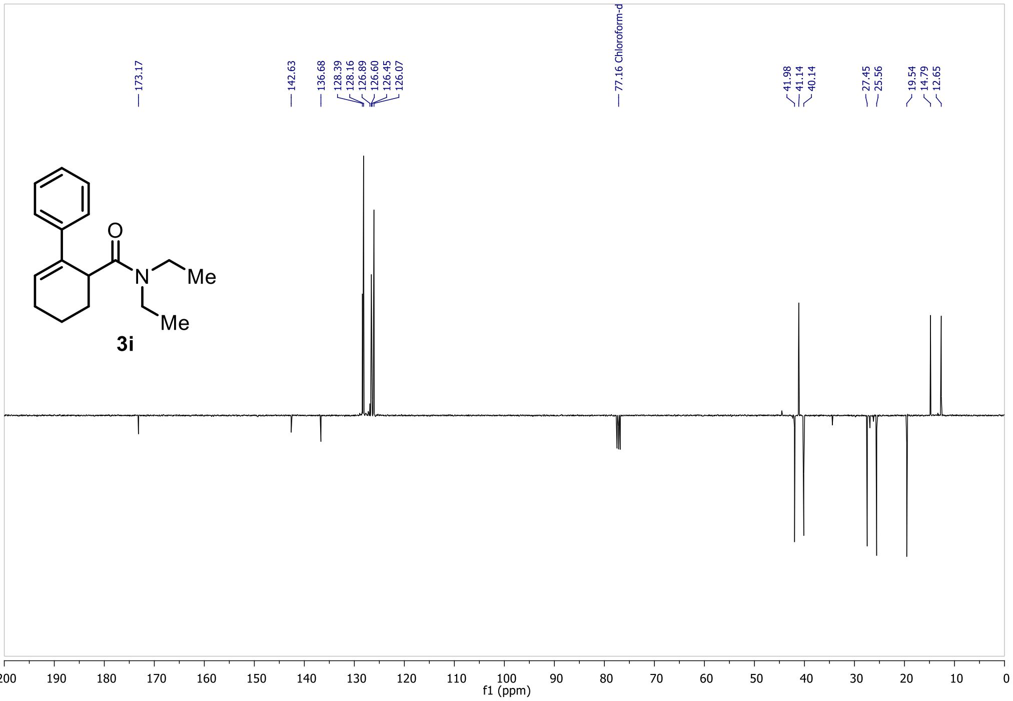

N,N-2-Trimethylcyclohex-2-ene-1-carboxamide (3j)

^{1}H NMR (600 MHz, $CDCl_3$):

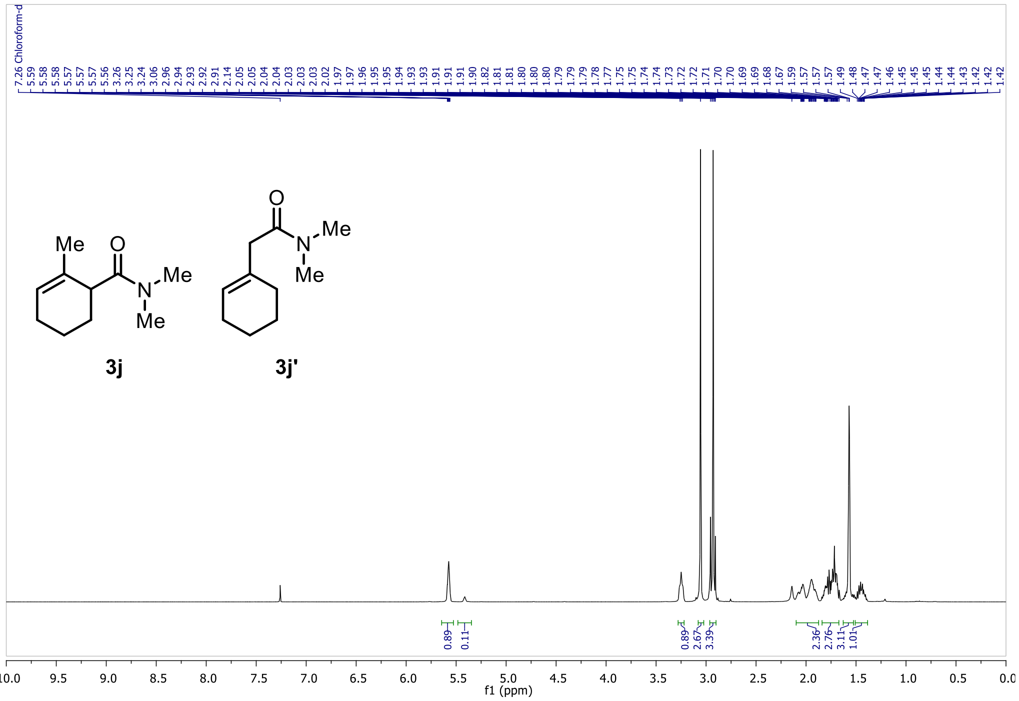

^{13}C{^{1}H} NMR (151 MHz, $CDCl_3$):

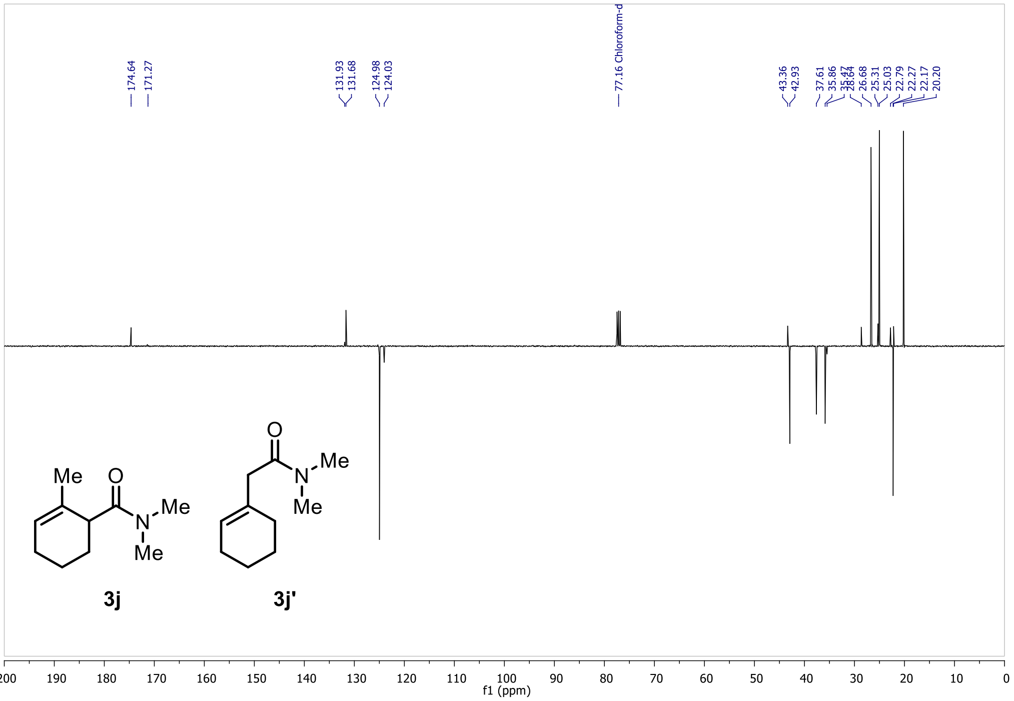

***N*,*N*-Diethylcyclopent-2-ene-1-carboxamide (3k)**

^{1}H NMR (400 MHz, $CDCl_3$):

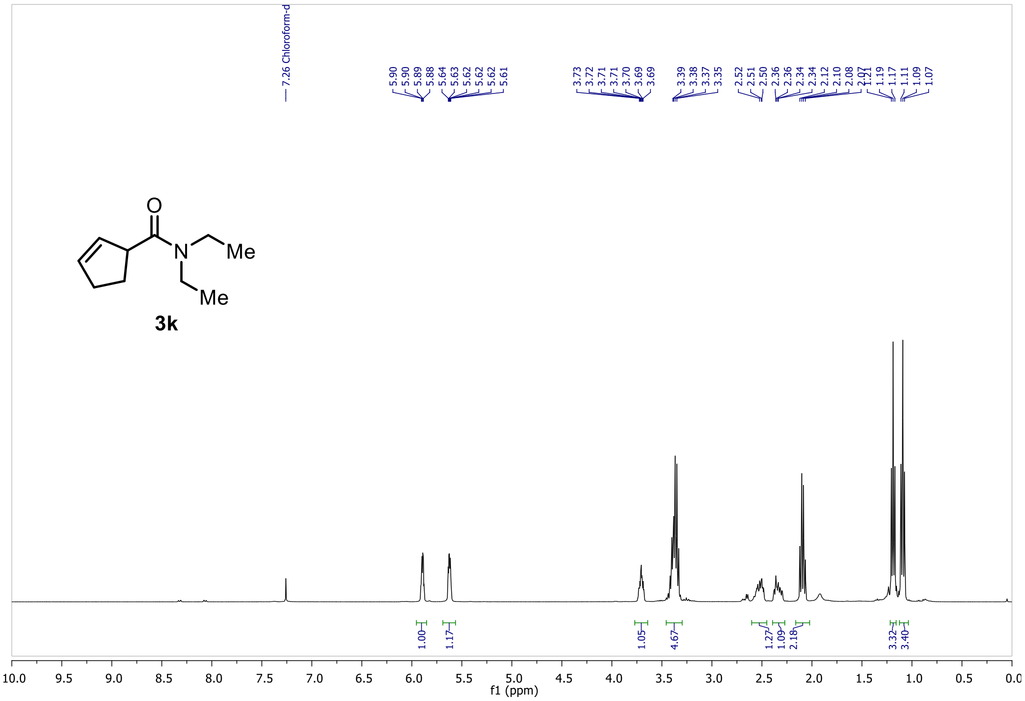

^{13}C{^{1}H} NMR (101 MHz, $CDCl_3$):

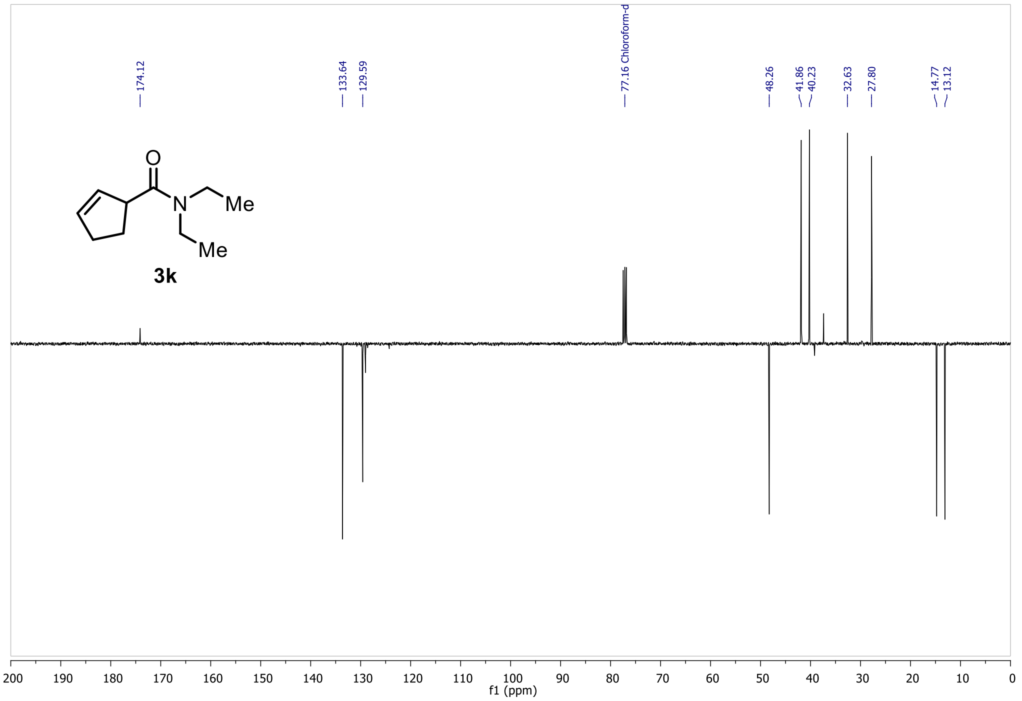

***N*,*N*-Diethylcyclopent-3-ene-1-carboxamide (3k')**

^{1}H NMR (400 MHz, $CDCl_3$):

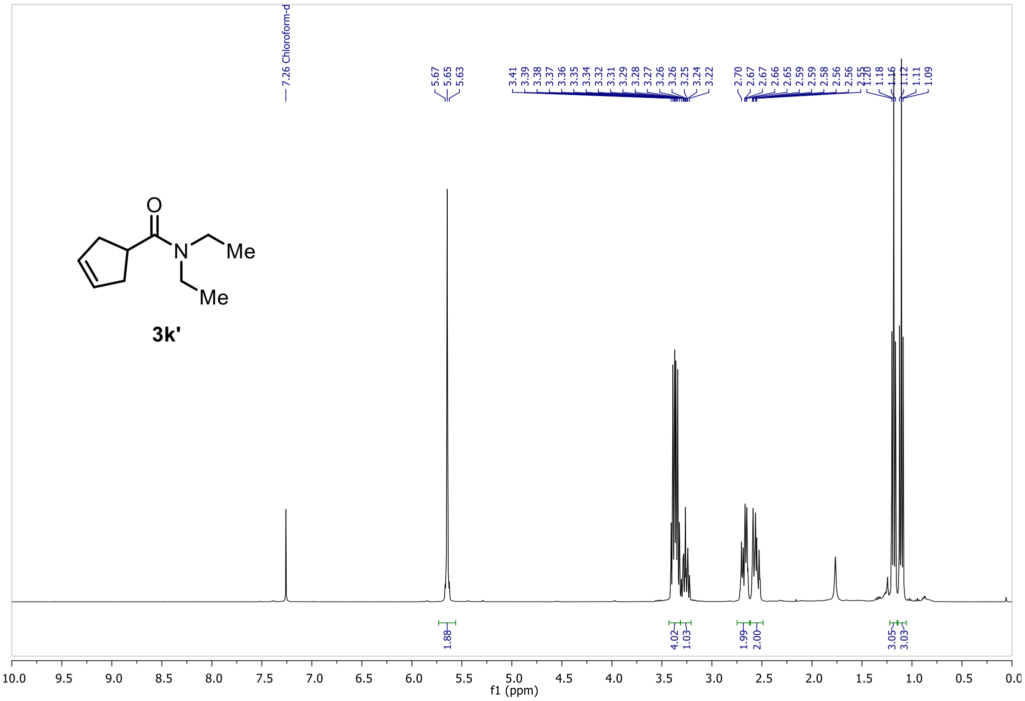

$^{13}C\{^{1}H\}$ NMR (101 MHz, $CDCl_3$):

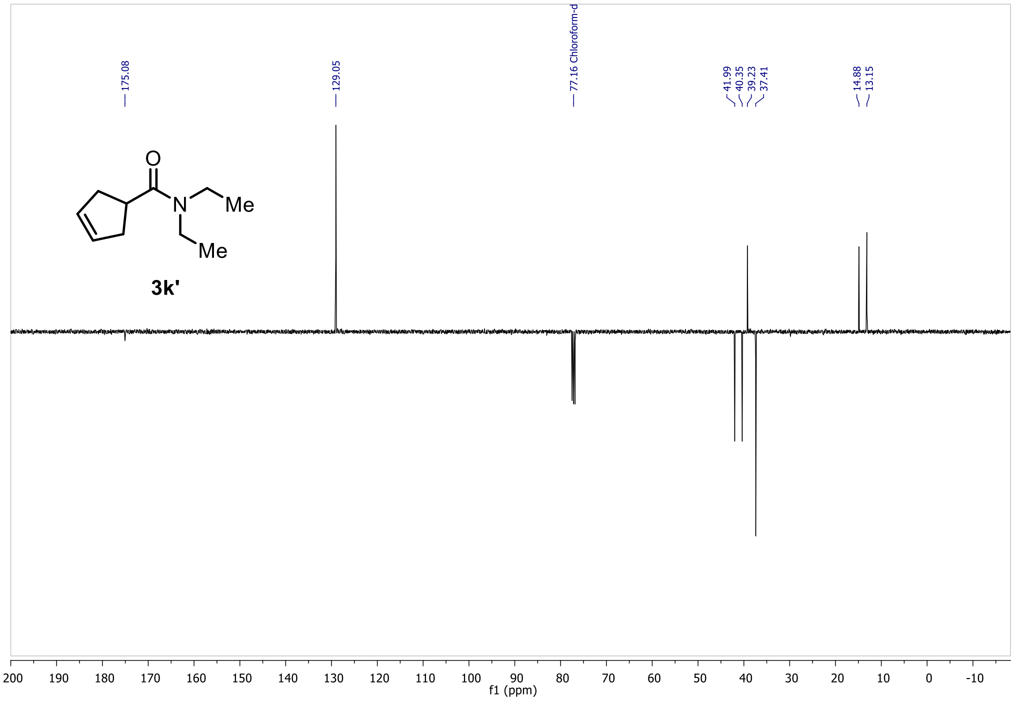

***N*,*N*-Dimethylcyclopent-2-ene-1-carboxamide (3l)**

^{1}H NMR (400 MHz, CDCl$_3$):

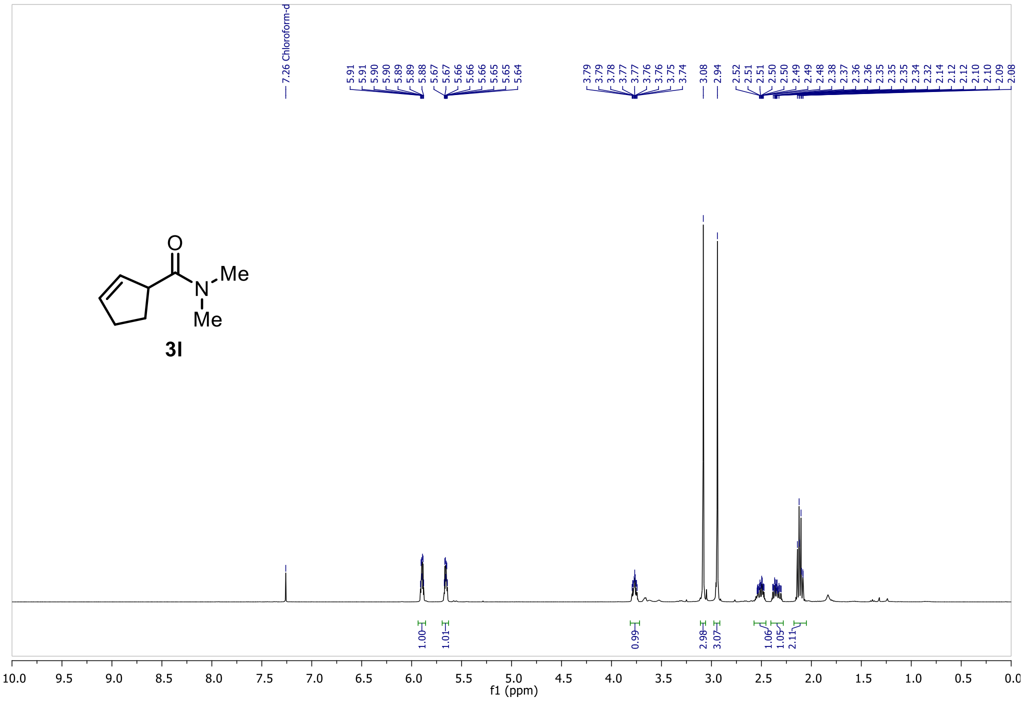

^{13}C{^{1}H} NMR (101 MHz, CDCl$_3$):

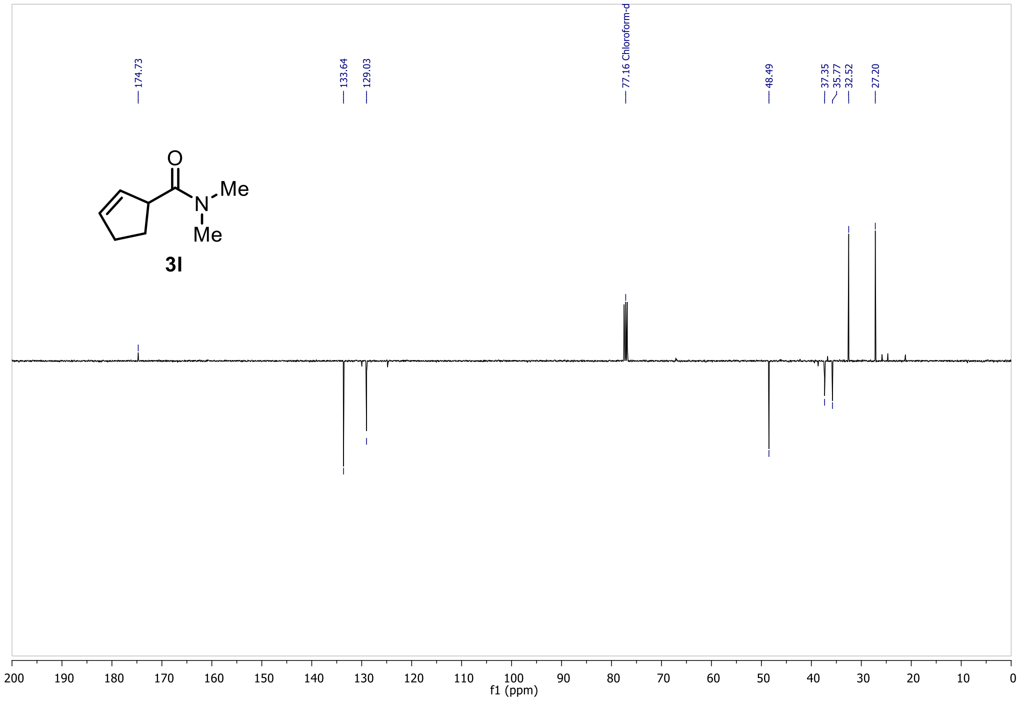

N,*N*-Diethylcyclopent-3-ene-1-carboxamide (3l’)

^{1}H NMR (400 MHz, CDCl$_3$):

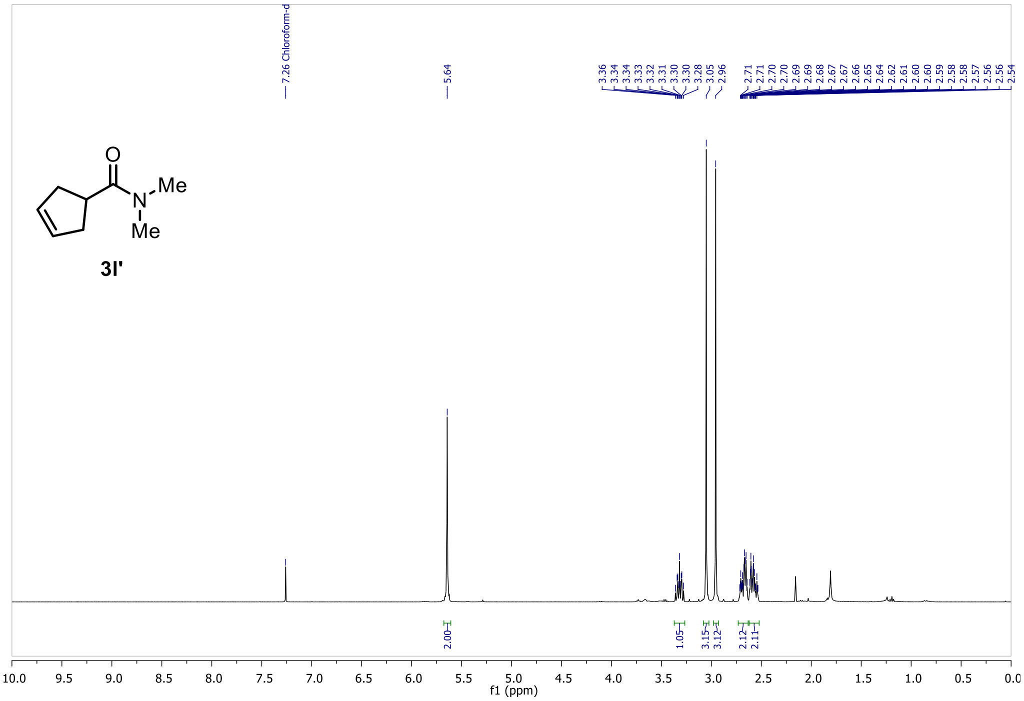

^{13}C{^{1}H} NMR (101 MHz, CDCl$_3$):

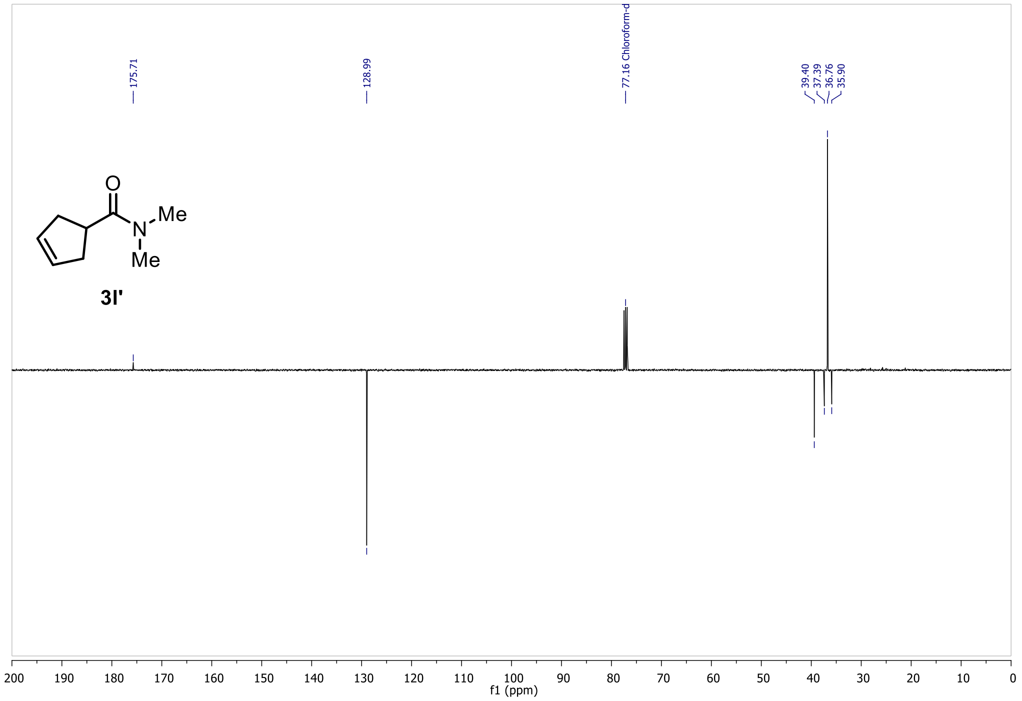

***N*,*N*-Diisopropylcyclopent-2-ene-1-carboxamide (3m)**

^{1}H NMR (400 MHz, $CDCl_3$):

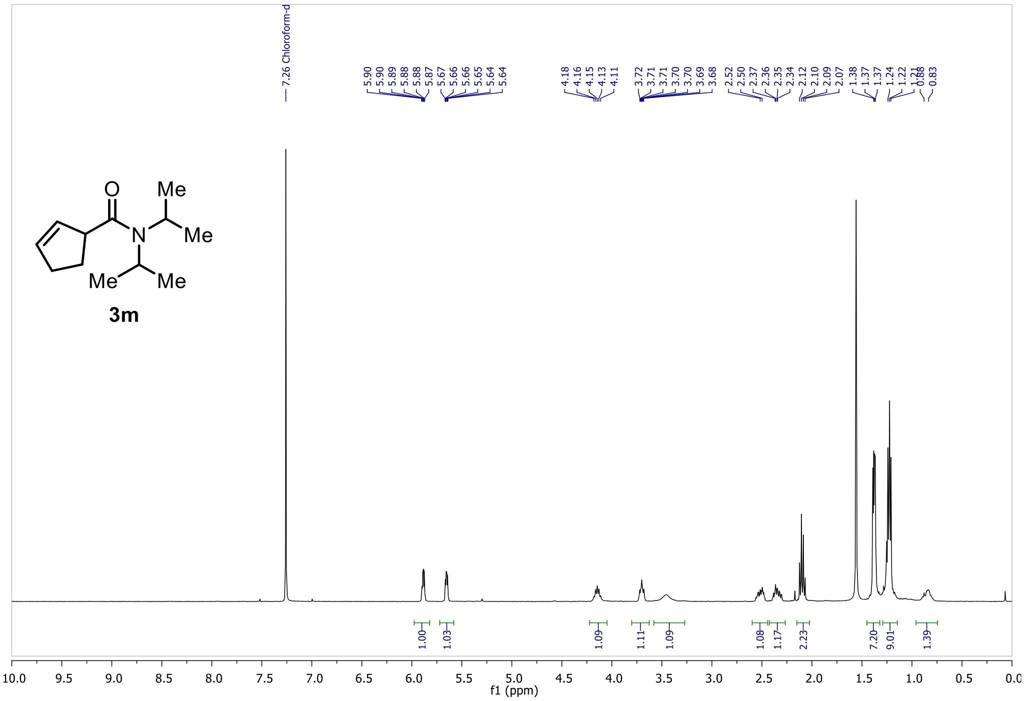

^{13}C{^{1}H} NMR (101 MHz, $CDCl_3$):

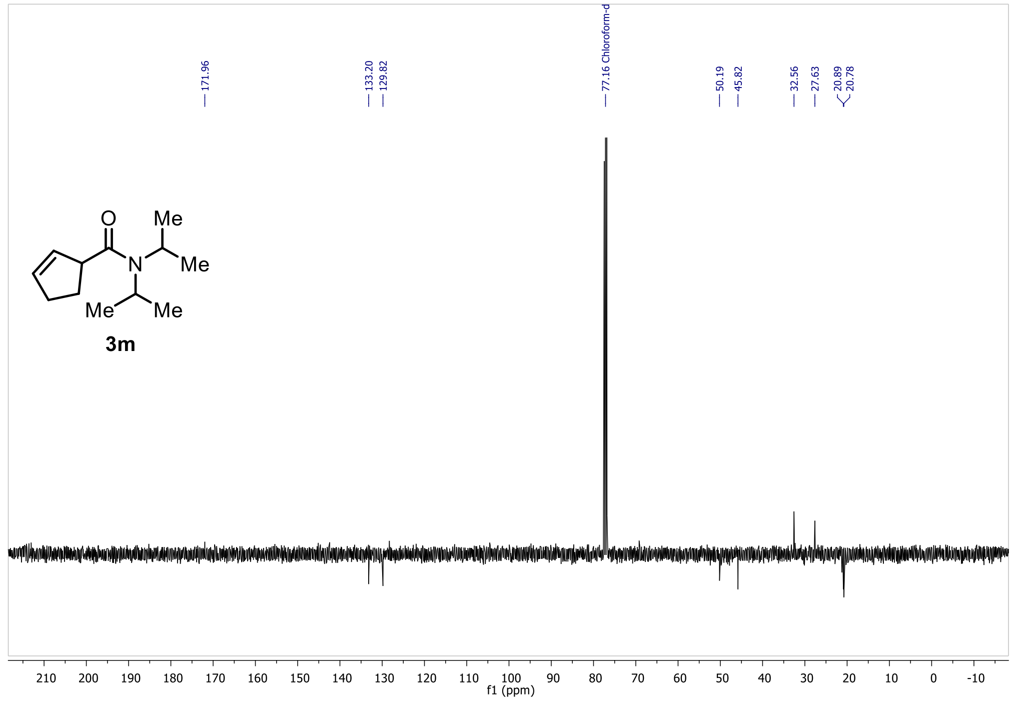

***N*,*N*-Diisopropylcyclopent-3-ene-1-carboxamide (3m')**

^{1}H NMR (400 MHz, CDCl$_3$):

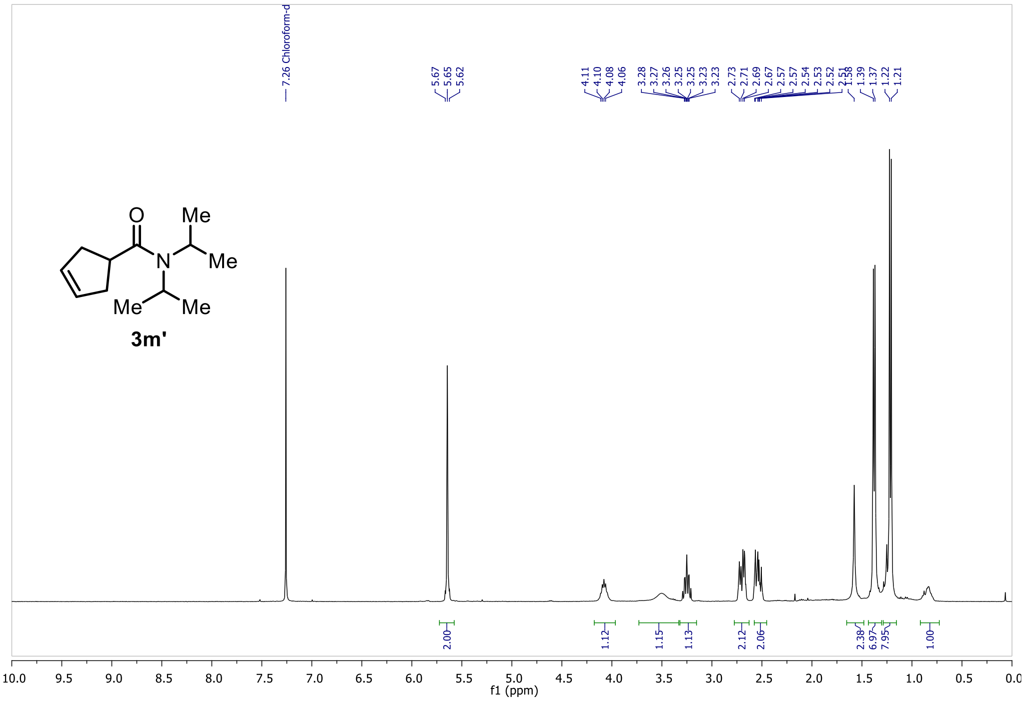

^{13}C{^{1}H} NMR (101 MHz, CDCl$_3$):

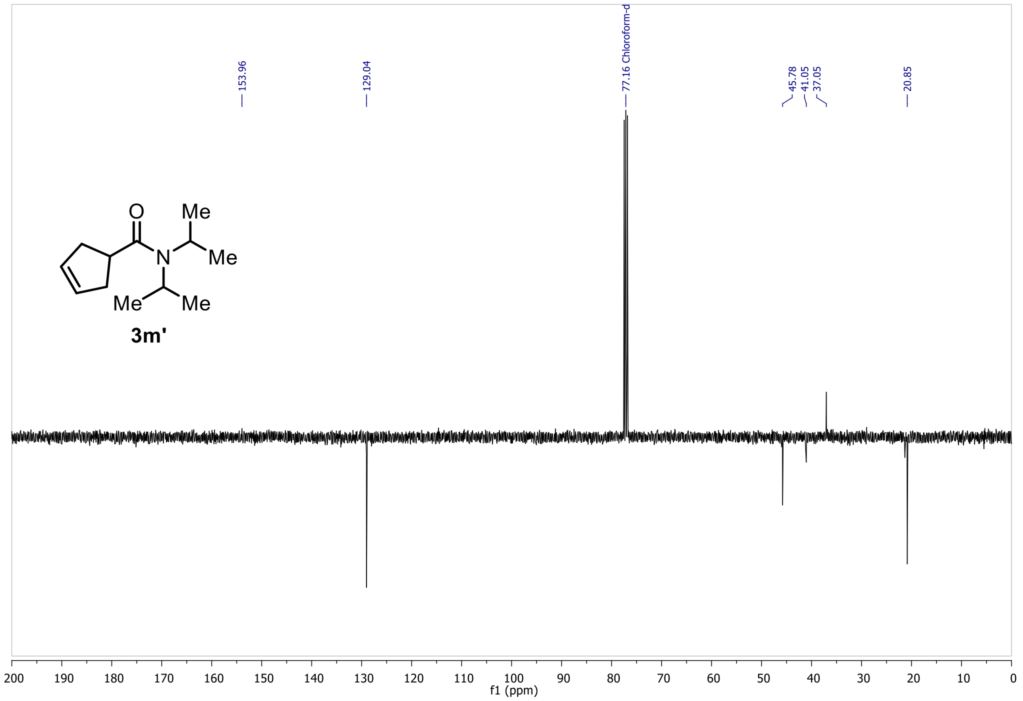

Cyclopent-2-en-1-yl(piperidin-1-yl)methanone (3n)

^{1}H NMR (400 MHz, $CDCl_3$):

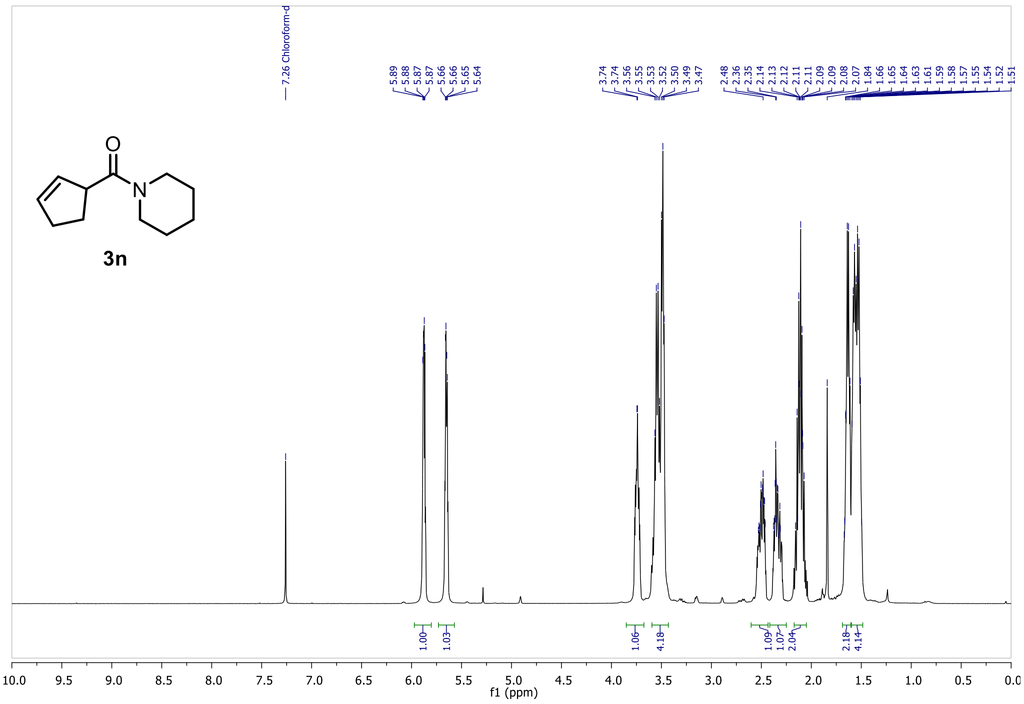

^{13}C{^{1}H} NMR (101 MHz, $CDCl_3$):

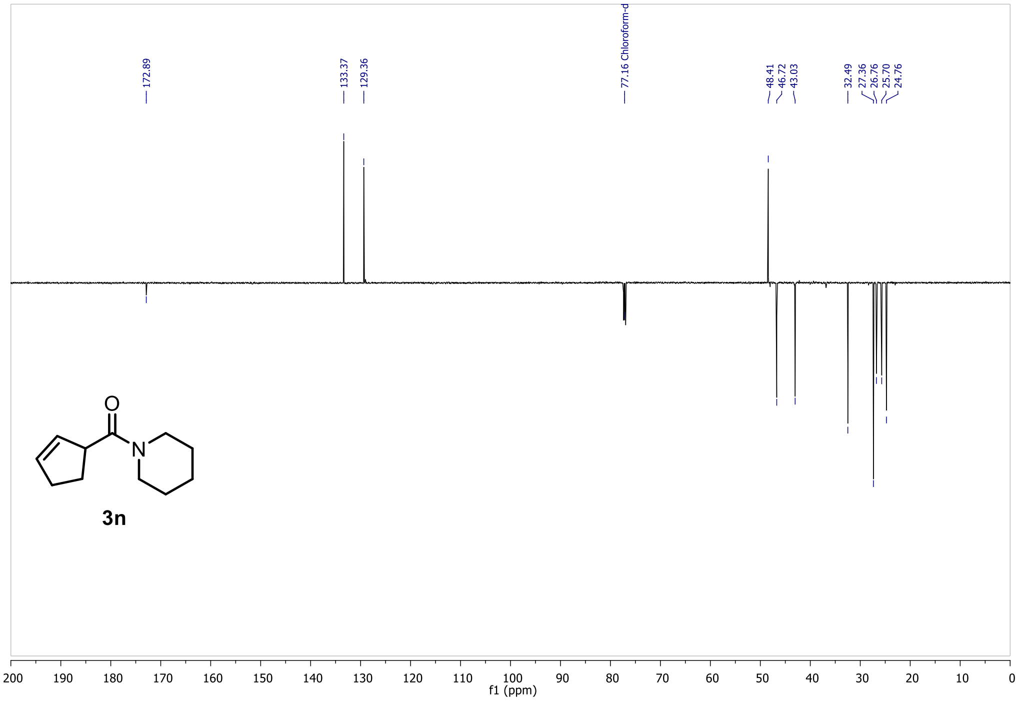

Cyclopent-3-en-1-yl(piperidin-1-yl)methanone (3n')

^{1}H NMR (400 MHz, $CDCl_3$):

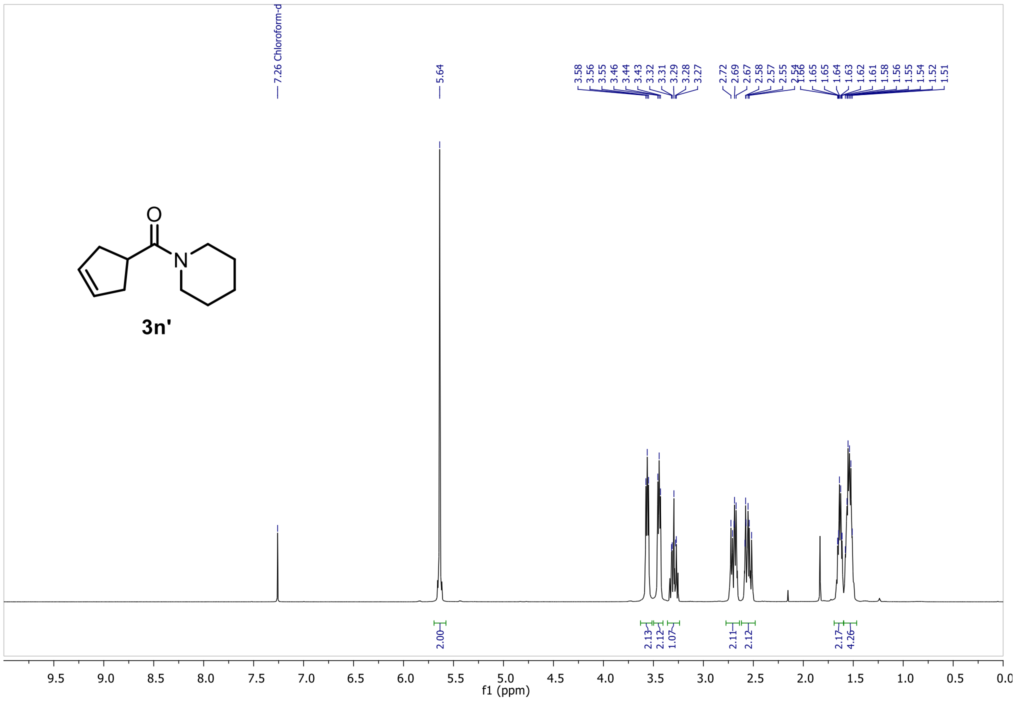

$^{13}C\{^1H\}$ NMR (101 MHz, $CDCl_3$):

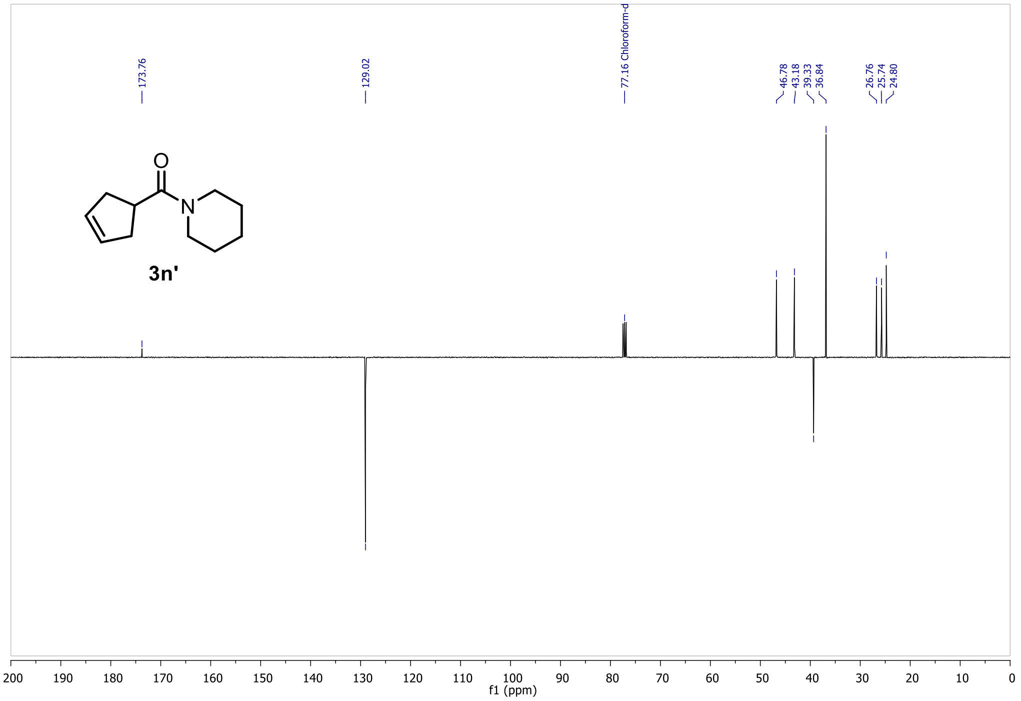

Cyclopent-2-en-1-yl(pyrrolidin-1-yl)methanone (3o)

^{1}H NMR (400 MHz, $CDCl_3$):

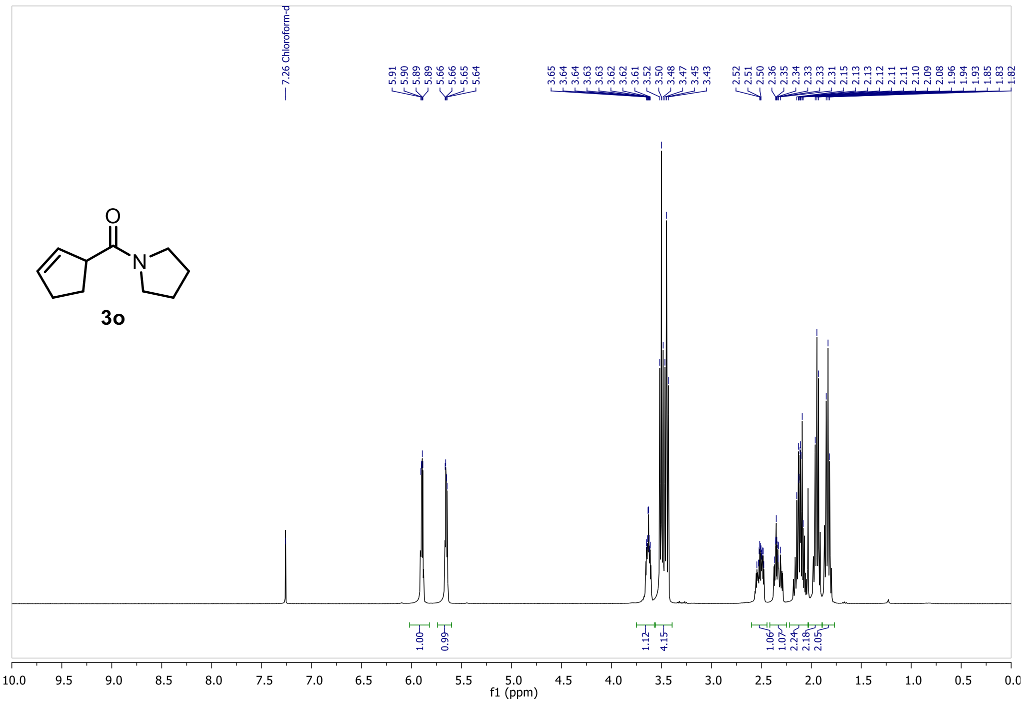

$^{13}C\{^{1}H\}$ NMR (101 MHz, $CDCl_3$):

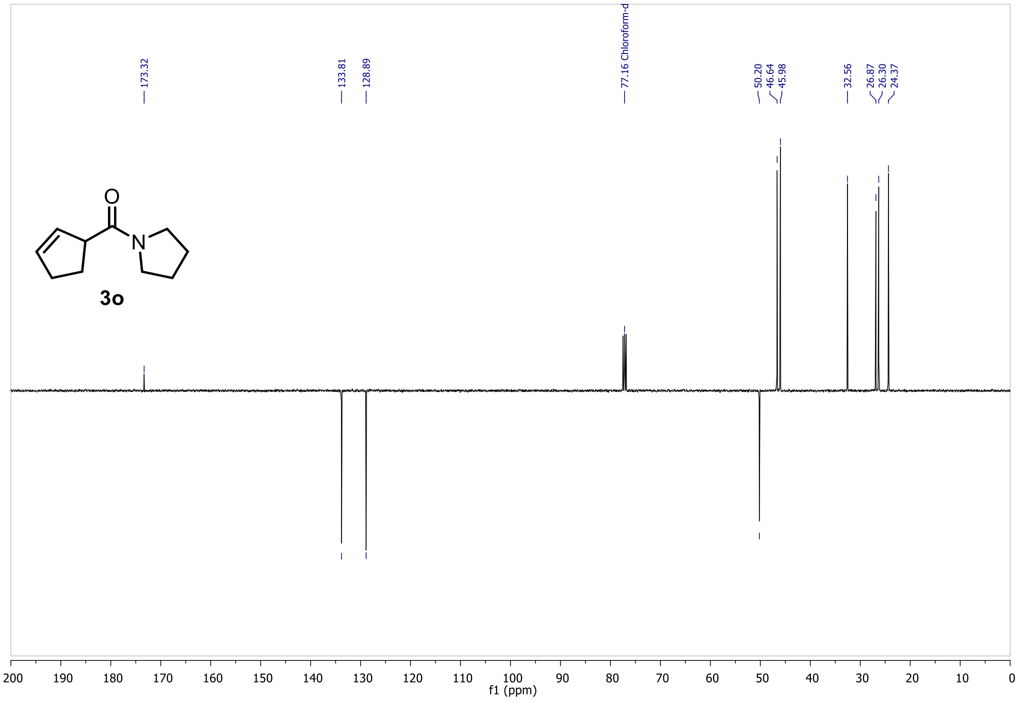

Cyclopent-3-en-1-yl(pyrrolidin-1-yl)methanone (3o’)

^{1}H NMR (400 MHz, $CDCl_3$):

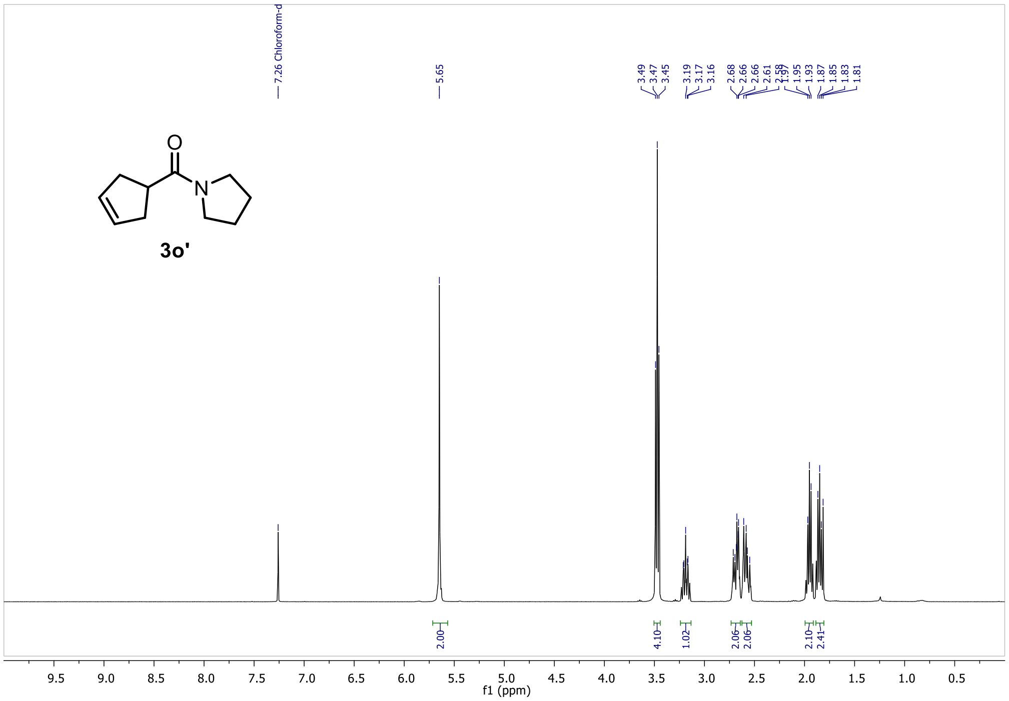

$^{13}C\{^{1}H\}$ NMR (101 MHz, $CDCl_3$):

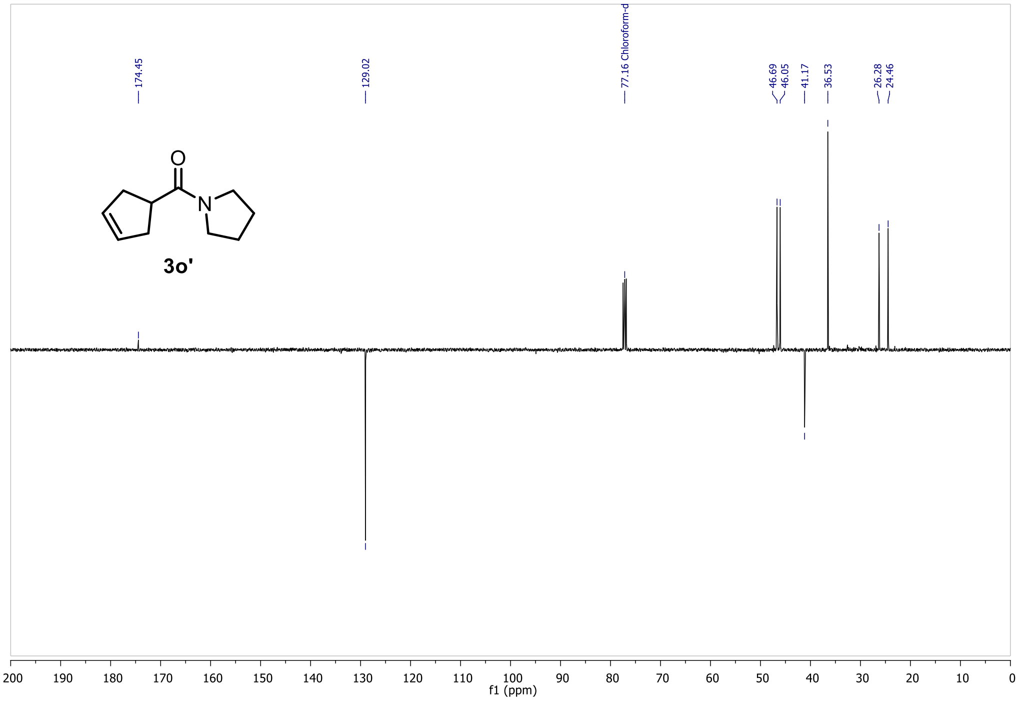

N,N-Diethyl-2-methylcyclopent-2-ene-1-carboxamide (3p)

^{1}H NMR (400 MHz, $CDCl_3$):

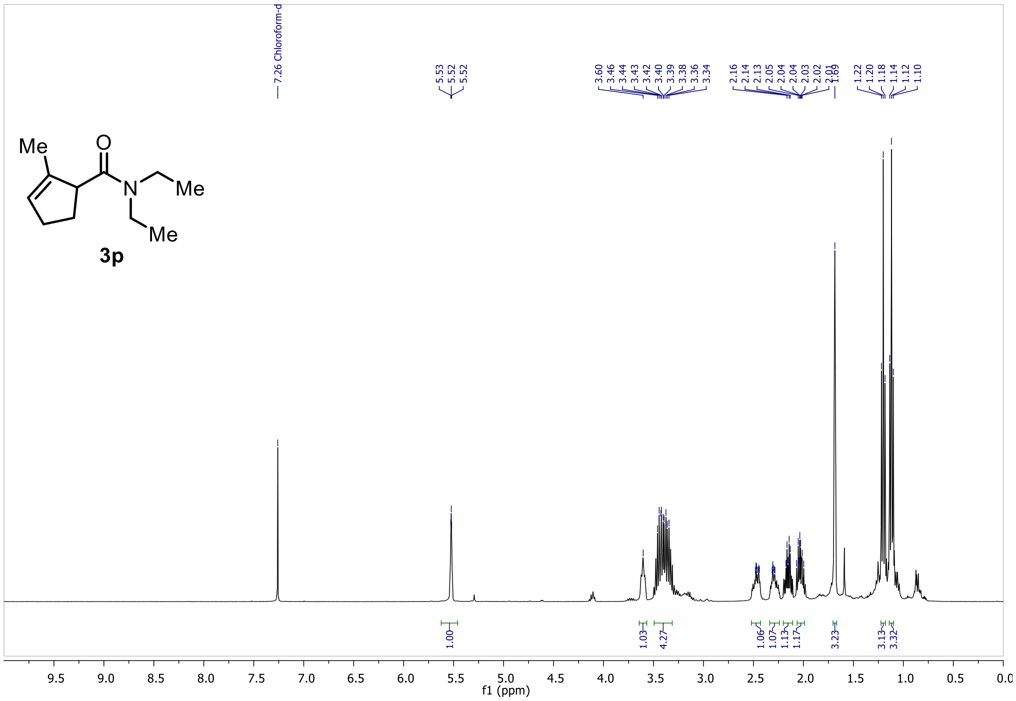

^{13}C{^{1}H} NMR (101 MHz, $CDCl_3$):

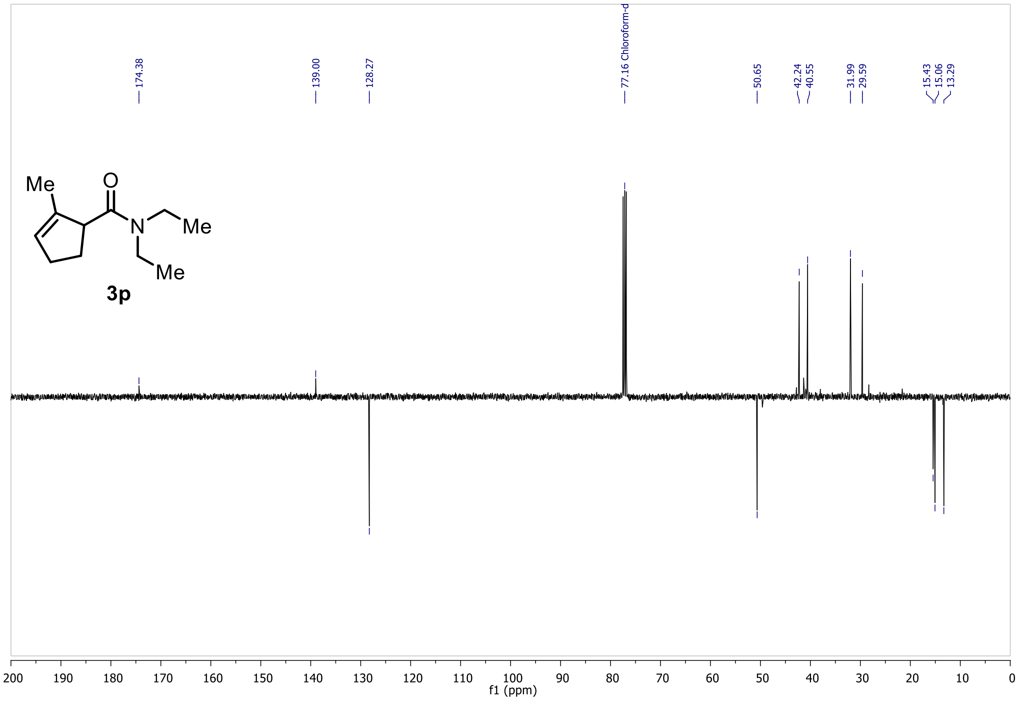

(*E*)-*N*,*N*-Diethylcyclododec-2-ene-1-carboxamide (3q)

^{1}H NMR (700 MHz, $CDCl_3$):

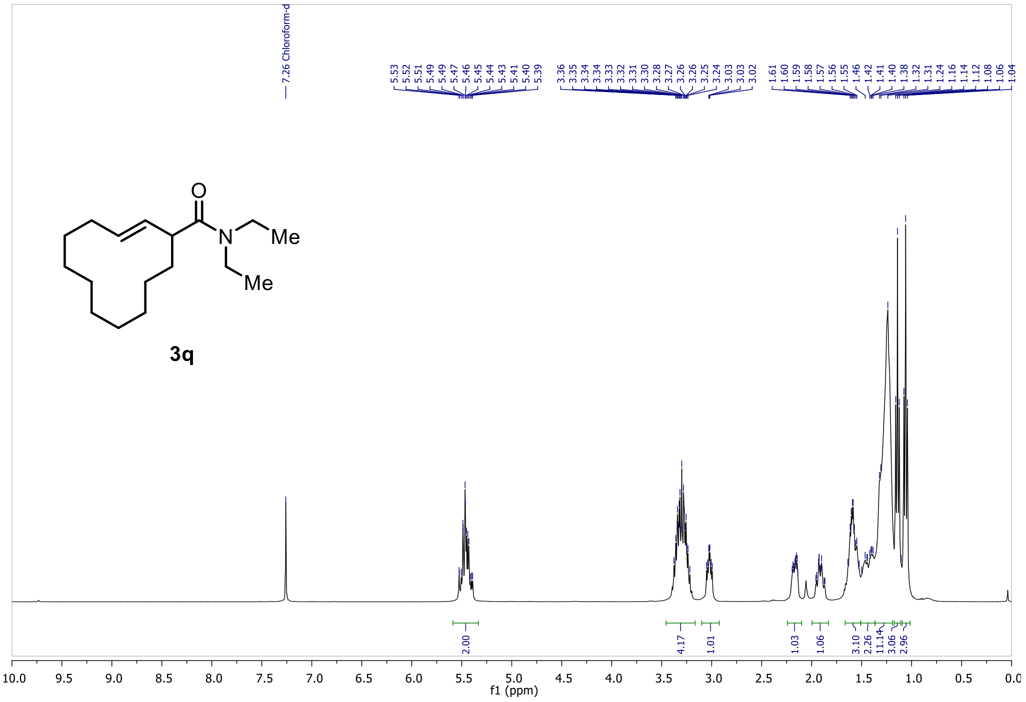

$^{13}C\{^1H\}$ NMR (151 MHz, $CDCl_3$):

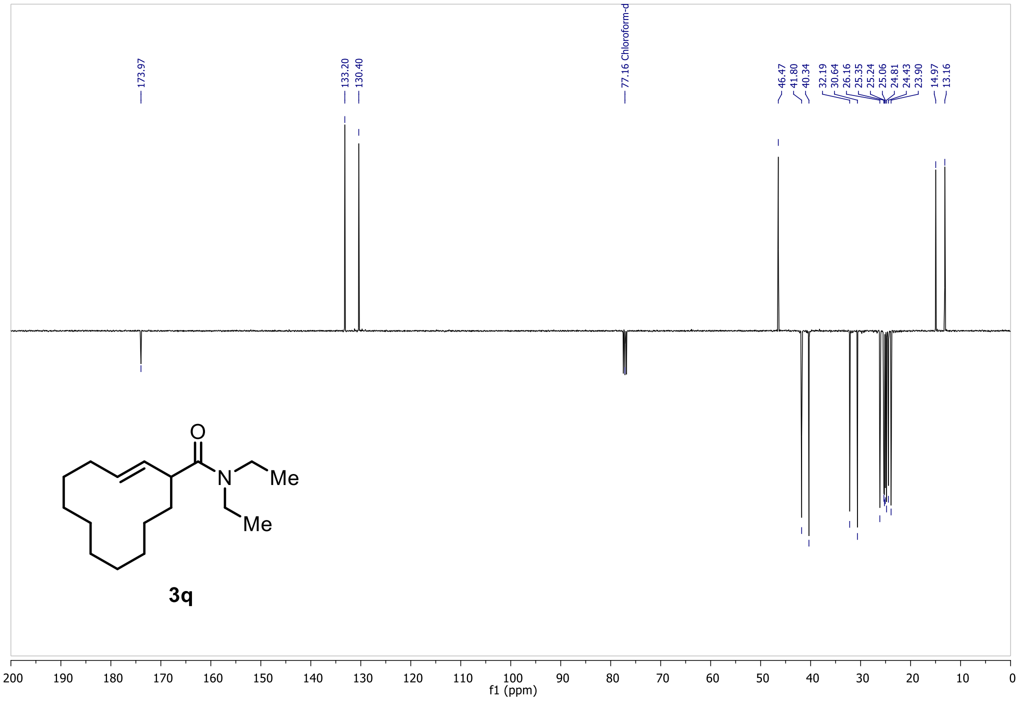

(*E*)-*N*,*N*-Diethyldodec-3-enamide (3r)

^{1}H NMR (400 MHz, $CDCl_3$):

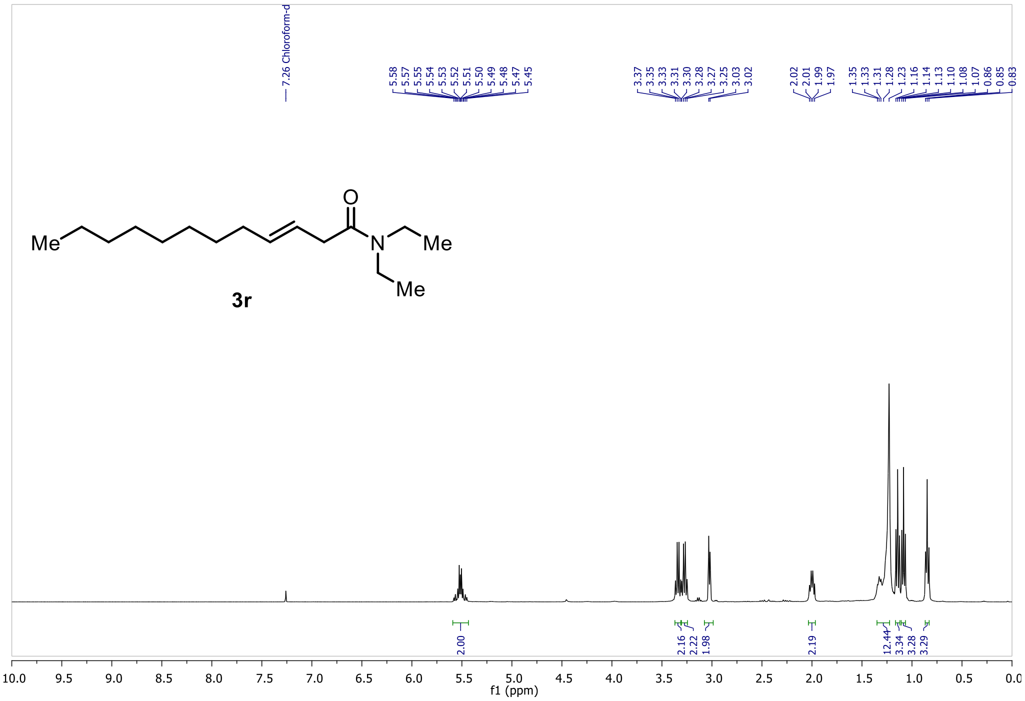

$^{13}C\{^{1}H\}$ NMR (101 MHz, $CDCl_3$):

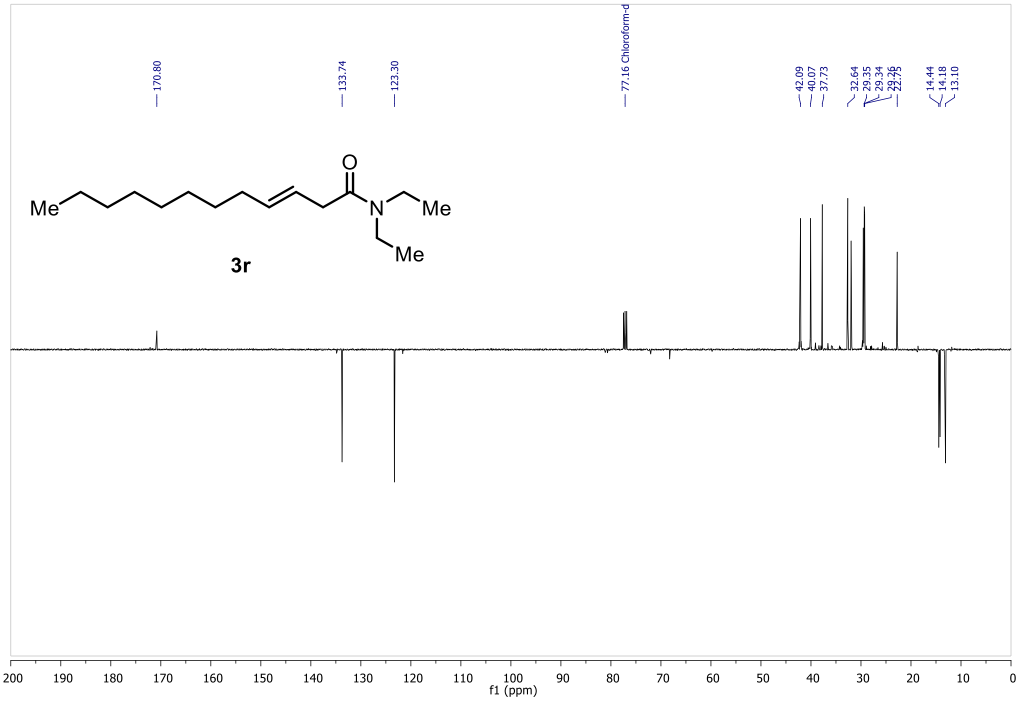

(*Z*)-*N*,*N*-Diethyldodec-3-enamide (3r'')

^{1}H NMR (400 MHz, $CDCl_3$):

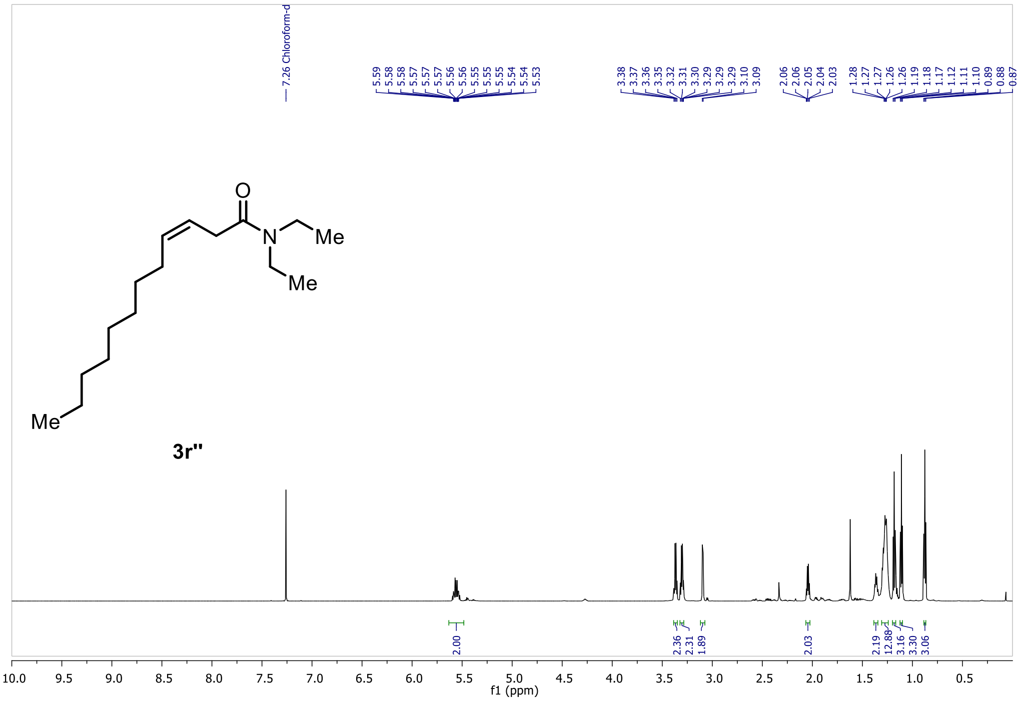

^{13}C{^{1}H} NMR (101 MHz, $CDCl_3$):

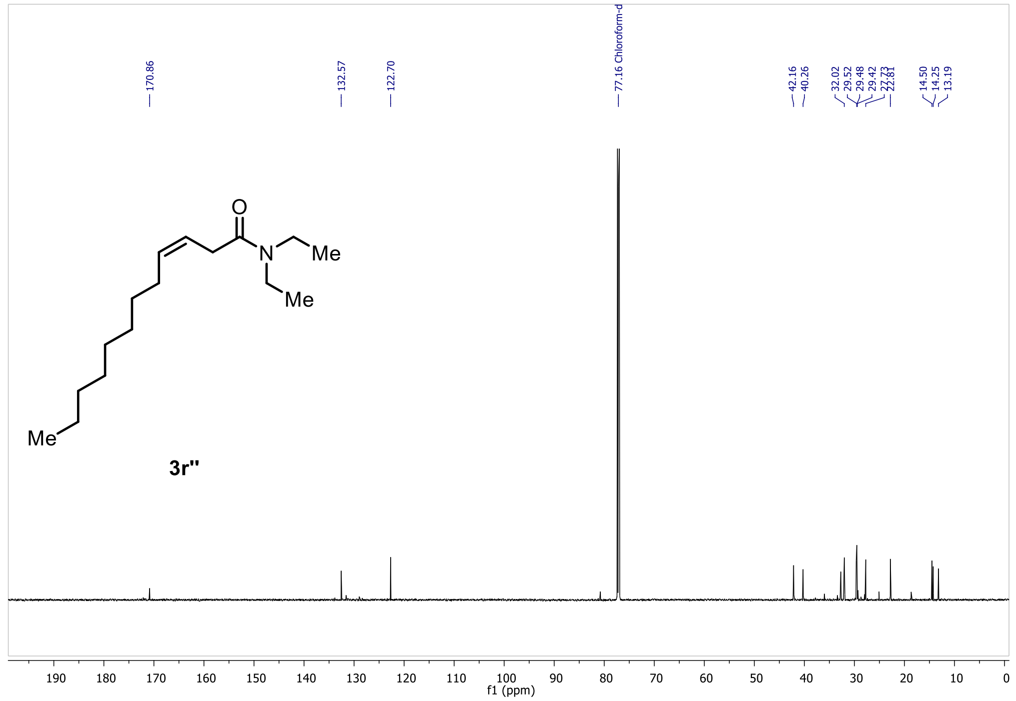

(*E*)-*N*,*N*-Diethyldec-3-enamide (3s)

^{1}H NMR (400 MHz, $CDCl_3$):

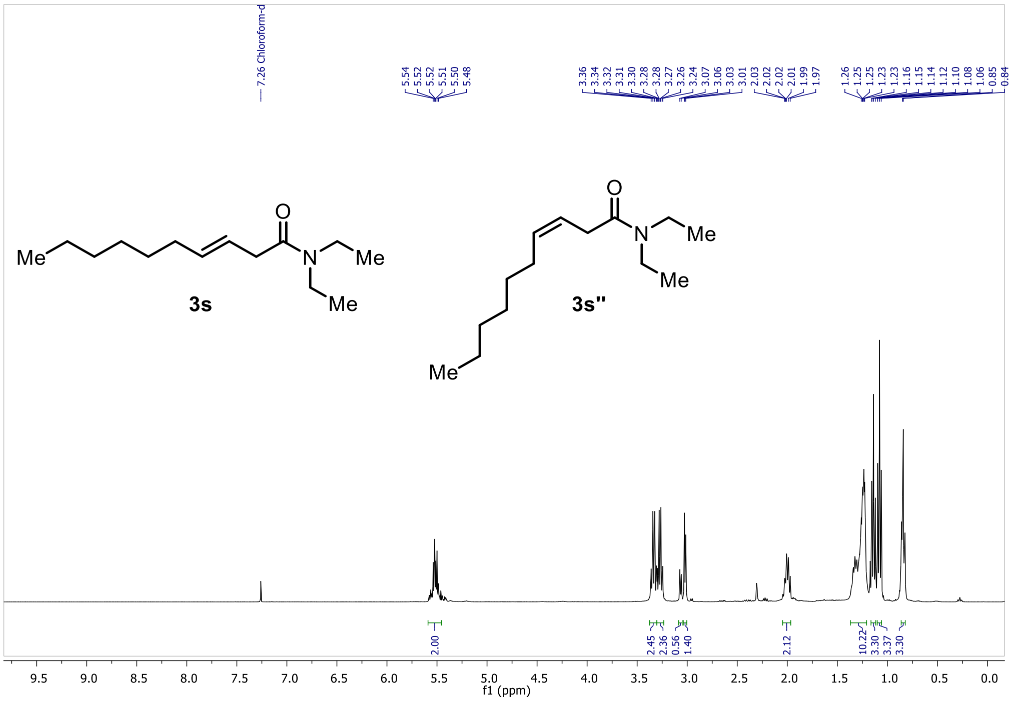

^{13}C{^{1}H} NMR (101 MHz, $CDCl_3$):

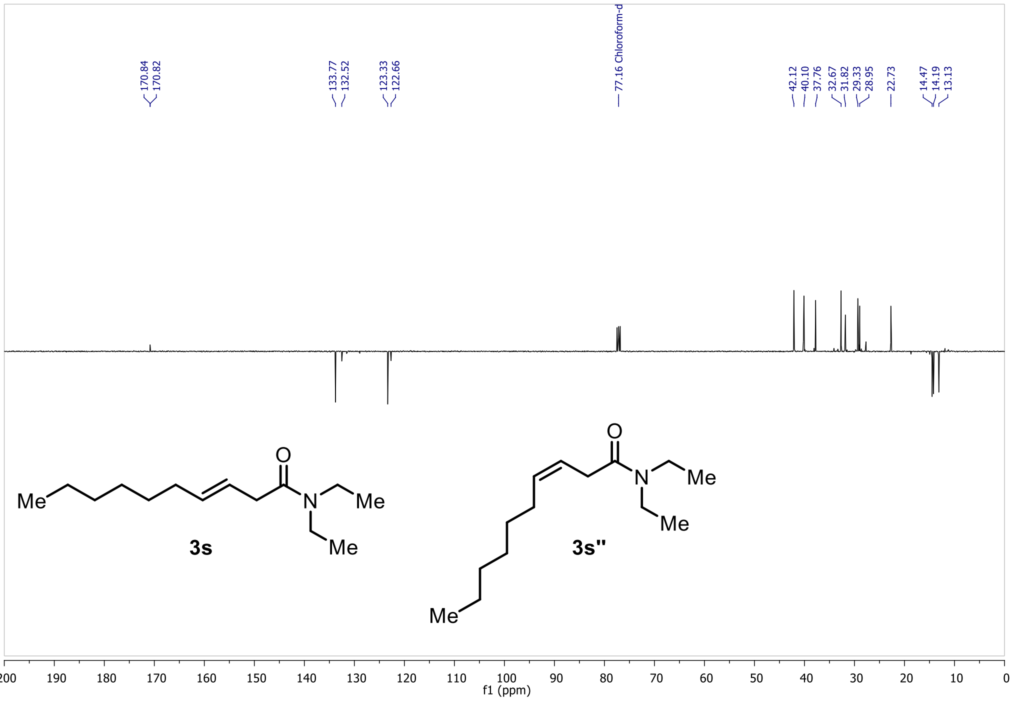

(*E*)-*N*,*N*-Diethylhex-3-enamide (3t)

^{1}H NMR (600 MHz, CDCl$_3$):

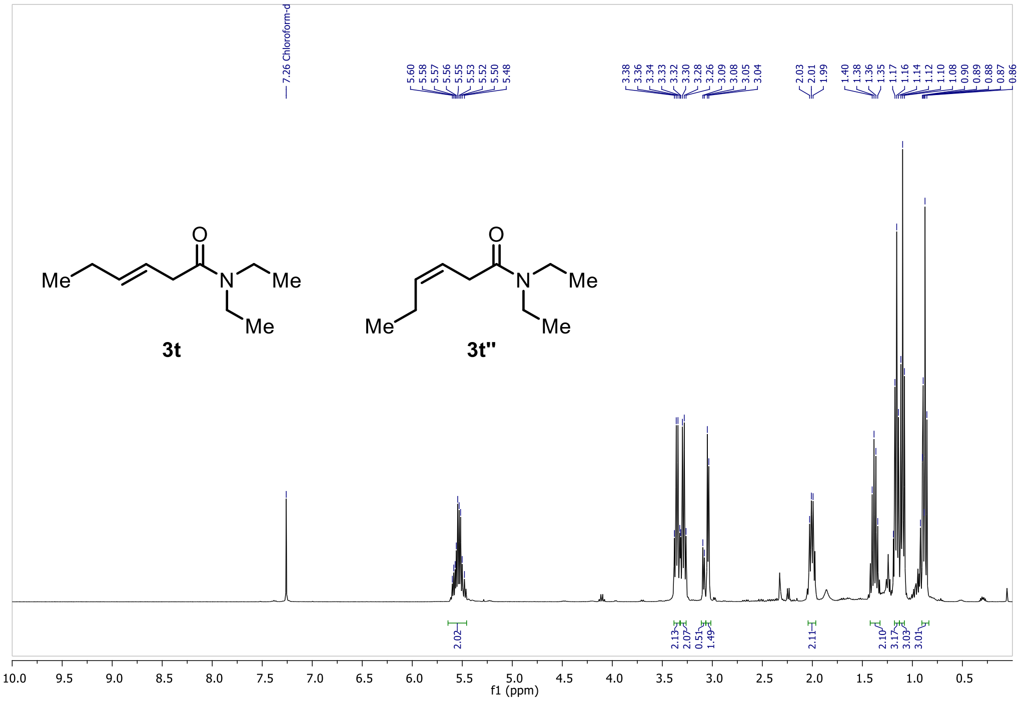

^{13}C{^{1}H} NMR (151 MHz, CDCl$_3$):

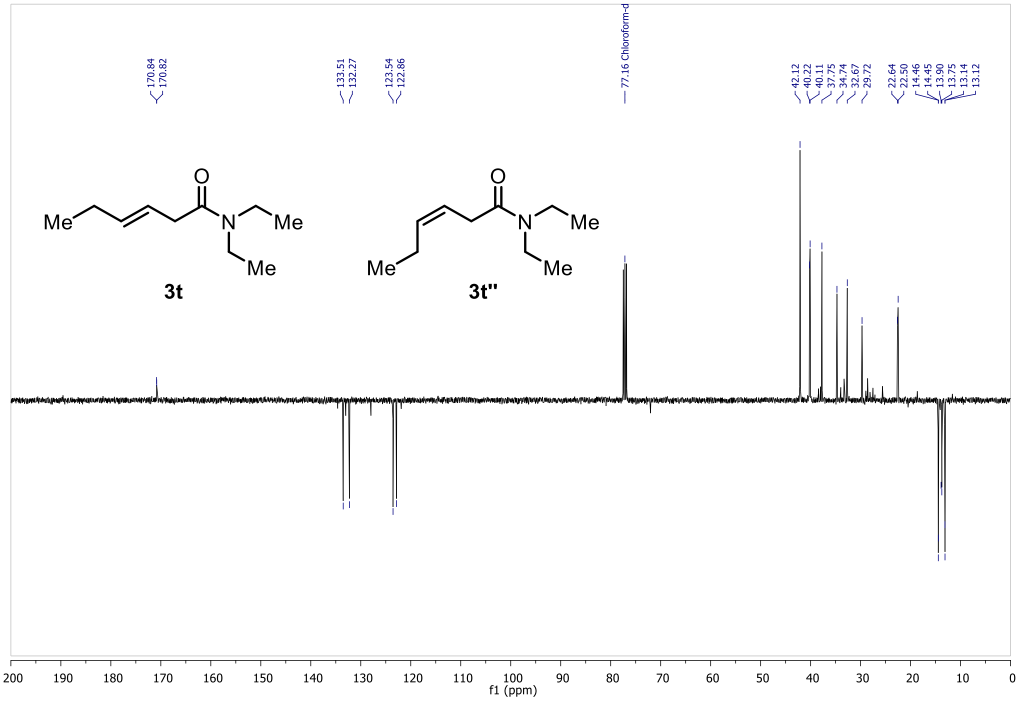

(*E*)-*N*,*N*-Diethylpent-3-enamide (3u)

^{1}H NMR (600 MHz, $CDCl_3$):

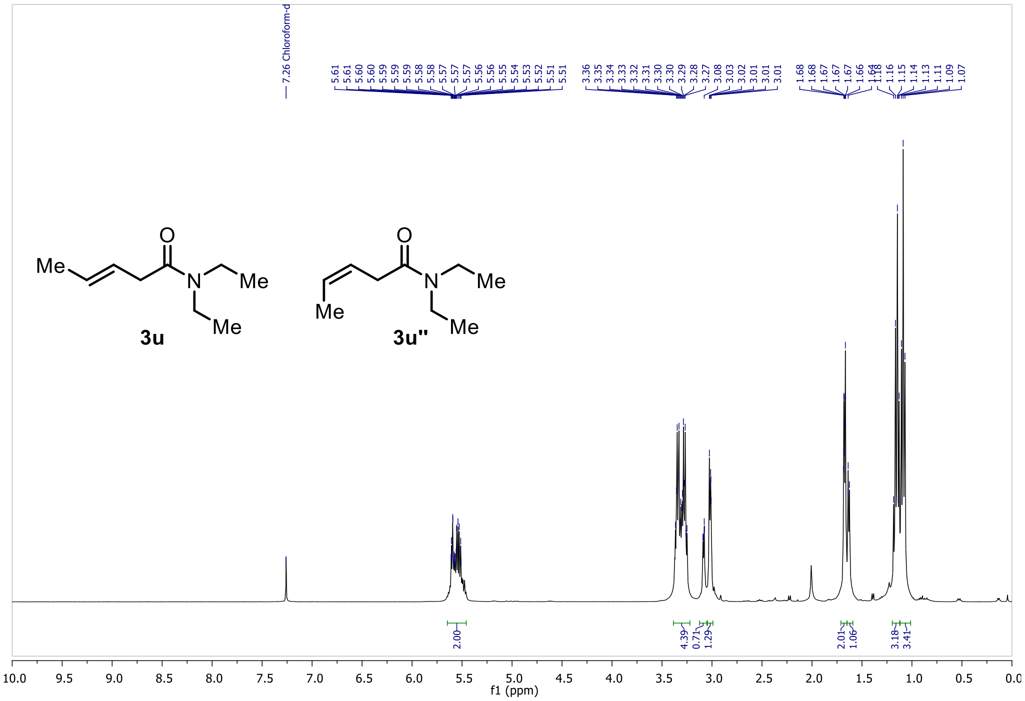

$^{13}C\{^{1}H\}$ NMR (151 MHz, $CDCl_3$):

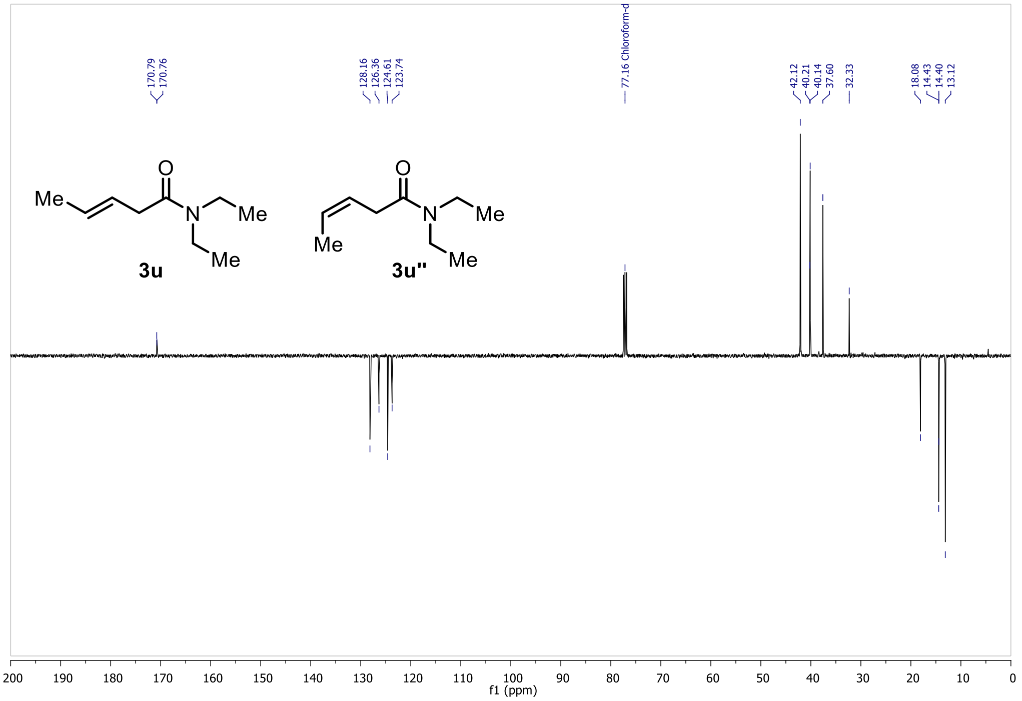

(*E*)-*N*,*N*,2-Triethylpent-3-enamide (3v)

^{1}H NMR (700 MHz, $CDCl_3$):

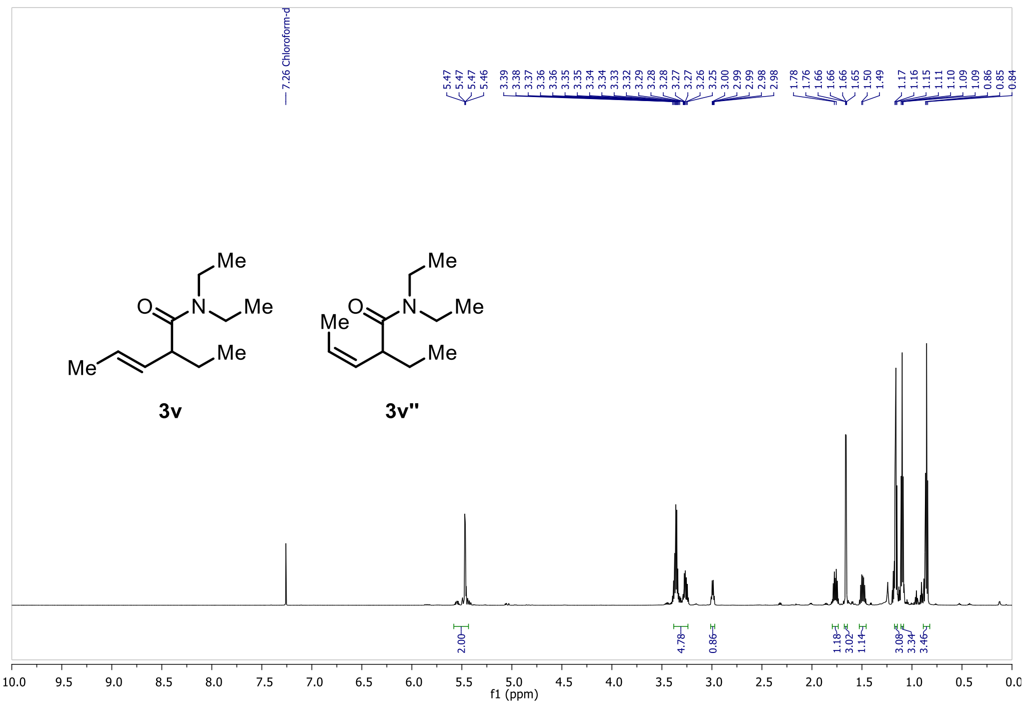

$^{13}C\{^{1}H\}$ NMR (151 MHz, $CDCl_3$):

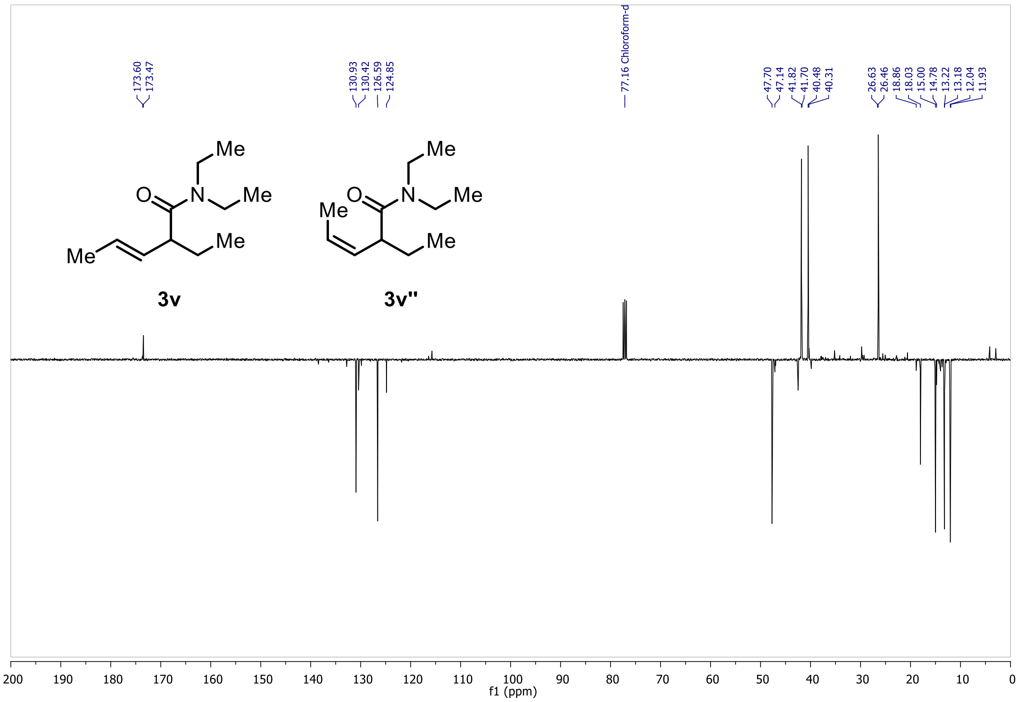

(*E*)-*N*,*N*-Diethyl-5-phenylpent-3-enamide (3w)

^{1}H NMR (700 MHz, $CDCl_3$):

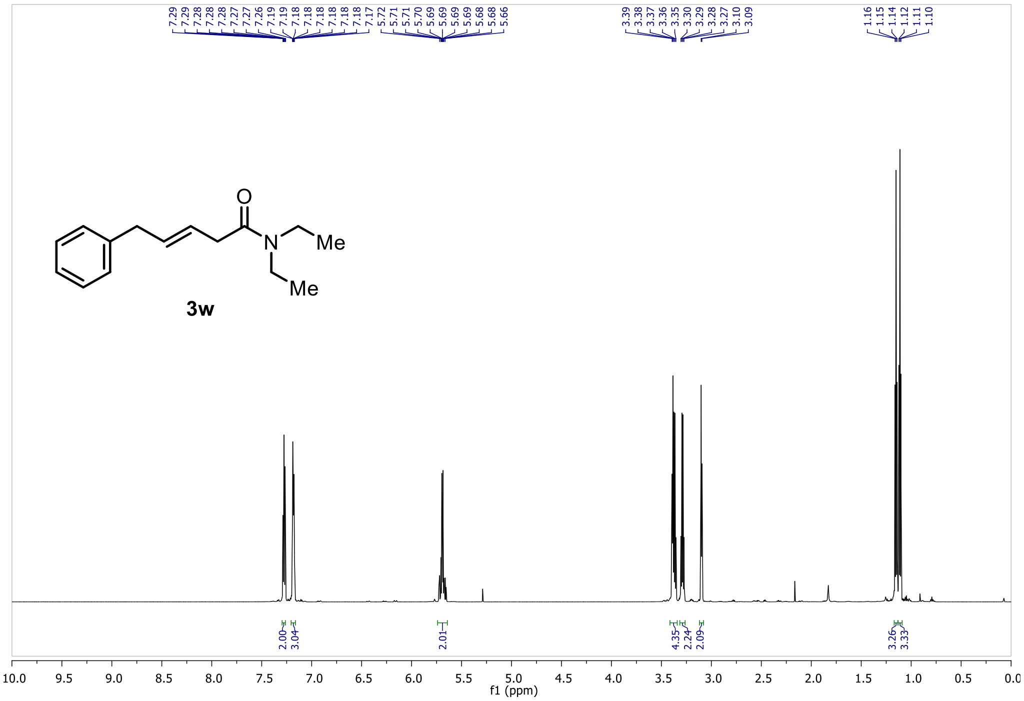

^{13}C{^{1}H} NMR (151 MHz, $CDCl_3$):

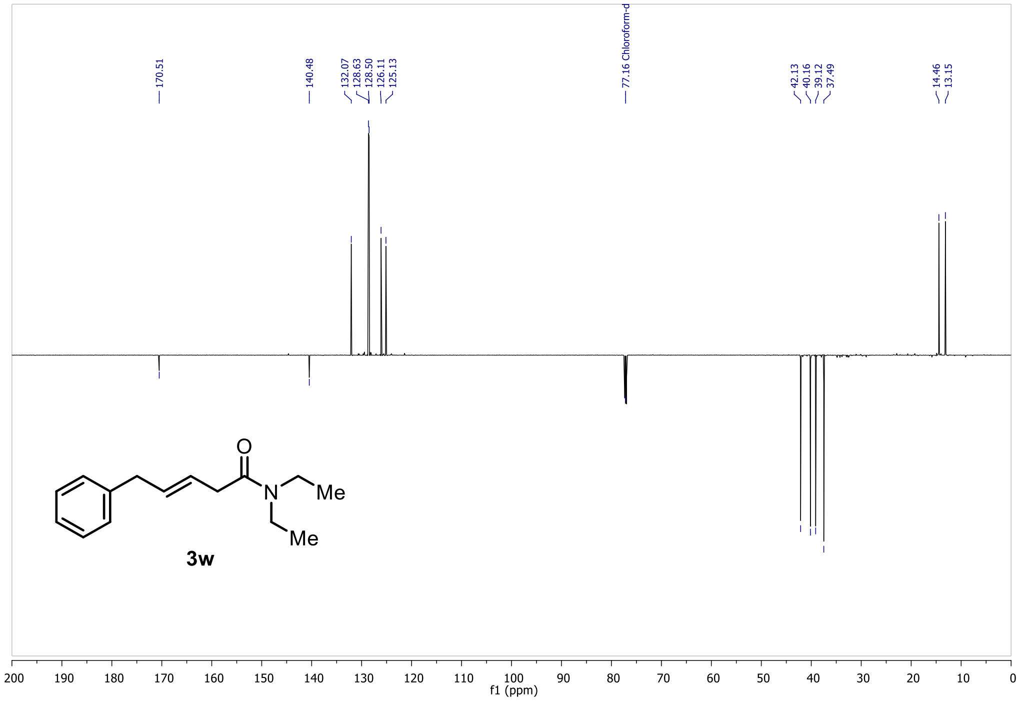

(*Z*)-*N*,*N*-Diethyl-5-phenylpent-3-enamide (3w'')

^{1}H NMR (700 MHz, $CDCl_3$):

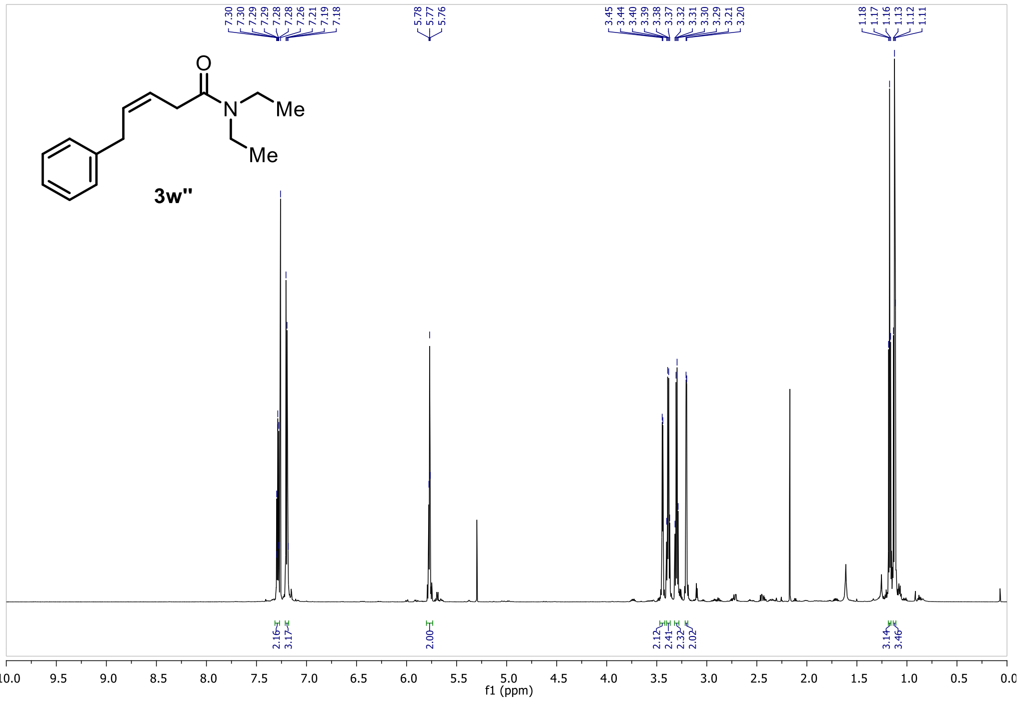

^{13}C{^{1}H} NMR (151 MHz, $CDCl_3$):

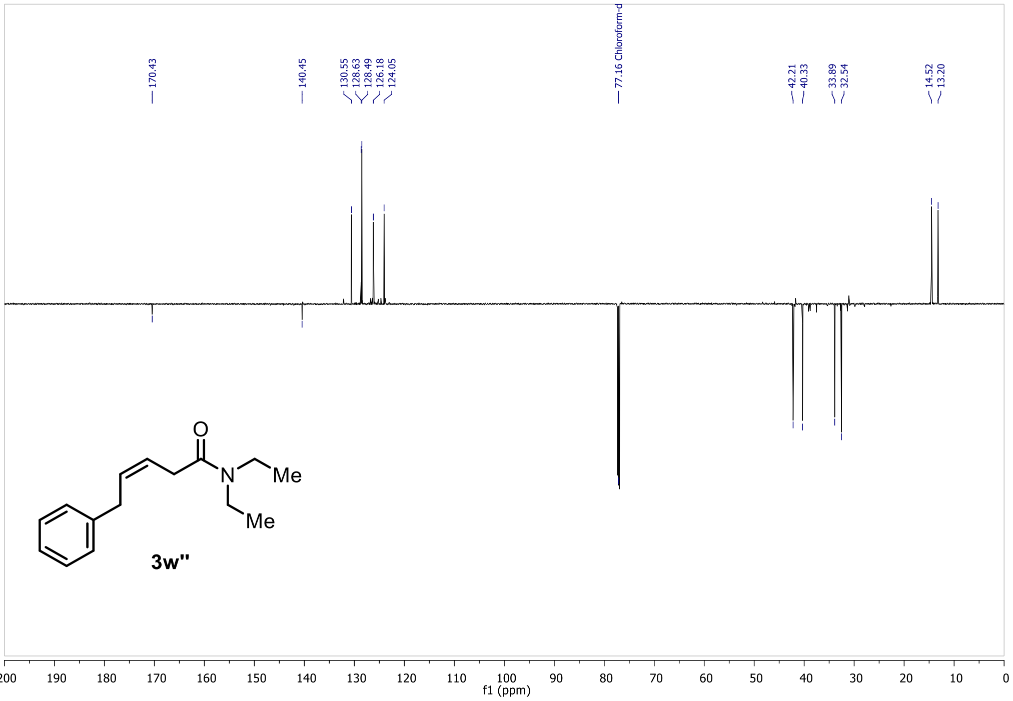

(*E*)-*N*,*N*-Diethyl-6-phenylhex-3-enamide (3x)

^{1}H NMR (700 MHz, $CDCl_3$):

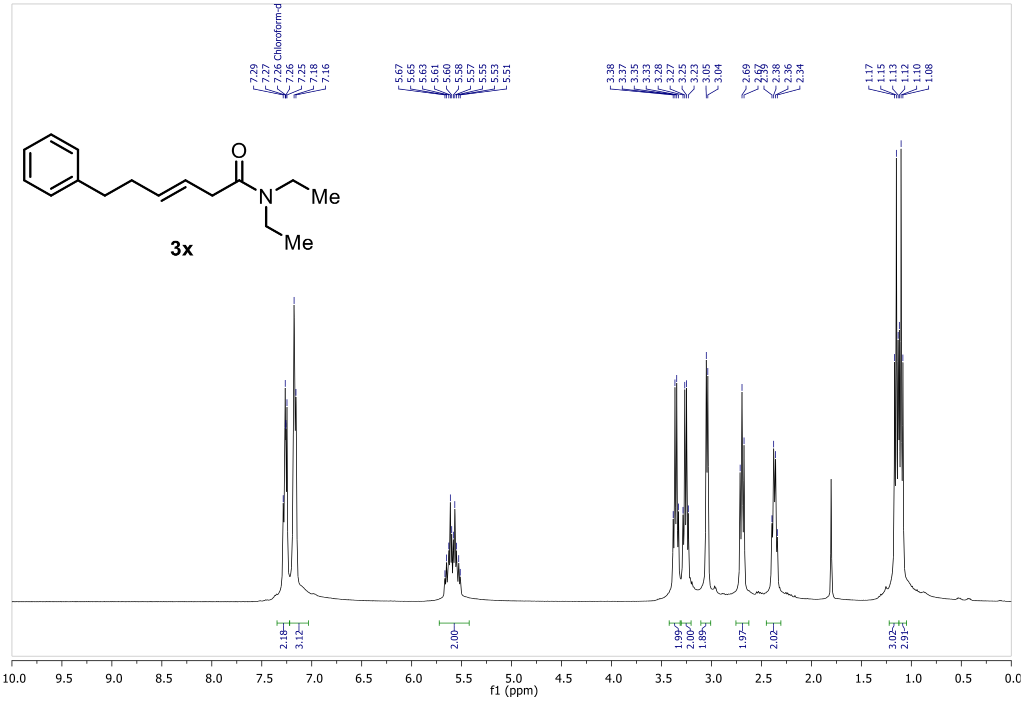

^{13}C{^{1}H} NMR (151 MHz, $CDCl_3$):

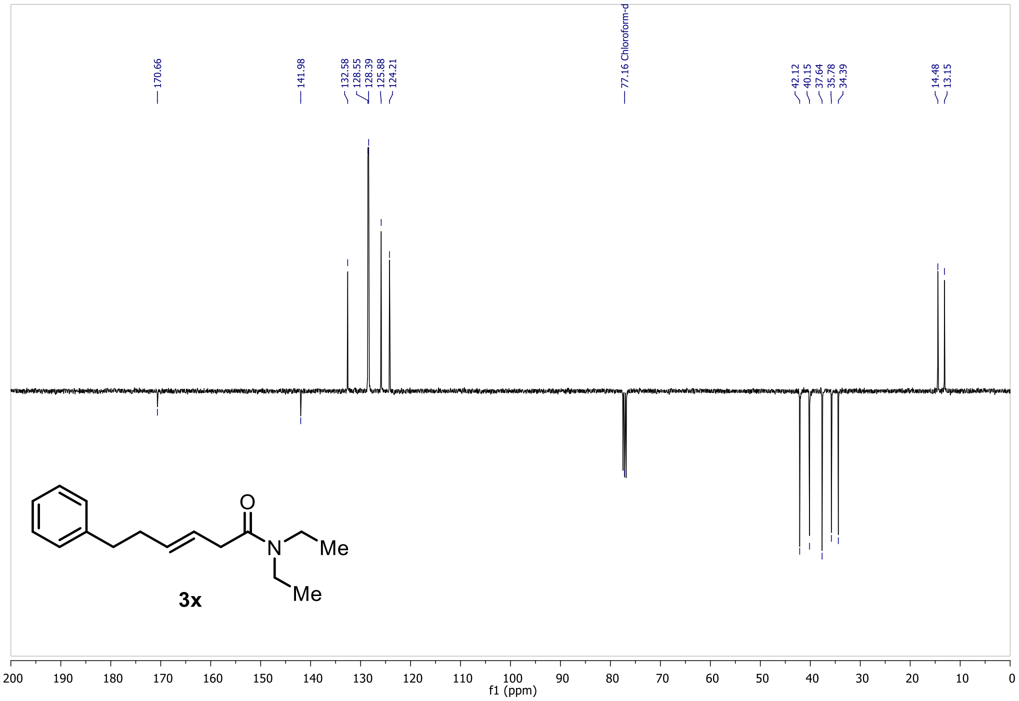

(*Z*)-*N*,*N*-Diethyl-6-phenylhex-3-enamide (3x'')

^{1}H NMR (700 MHz, CDCl$_3$):

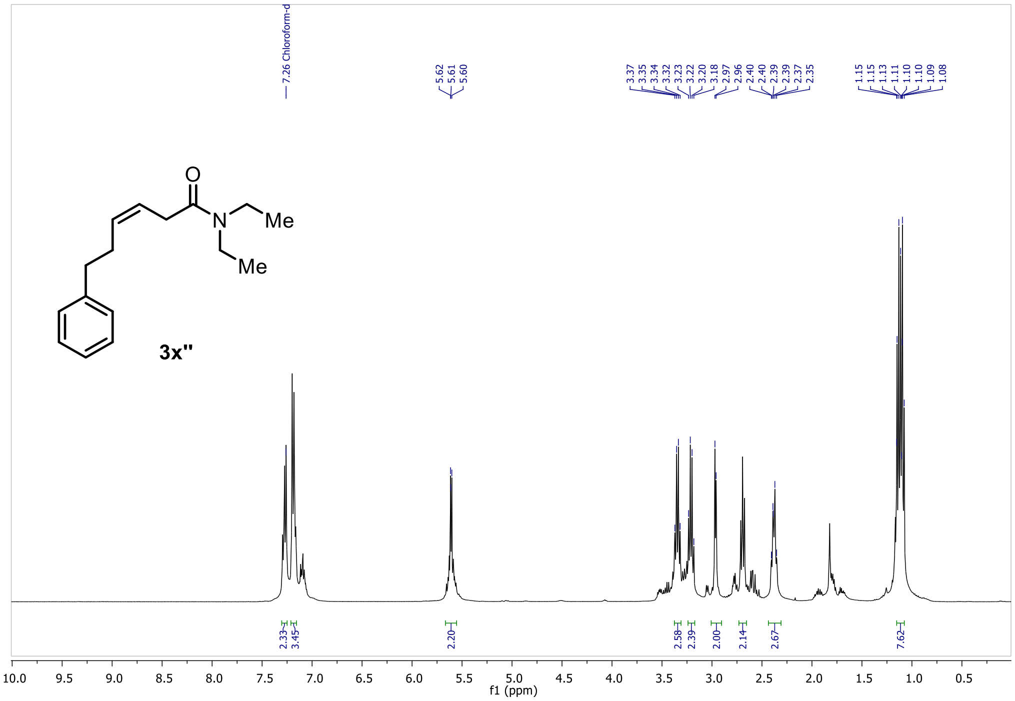

^{13}C{^{1}H} NMR (151 MHz, CDCl$_3$):

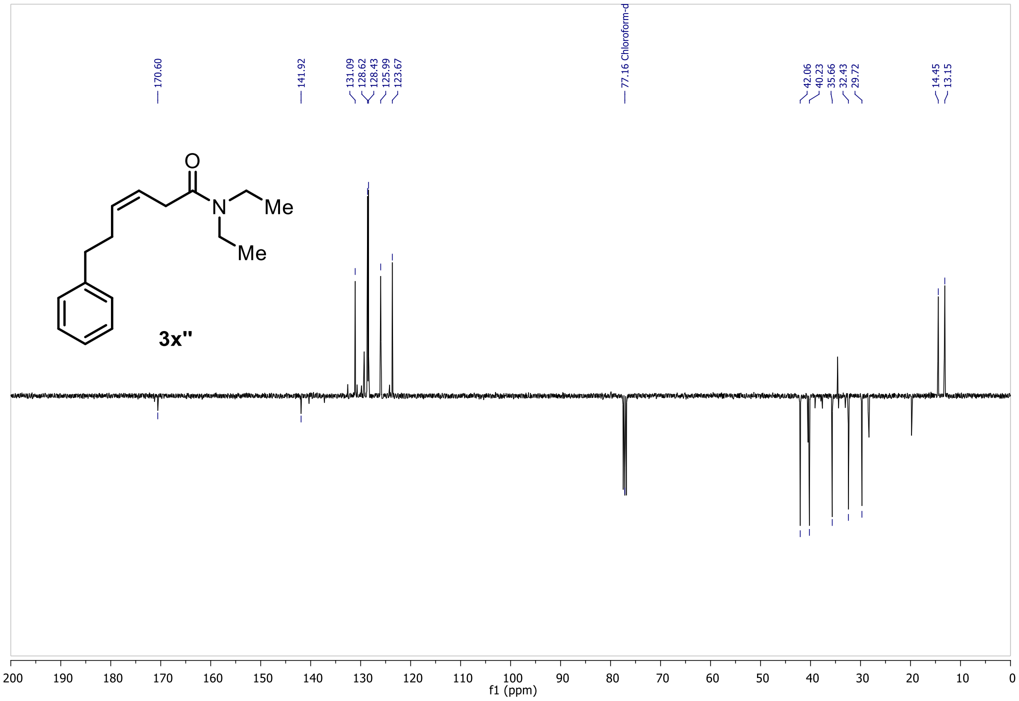

(*E*)-*N*,*N*-Diethyl-6-phenylhex-3-enamide (3y)

^{1}H NMR (400 MHz, $CDCl_3$):

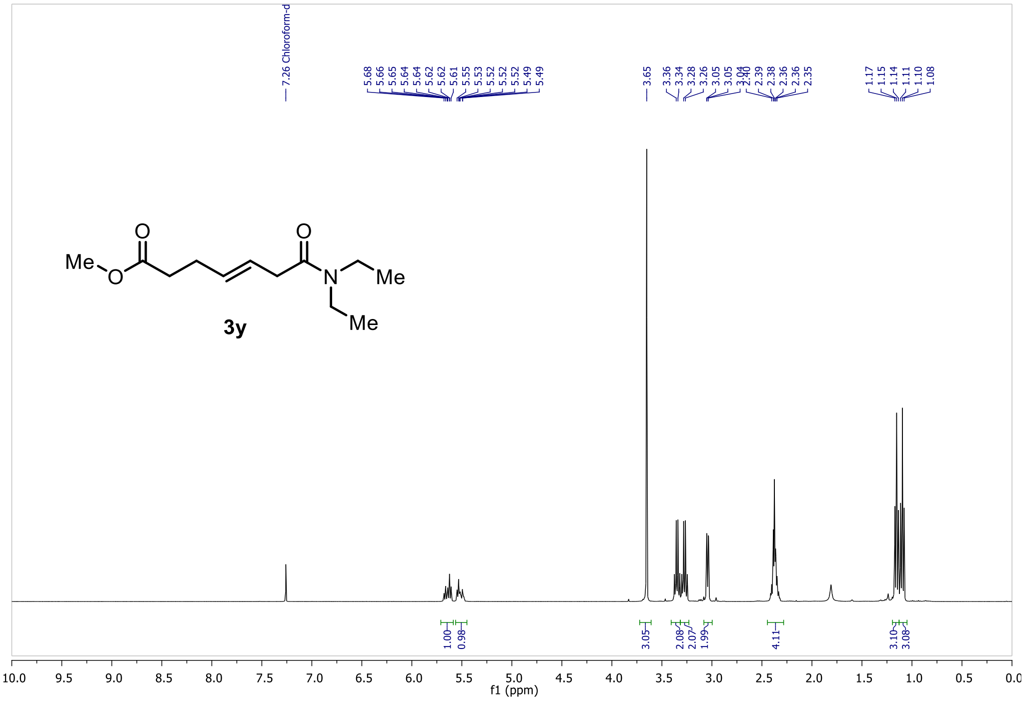

^{13}C{^{1}H} NMR (101 MHz, $CDCl_3$):

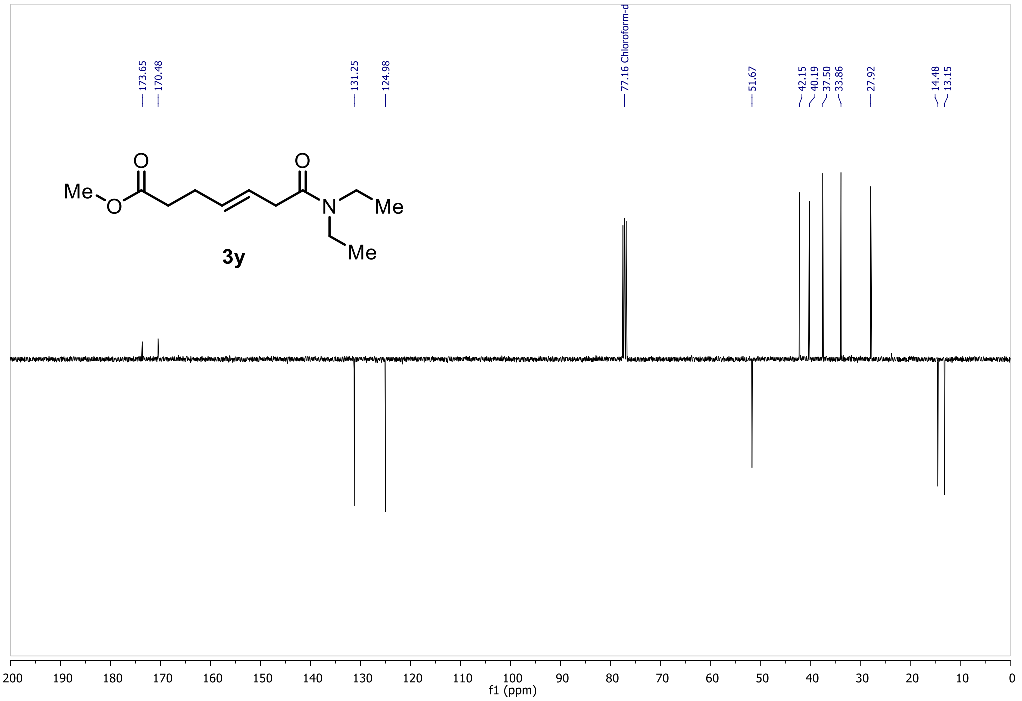

(*Z*)-*N*,*N*-Diethyl-6-phenylhex-3-enamide (3y'')

^{1}H NMR (400 MHz, $CDCl_3$):

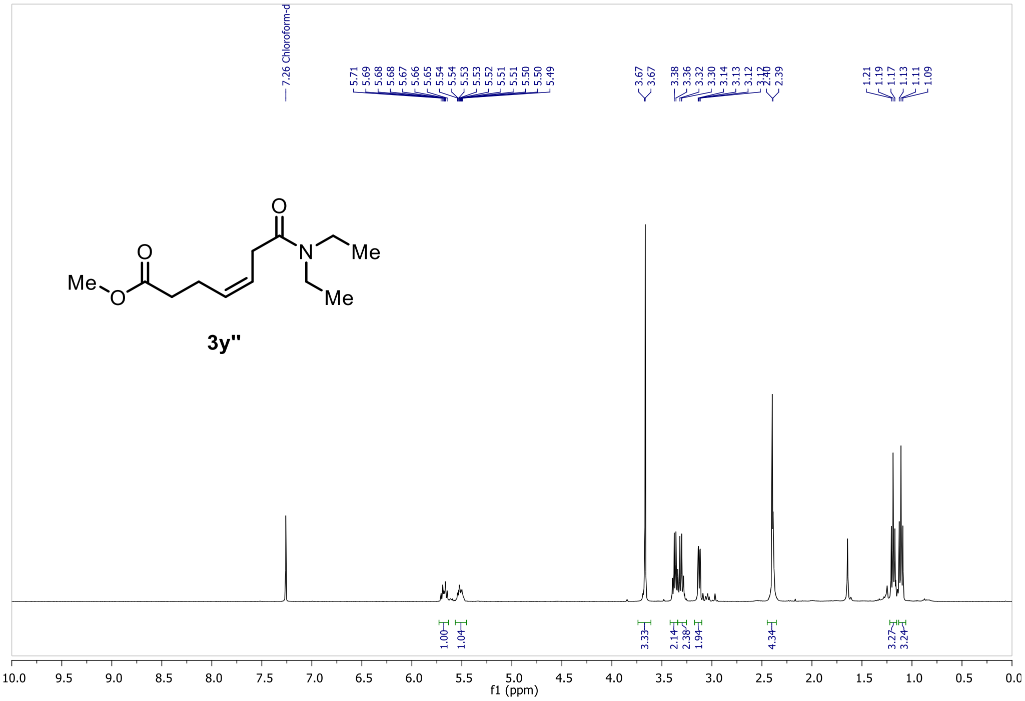

^{13}C{^{1}H} NMR (101 MHz, $CDCl_3$):

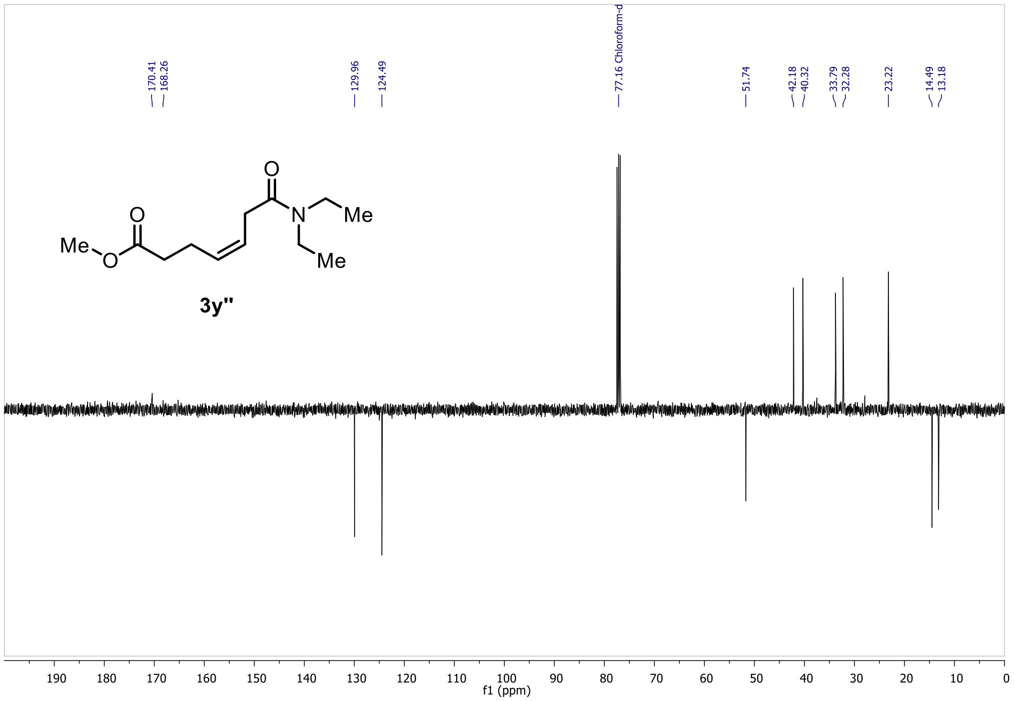

(*E*)-7-Bromo-*N*,*N*-diethylhept-3-enamide (3z)

^{1}H NMR (400 MHz, $CDCl_3$):

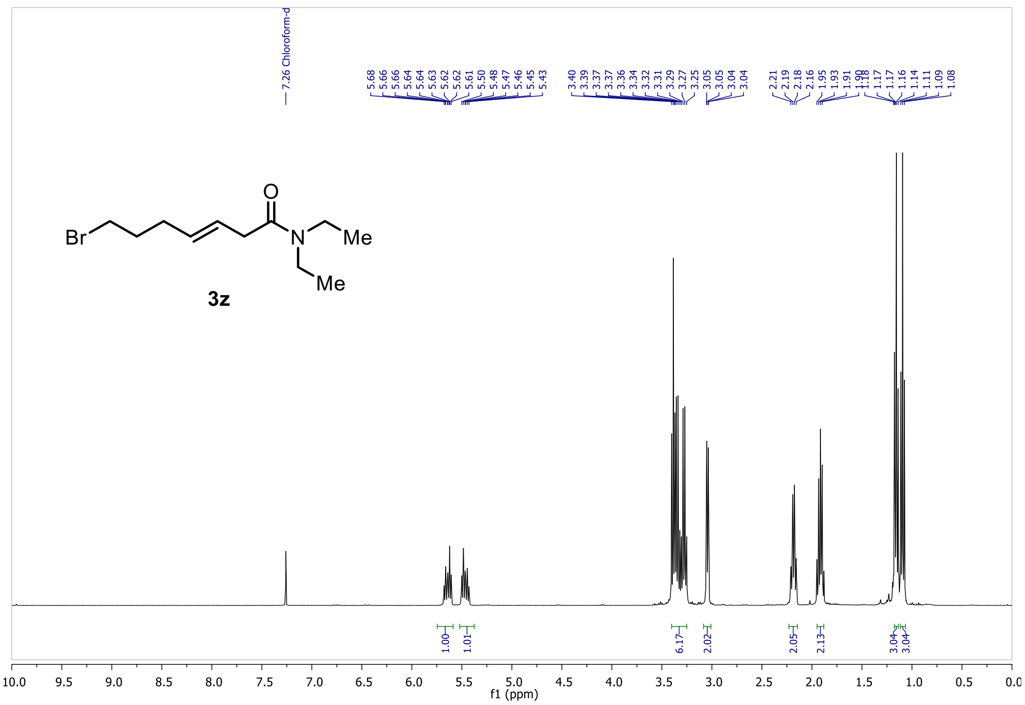

$^{13}C\{^{1}H\}$ NMR (101 MHz, $CDCl_3$):

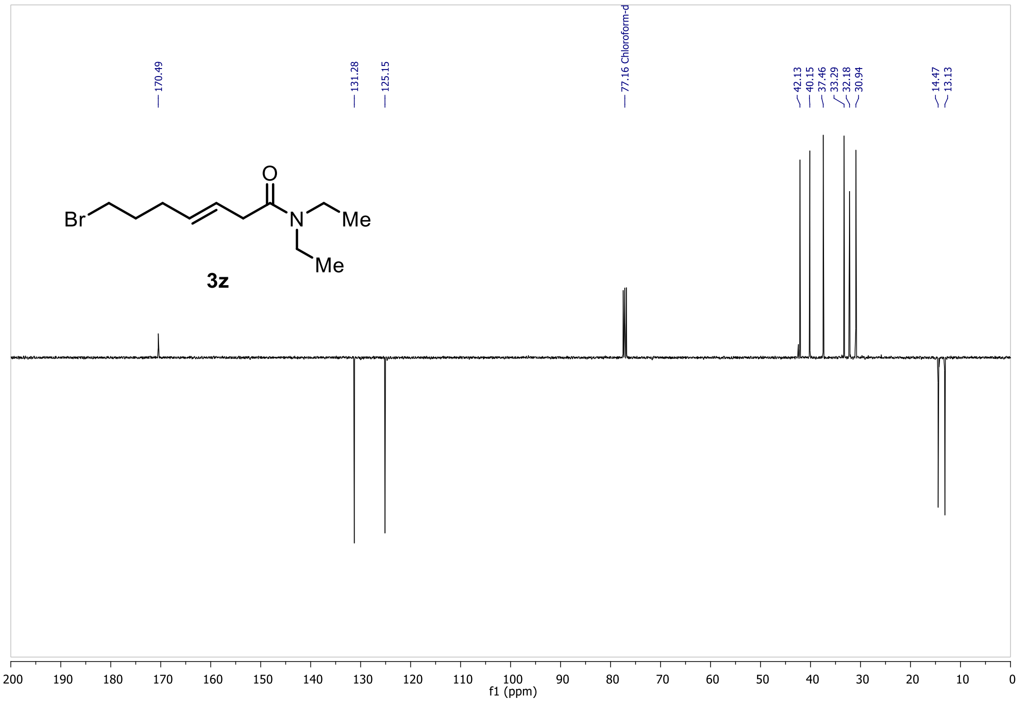

(*Z*)-7-Bromo-*N*,*N*-diethylhept-3-enamide (3z'')

^{1}H NMR (400 MHz, $CDCl_3$):

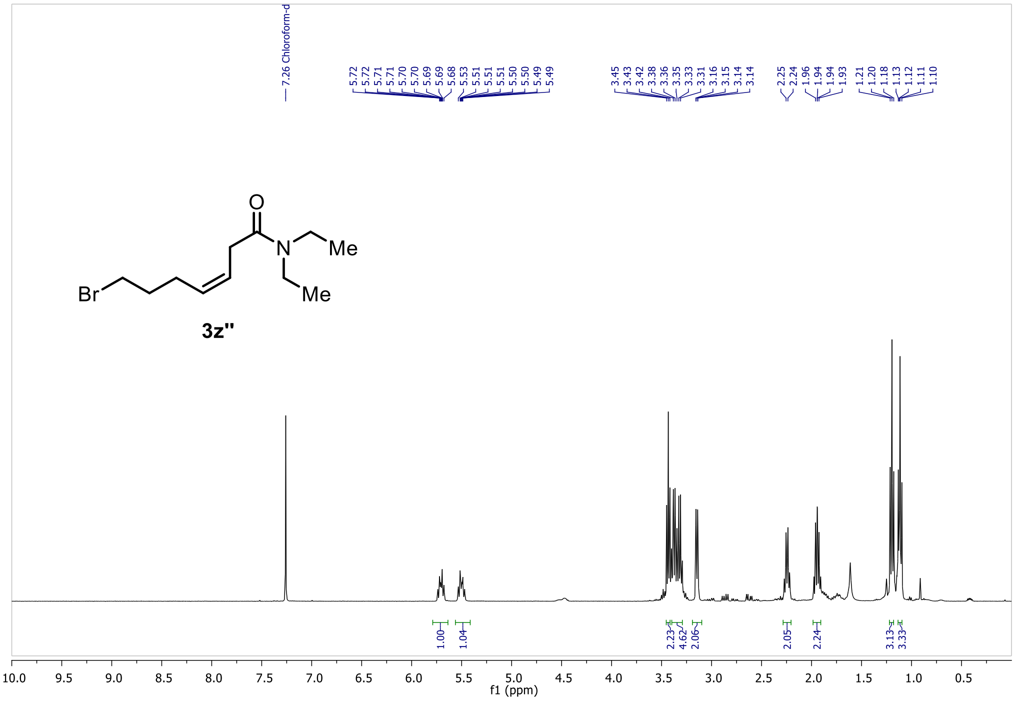

^{13}C{^{1}H} NMR (101 MHz, $CDCl_3$):

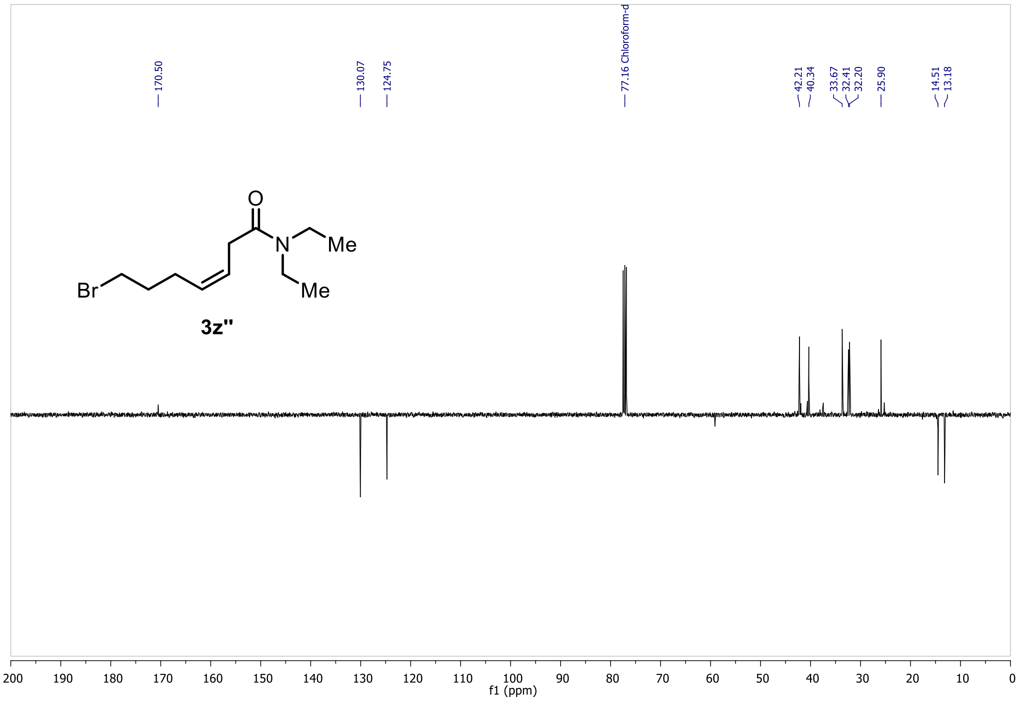

(*E*)-8-(1,3-Dioxoisoindolin-2-yl)-*N*,*N*-diethyloct-3-enamide (3aa)

^{1}H NMR (400 MHz, $CDCl_3$):

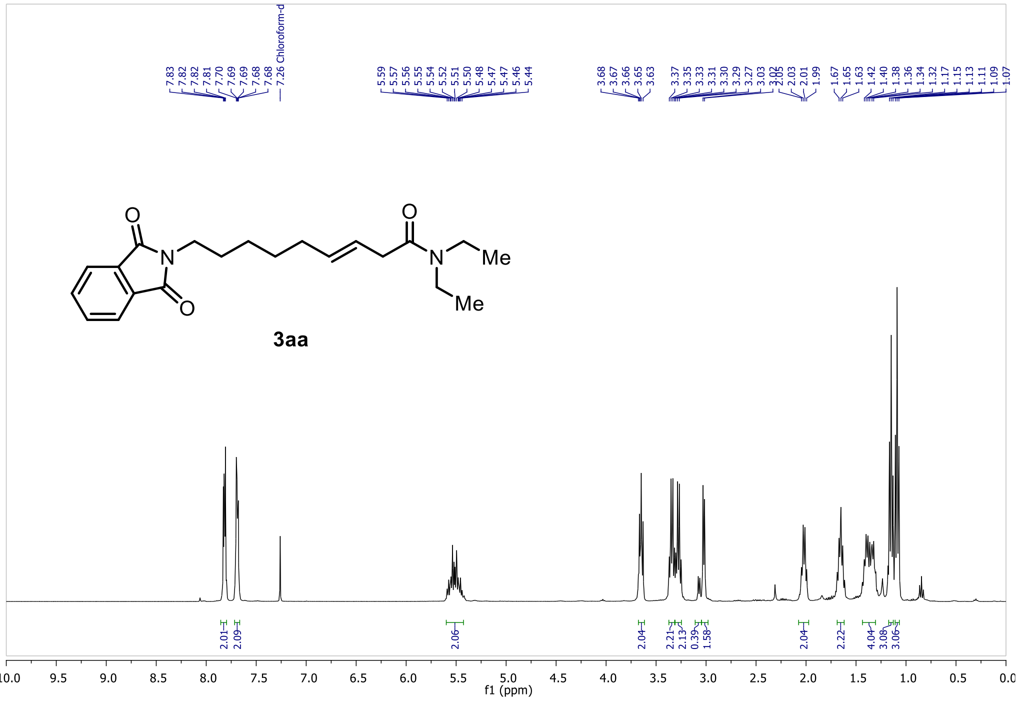

$^{13}C\{^{1}H\}$ NMR (101 MHz, $CDCl_3$):

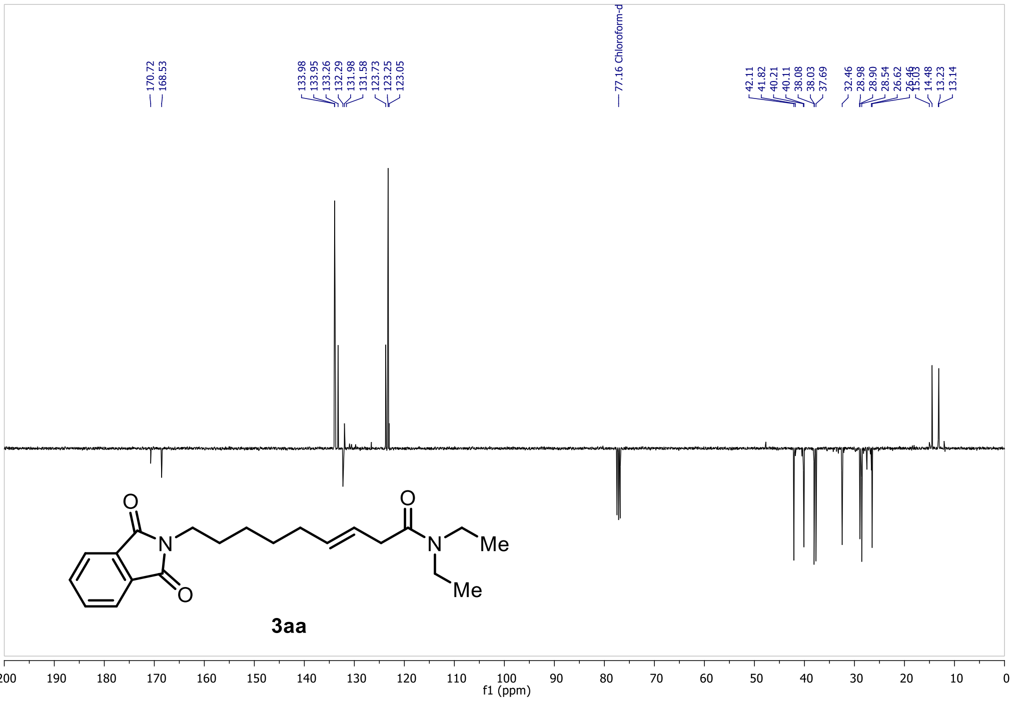

(*E*)-1-(Pyrrolidin-1-yl)-4-((2,2,6,6-tetramethylpiperidin-1-yl)oxy)dodec-2-en-1-one (4a)

^{1}H NMR (400 MHz, $CDCl_3$):

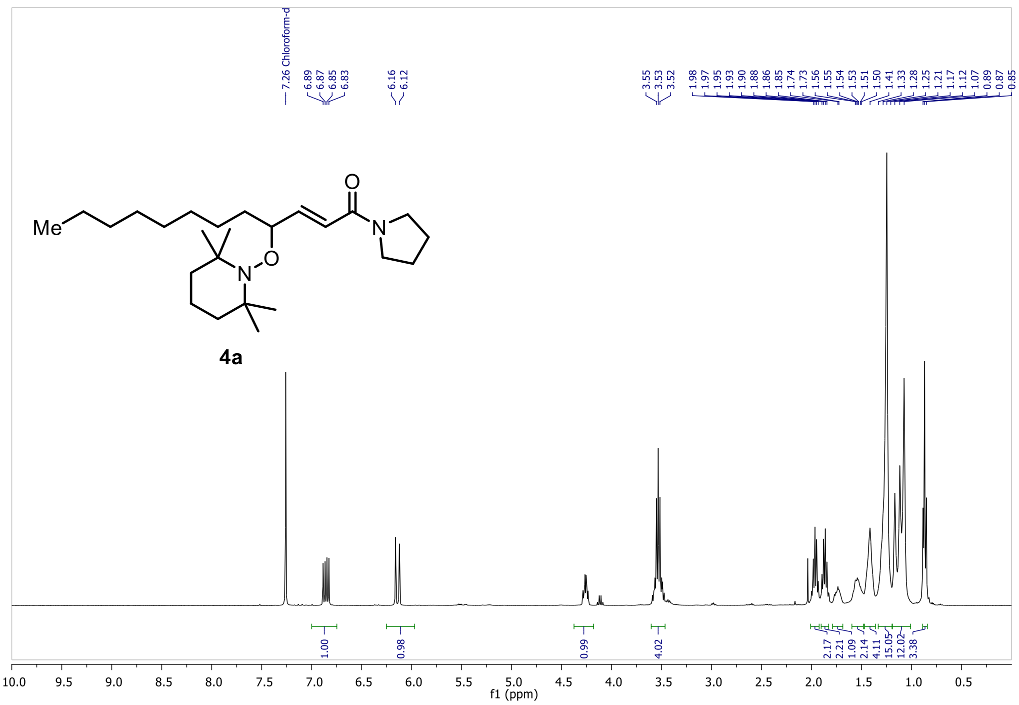

^{13}C{^{1}H} NMR (101 MHz, $CDCl_3$):

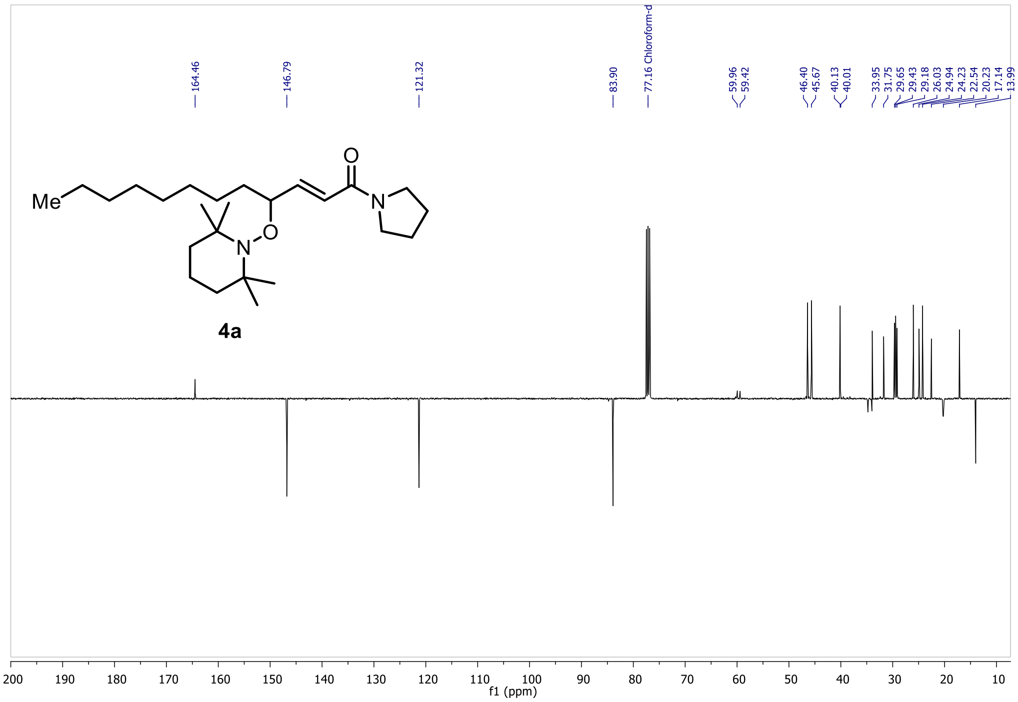

(*E*)-*N*,*N*-Diethyl-4-((2,2,6,6-tetramethylpiperidin-1-yl)oxy)hept-2-enamide (4b)

^{1}H NMR (400 MHz, $CDCl_3$):

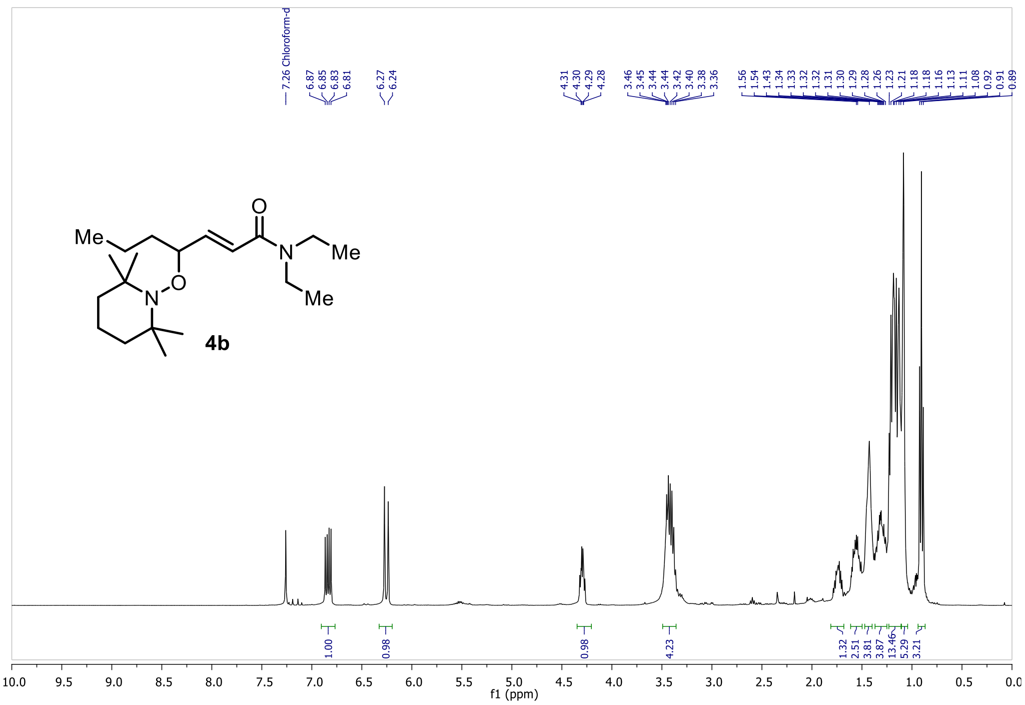

^{13}C{^{1}H} NMR (101 MHz, $CDCl_3$):

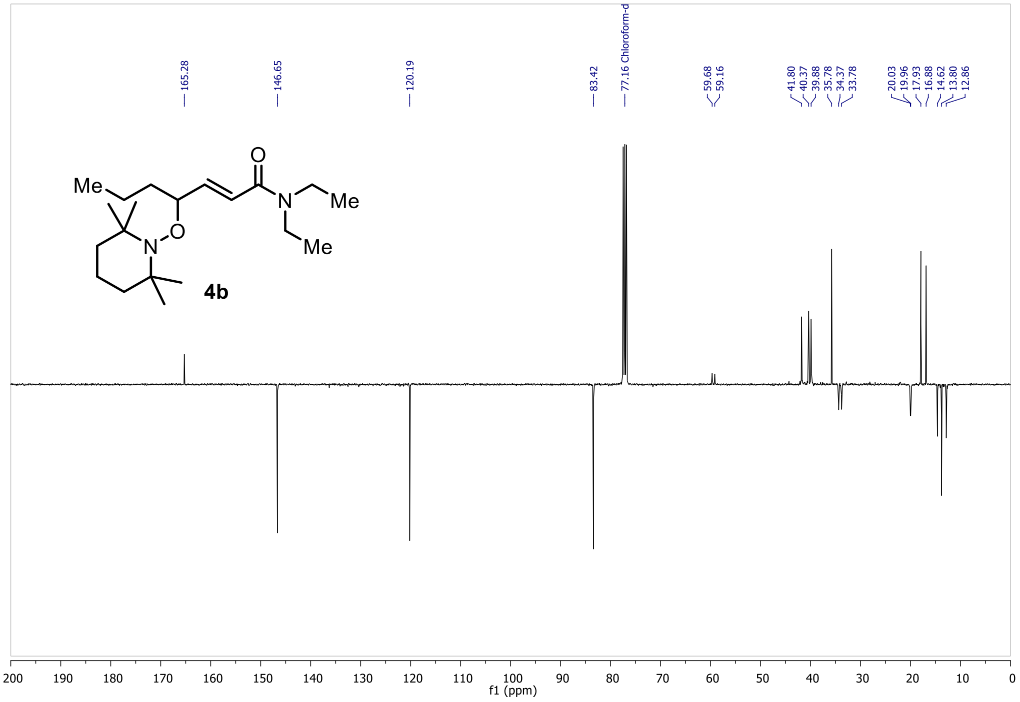

(*Z*)-*N*,*N*-Diethyl-3-fluorohex-2-enamide (6a)

^{1}H NMR (600 MHz, $CDCl_3$):

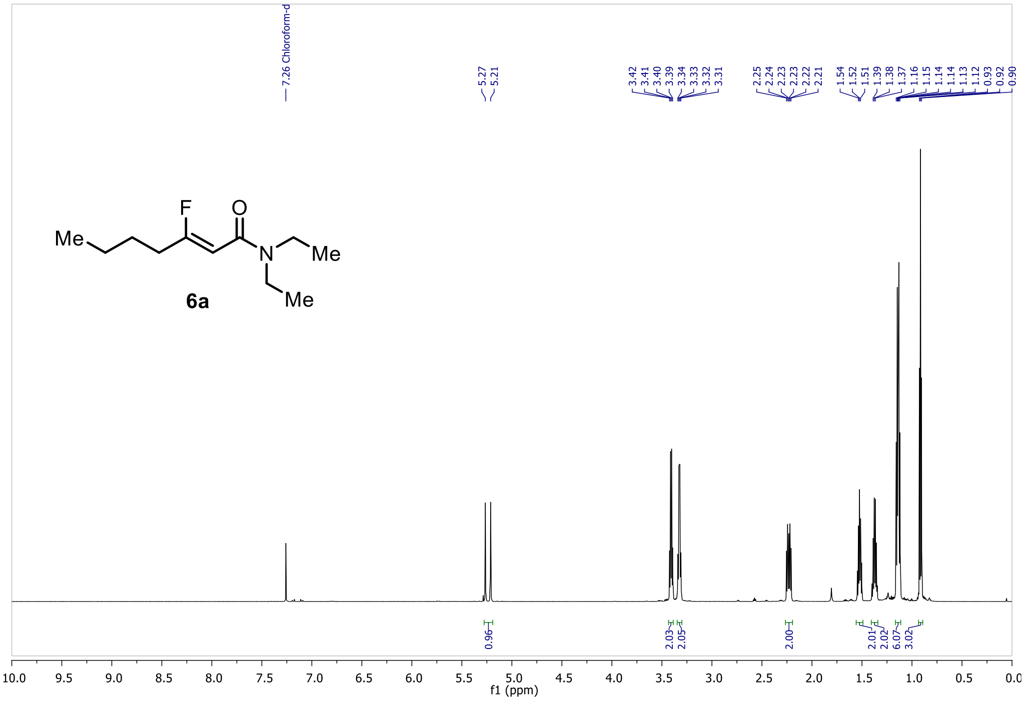

$^{13}C\{^{1}H\}$ NMR (151 MHz, $CDCl_3$):

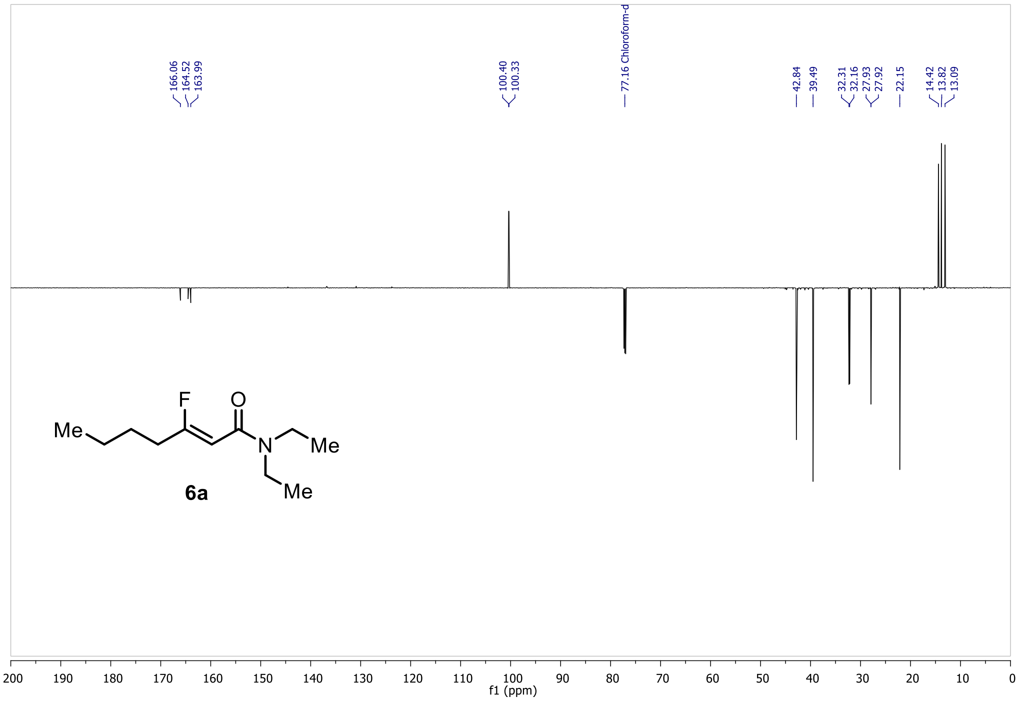

^{19}F NMR (659 MHz, CDCl$_3$):

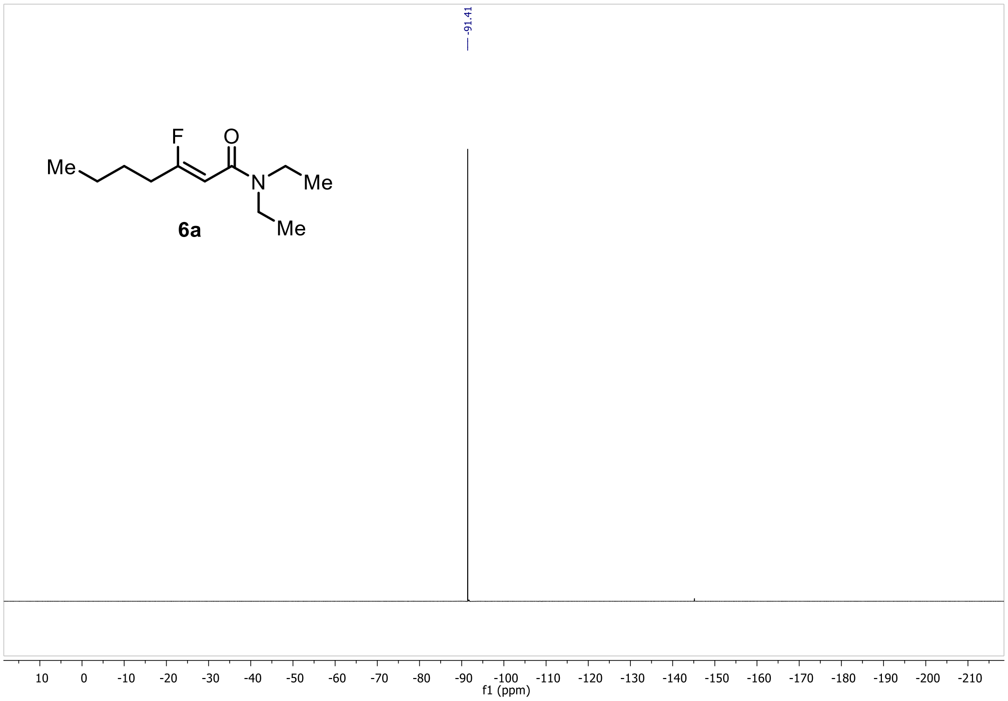

9. References

[1] D. Zuo, Q. Wang, L. Liu, T. Huang, M. Szostak, T. Chen, *Angew. Chem. Int. Ed.* **2022**, *61*, e202202794.
[2] J. Morgan, A. Greenberg, J. F. Liebman, *Struct. Chem.* **2012**, *23*, 197–199.
[3] N. Radhoff, A. Studer, *Nat. Commun.* **2022**, *13,* 3083.
[4] R. Logeswaran, M. Jeganmohan, *Org. Lett.* **2023**, *25*, 6284–6289.
[5] S. Zheng, W. Wang, W. Yuan, *J. Am. Chem. Soc.* **2022**, *144*, 17776–17782.
